# Leisure-time and occupational physical activity and risk of cardiovascular disease incidence: a systematic-review and dose-response meta-analysis of prospective cohort studies

**DOI:** 10.1186/s12966-024-01593-8

**Published:** 2024-04-24

**Authors:** Asma Kazemi, Sepideh Soltani, Dagfinn Aune, Elham Hosseini, Zeinab Mokhtari, Zahra Hassanzadeh, Ahmad Jayedi, Francisco Pitanga, Masoumeh Akhlaghi

**Affiliations:** 1 Nutrition Research Center, School of Nutrition and Food Sciences, Shiraz University of Medical Sciences, Shiraz, Iran; 2 Cardiovascular Research Center, Shahid Sadoughi University of Medical Sciences, Yazd, Iran; 3 Department of Epidemiology and Biostatistics, School of Public Health, Imperial College London, London, UK; 4 Department of Nutrition, Oslo New University College, Oslo, Norway; 5 Department of Endocrinology, Morbid Obesity and Preventive Medicine, Oslo University Hospital, Oslo, Norway; 6 Nutrition and Food Security Research Center, Isfahan University of Medical Sciences, Isfahan, Iran; 7 Social Determinants of Health Research Center, Semnan University of Medical Sciences, Semnan, Iran; 8 The Federal University of Bahia, Salvador, Bahia Brazil; 9 Department of Community Nutrition, School of Nutrition and Food Sciences, Shiraz University of Medical Sciences, Razi Blvd, 7153675541 Shiraz, Iran

**Keywords:** Leisure-time physical activity, Occupational physical activity, Work physical activity, Cardiovascular disease

## Abstract

**Background and objective:**

Physical activity has benefits for the cardiovascular system, however, what levels and types of activity provide optimal cardiovascular health is unclear. We aimed to determine the level of physical activity that has the most benefits against cardiovascular diseases (CVD).

**Methods:**

PubMed, Scopus, and Web of Science were searched for prospective cohort studies on leisure-time (LTPA) or occupational physical activity (OPA) as the exposure and major types of CVD (total CVD, coronary heart disease [CHD], stroke, and atrial fibrillation [AF]) incidence as the outcome. Risk of bias of studies was evaluated using the ROBINS-I tool. Summary hazard ratios (HR) were calculated using random-effects pairwise model.

**Results:**

A total of 103 studies were included in the analysis. The highest versus the lowest LTPA was associated with a lower risk of overall CVD (HR = 0.81; 95% CI: 0.77–0.86), CHD (HR = 0.83; 0.79–0.88), and stroke (HR = 0.83; 0.79–0.88), but not AF (HR = 0.98; 0.92–1.05). Linear dose-response analyses showed a 10%, 12%, 9%, and 8% risk reduction in CVD, CHD, stroke, and AF incidence, respectively, for every 20 MET-hours/week increase in LTPA. In nonlinear dose-response analyses, there were inverse associations up to 20 MET-hours/week with 19% and 20% reduction in CVD and CHD risk, and up to 25 MET-hours/week with 22% reduction in stroke, with no further risk reduction at higher LTPA levels. For AF, there was a U-shaped nonlinear association with the maximum 8% risk reduction at 10 MET-hours/week of LTPA. Higher levels of OPA were not associated with risk of CVD, CHD, stroke, or AF.

**Conclusions:**

Overall, results showed an inverse dose-response relationship between LTPA and risk of CVD, CHD, stroke, and AF. Running was the most beneficial LTPA but the risk was similar among various LTPA intensities. OPA showed no benefits in total or any type of CVD.

**Supplementary Information:**

The online version contains supplementary material available at 10.1186/s12966-024-01593-8.

## Background

Cardiovascular diseases (CVD) are a spectrum of diseases related to the heart and circulation [1] and coronary heart disease (CHD) and stroke are still the leading causes of death worldwide [2]. The number of people living with CVD is rising, particularly in low and middle-income countries, while reductions in rates of CVDs have been observed in high income countries due to improvements in some cardiovascular risk factors and improved treatments. The number of prevalent CVD cases globally increased from 285 million in 1990 to 350 million in 2000, more than 430 million in 2010, and about 550 million in 2022 [1].

Lifestyle factors such as diet and physical activity play a major role in the development of CVD [3, 4]. Physical inactivity is estimated to be responsible for 7.6% of global CVD mortality [5]. In contrast, physical activity has shown benefits of reducing CVD morbidity and mortality in individuals with or without CVD [6]. This protection may be exerted through prevention of general and abdominal obesity, and improvement of cardiometabolic risk factors such as blood glucose, lipoproteins, and blood pressure [7].

Although the benefits of physical activity in cardiovascular system are well known, recent studies have found different effects by physical activity domains on CVD [8]. There are four main domains of physical activity: leisure, occupational, transport, and domestic or household [9]. The benefits of physical activity for improvement of health and prevention of diseases are mainly related to leisure-time physical activity (LTPA) [10]. However, for the occupational physical activity (OPA) the evidence is conflicting, and some studies have rejected the association of OPA and CVD or even found harmful effects [11].

A number of meta-analyses have examined the association of physical activity and CVD risk [12–17]. Most of these meta-analyses were published more than a decade ago. Recently, a dose-response meta-analysis of prospective studies examined the association between non-occupational physical activity and the risk of CVD mortality. However, it excluded studies that reported non-fatal CVD incidence and considered both leisure-time physical activity and domestic activities as non-occupational physical activity, while the current meta-analysis has a more distinct and specific look at LTPA. Additionally, none of the previous meta-analyses [12–17] performed a dose-response meta-analysis for the relationship between OPA and CVD incidence. Furthermore, it is unclear whether some types (such as walking, cycling, running, jogging, and stair climbing) or intensities of physical activity are more beneficial on CVD risk than others. To fill these gaps, we aimed to conduct a comprehensive meta-analysis on the association of each of LTPA and OPA and the incidence of major types of CVD (CHD, stroke, atrial fibrillation (AF), and overall CVD). In this meta-analysis, linear and nonlinear dose-response relationships were explored, the optimal volume of physical activity was estimated, and intensities and types of activity that provide the most prevention were determined.

## Methods

We followed the Preferred Reporting Items for Systematic Reviews and Meta-Analyses (PRISMA) [18] and Meta-analyses Of Observational Studies in Epidemiology (MOOSE) [19] for reporting meta-analyses. The protocol of this meta-analysis has been registered in the International Prospective Register of Systematic Reviews (PROSPERO; www.crd.york.ac.uk/prospero/index.asp; identifier CRD).

### Search strategy

We systematically searched three databases of PubMed, Scopus, and Web of Science from inception up to August 30, 2023. Details of the search strategies are presented in Supplementary Table 1.

### Eligibility criteria and study selection

Four authors (AK, ZM, EH, and ZH) reviewed the titles and abstracts of articles to select studies meeting the eligibility criteria. The eligible studies were all prospective cohort studies which had measured leisure-time/occupational physical activity as the exposure and any type of CVD incidence as the outcome (total CVD, CHD, stroke, and AF) in the general adult population aged ≥ 18 years. We excluded studies that reported on heart failure, since a recent meta-analysis was conducted on heart failure [20]. Studies that reported fatal events were also excluded, but they were included if both fatal and nonfatal events were reported in combination. Retrospective cohort and case-control studies, studies with a follow-up duration of ≤ two years, and studies conducted exclusively in populations with specific diseases or lifestyles (athletes) were also excluded. When more than one publication was published from the same cohort, only the most recent publication with the longest follow-up was included in high vs. low and linear dose-response meta-analyses. For the dose-response meta-analyses, the publications with the most complete information were used. If the risk was reported at two or more time points in a study, the data with the longest follow-up was used. If a study reported only total physical activity or did not clearly define the type of physical activity, we excluded it. Studies that reported having measured LTPA/recreational physical activity, or exercise/sports were included in the LTPA analysis. Studies reporting on LTPA combined with commuting physical activity were included but studies reporting on LTPA and activities during work at home were excluded. A list of studies that were excluded along with reasons of exclusion are provided in Supplementary Table 2.

### Data extraction and risk of bias assessment

Three authors (SS, ZM, EH) extracted the data from eligible studies, and one author (AK) checked for completeness, accuracy, and consistency. The extracted data include study characteristics (name of first author, year of publication, country, cohort name), participants characteristics (age, sex), sample size, number of CVDs cases, method of CVD and physical activity assessment, follow-up duration, adjustment factors, type, level, and intensity of physical activity, frequency of physical activity assessment, multivariable-adjusted risk estimate [risk ratios (RRs), hazard ratios (HRs), or odds ratios (ORs) with their corresponding 95% confidence intervals (CIs)]. When studies did not report sufficient information for the study to be included in the analyses, we contacted the authors by email at least two times. Any disagreement was resolved by consensus between two of the reviewers (AK, DA).

Risk of bias of studies was evaluated using the Risk of Bias In Non-randomised Studies of Interventions (ROBINS-I) [21]. This tool assesses the risk of bias based on seven items including, bias due to confounding, bias in selection of participants, bias in classification of exposures, bias due to deviations from intended exposures, bias due to missing data, bias in measurement of the outcome, and bias due to selective reporting of the results.

### Data synthesis and statistical analysis

For all analyses, HRs and 95% CIs were used as the effect sizes. The reported risk ratios and relative risks were considered as being equivalent to HRs. The random-effects model by DerSimonian and Laird was used to calculate summary HRs for the highest vs. lowest category of LTPA and OPA and per 20 metabolic equivalent (MET)-hours/week increase in LTPA in the dose-response analysis [22]. If the risk estimates were reported stratified by sex or other subgroups, but not overall, we pooled the subgroup-specific risk estimates using a fixed-effects model to generate an overall estimate before inclusion in the main analysis. To better control for residual confounding, we estimated the E value using the methodology proposed by Vanderweele and Ding [23]. We used the generalized least squares trend estimation method, by Greenland and colleagues for the linear dose-response analysis [24, 25]. For the non-linear dose response meta-analysis, we modelled the exposures by applying restricted cubic splines with three knots based on Harrell’s recommended percentiles (10%, 50%, and 90%) of the distribution. This method combines each study specific slope to obtain an overall average slope in a single stage [26]. The number of events and participants or person years, the levels of physical activity, and adjusted HRs with their 95% CIs in each category of exposure were requisite inputs when using this method. If the numbers of participants or person-years in each category were not presented in a study and the exposures were defined as quantiles, we divided the total number of participants or person years by the number of categories to estimate the missing distributions. If the exposures were not defined as quantiles, the numbers of cases and person-years in each category was estimated, using information on the total number of cases and the number of total participants plus the follow-up period as described previously [20]. The median MET-hours/week, kcal/week, and h/week per category was used to estimate the level of physical activity. We ascribed a dose of 45 min per session to studies that reported physical activity frequency per week or month [27]. For the moderate and vigorous exercise, we translated the data to MET-hours/week by multiplying the number of h/weeks by a factor of 4 and 8 [28]. For the open-ended categories, the width was considered equal to the adjacent category. When a study considered a category other than the lowest one as a reference, we recalculated the HRs and 95% CIs using the method by Hamling [29].

We conducted subgroup analyses according to potential important pre-specified factors, including sex, length of follow-up, geographical location, number of cases, and risk of bias (overall risk of bias and each component). We additionally conducted exploratory subgroup analyses by type of stroke and overall LTPA vs. sport. We explored publication bias using Egger’s asymmetry test and by inspection of the funnel plots. We conducted sensitivity analyses by excluding one study at a time and re-estimating the HRs to check whether a study with large sample size or a study with an extreme result impacted the summary estimates. Stata version 16 software was used to conduct all statistical analyses.

### Certainty of evidence assessment

The certainty of evidence was evaluated using the updated Grading of Recommendations, Assessment, Development and Evaluation (GRADE) approach which integrates the application of ROBINS-I [30]. GRADE tool rates the certainty of evidence as high, moderate, low, or very low. Observational studies start at a high certainty of evidence level in the updated GRADE. The evidence was downgraded based on the following criteria, risk of bias as assessed by ROBINS-I tool, inconsistency (substantial unexplained between-study heterogeneity, as represented by I^2^ ≥ 50% and inconsistency between the size and direction of risk estimates of the studies), indirectness (existence of population factors that limit the generalizability of the findings), imprecision [if 95%CIs of risk difference (RD) failed to exclude important harm (RD > 1.0) and benefit (RD < -1.0)], and evidence of publication bias. The certainty of evidence was upgrading using the following criteria: existence of a dose-response gradient and large effect size. Large size was defined as RR > 2.0 or < 0.5, at any specific dose of the exposure in the non-linear dose-response meta-analyses [31].

## Results

### Study screening

**Fig. 1:**
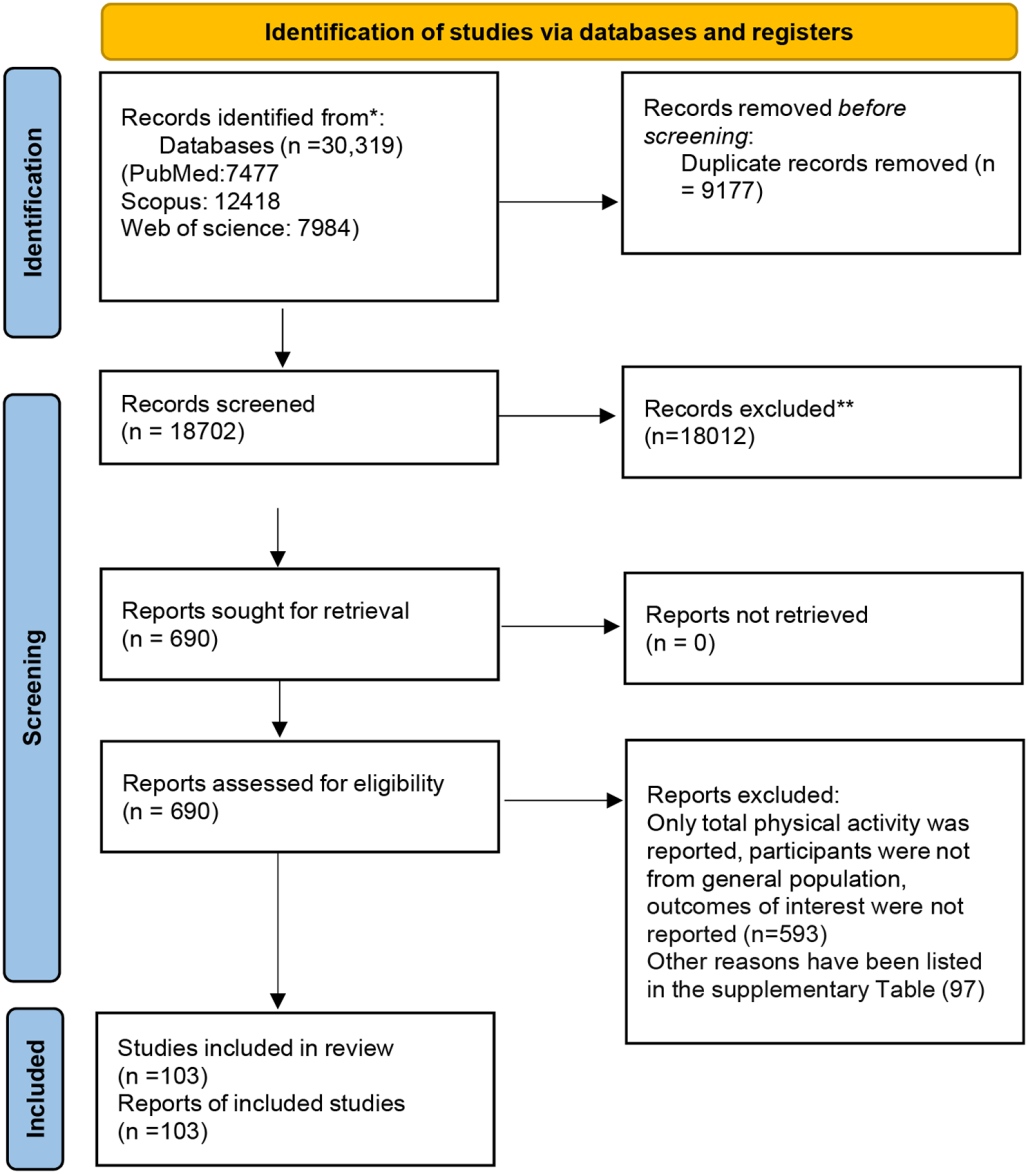
Flow diagram of studies selection process

A total of 30,319 papers (including 8,078 from PubMed, 13,624 from Scopus, and 8,617 from Web of Science) were originally retrieved. After removing duplicate records and screening titles and abstracts, full texts of 710 citations were evaluated and finally 103 studies were included in the analysis [32–128]. The flow diagram of the meta-analysis is shown in Fig. 1. The reasons for excluding studies other than those reported in the flowchart are provided in Supplementary Table 2. Of the included studies, 95 publications provided information on LTPA/recreational activity (*n* = 89) and sports (*n* = 8), and 24 citations reported OPA. Overall, 40 studies reported data on the association between LTPA and CVD risk (2,876,417 participants and 290,811 cases), 38 on CHD risk (2,567,921 participants and 115,389 cases), 30 on stroke risk (2,568,711 participants and 76,170 cases), and 12 on AF risk (764,640 participants and 24,642 cases). Regarding OPA, seven studies (733,300 participants and 46,543 cases) yielded findings on CVD risk; 12 studies on CHD (630,236 participants and 14,122 cases); six studies on stroke (625,347 participants and 37,342 cases), and two studies on AF (53,708 participants and 5,035 cases). Co-published articles from the same cohort studies are listed in Supplementary Table 3.

For dose-response meta-analysis of LTPA, 38 studies did not report the required data (Supplementary Table 4). Among the remaining studies, 65 reported physical activity in MET-hours/week, five in kcal/week, and five in hours/week. Because the studies that reported on OPA did not report data in a format that could be used for dose-response analyses, we were unable to conduct dose-response analysis for OPA.

### Study characteristics

All studies had a prospective cohort design. The mean age of cohort participants at baseline ranged from 28.8 to 85.9 years. The majority of studies (*n* = 63) came from Europe, 28 from the US, eight from Asia, three from Australia, and one international study from 17 high, medium, and low-income countries. Of the 103 studies, 24 included males, 17 included females, and 62 reported results for both sexes (13 articles described stratified analyses by sex). Two-thirds of the studies (*n* = 75) involved the general population; ten studies involved middle-aged subjects; nine studies involved elderly subjects; six studies involved workers; six studies involved physicians and nurses; two studies involved postmenopausal women; one study involved obese adults; and one study involved a twin population. The follow-up duration was more than 13 years in 44 studies. The outcomes of interest were determined in 80% of the studies (*n* = 82) by records of hospital discharge diagnosis, medical reports, and national patient registry linkages, while the outcomes were identified in five studies through actual assessment (physician diagnosis), in seven studies through self-report confirmed by physician diagnosis, seven studies based on self-reported data, one study did not mention the assessment method, and one used mixed methods. More than a half of the studies yielded information on physical activity through validated questionnaires (*n* = 59). A total of 74 studies measured physical activity only at baseline but others performed multiple assessments throughout the cohort course. All the studies adjusted for sex or were conducted only in males or females, all except three adjusted for age, approximately 70% adjusted for body mass index (BMI), 78% adjusted for cigarette smoking and alcohol, 52% adjusted for dyslipidemia or lipid-lowering medications, and more than two-thirds adjusted for blood pressure and anti-hypertensive drugs, and a quarter of the studies adjusted for dietary patterns or food items. Supplementary Tables 5–12 contain descriptive data for the included studies according to the outcomes. As most of the studies did not adjust for diet, in the subgroup analysis by risk of bias, the adjustment for diet was ignored if only one or two studies were in the moderate subgroup.

### Risk of bias assessment

Seven studies had moderate, and the remaining had serious risk of bias. Only 16 studies adequately adjusted for confounding variables (age, sex, BMI, alcohol, smoking, and diet) and other studies did not fully adjust for potential confounders. Nearly 37% of studies were susceptible to selection bias because of conducted in specific population like elderly, workers, physicians, or twins. Thirty-nine studies had serious risk for exposure assessment bias because physical activity was measured by a questionnaire that had not been validated or a single question. Twenty-four studies had low risk of misclassification bias since physical activity was assessed repeatedly during the follow-up. 82% of the studies had low risk of missing data due to adequate descriptions of the loss to follow-up. The majority of studies (*n* = 90) obtained data through medical/hospital reports and national patient registries and thus had low risk of bias for measurement of outcomes. None of studies were biased by selective data reporting. A summary of the risk of bias assessment is provided in Supplementary Table 13.

## Meta-analysis

### LTPA and the risk of CVD

#### High vs. low analysis

**Fig. 2:**
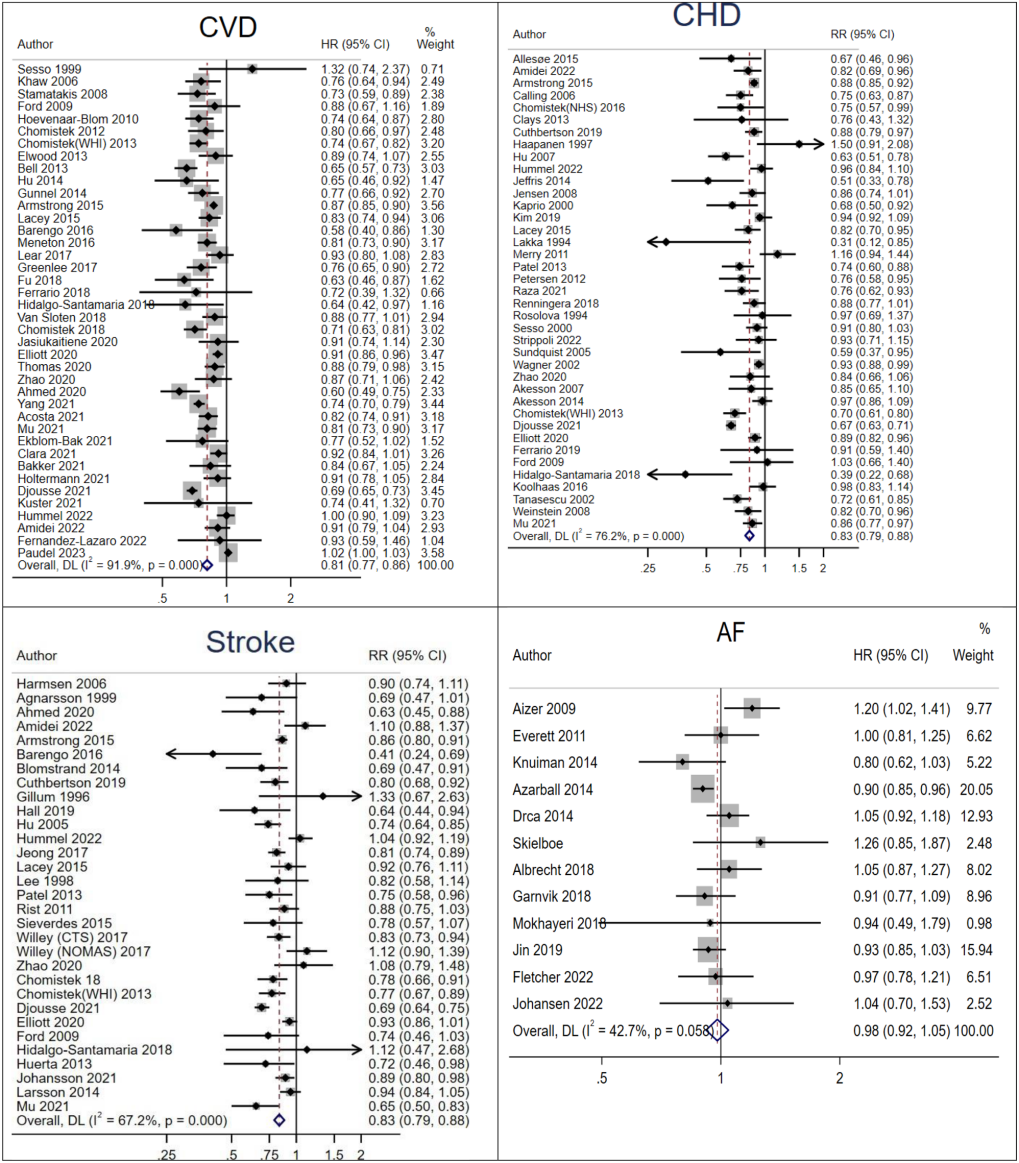
Summary HR of cardiovascular disease (CVD), coronary heart disease (CHD), stroke, and atrial fibrillation (AF) incidence for the highest compared with the lowest category of leisure time physical activity

Forty cohort studies (2,876,417 participants, 290,811 cases) investigated the association between LTPA and the risk of CVD [32, 34, 40, 41, 43–45, 50, 51, 53, 54, 57, 60–62, 64–66, 69, 70, 74, 75, 77, 80, 82, 83, 91, 96, 97, 100, 102, 105, 114, 116, 120, 121, 126, 127]. The summary HR for the highest vs. the lowest categories of LTPA was 0.81 (95% CI: 0.77 to 0.86, I^2^ = 91.9%; RD: 1.92 fewer CVD cases per 100 participants, 95% CI: 2.32 fewer, 1.41 fewer; GRADE = moderate) (Fig. 2). Sequential removal of studies did not change the direction or magnitude of the pooled HR (HR range = 0.80–0.82). The estimated E value for point estimate was 1.58 with a lower confidence CI of 1.51. There was no heterogeneity between subgroups, except for a stronger association among studies that reported on sports, running, and jogging than among studies reporting on other types of LTPA (Supplementary Table 14). Inspection of funnel plot (Supplementary Fig. 1) and Egger’s test for asymmetry (*P* < 0.001) showed an indication of small study effects. However, no study was included in trim and fill analysis.

#### Dose-response analysis

Nineteen studies were included in the linear dose-response meta-analysis [44, 50, 53, 56, 61, 64, 70, 74, 97, 120, 121, 128]. The estimated risk reduction for CVD was 10% (HR = 0.90; 95% CI: 0.87 to 0.93; *I*^2^ = 88.6%, P_heterogeneity_<0.001) per 20 MET-hours/week increment of LTPA (Supplementary Fig. 2).

**Fig. 3:**
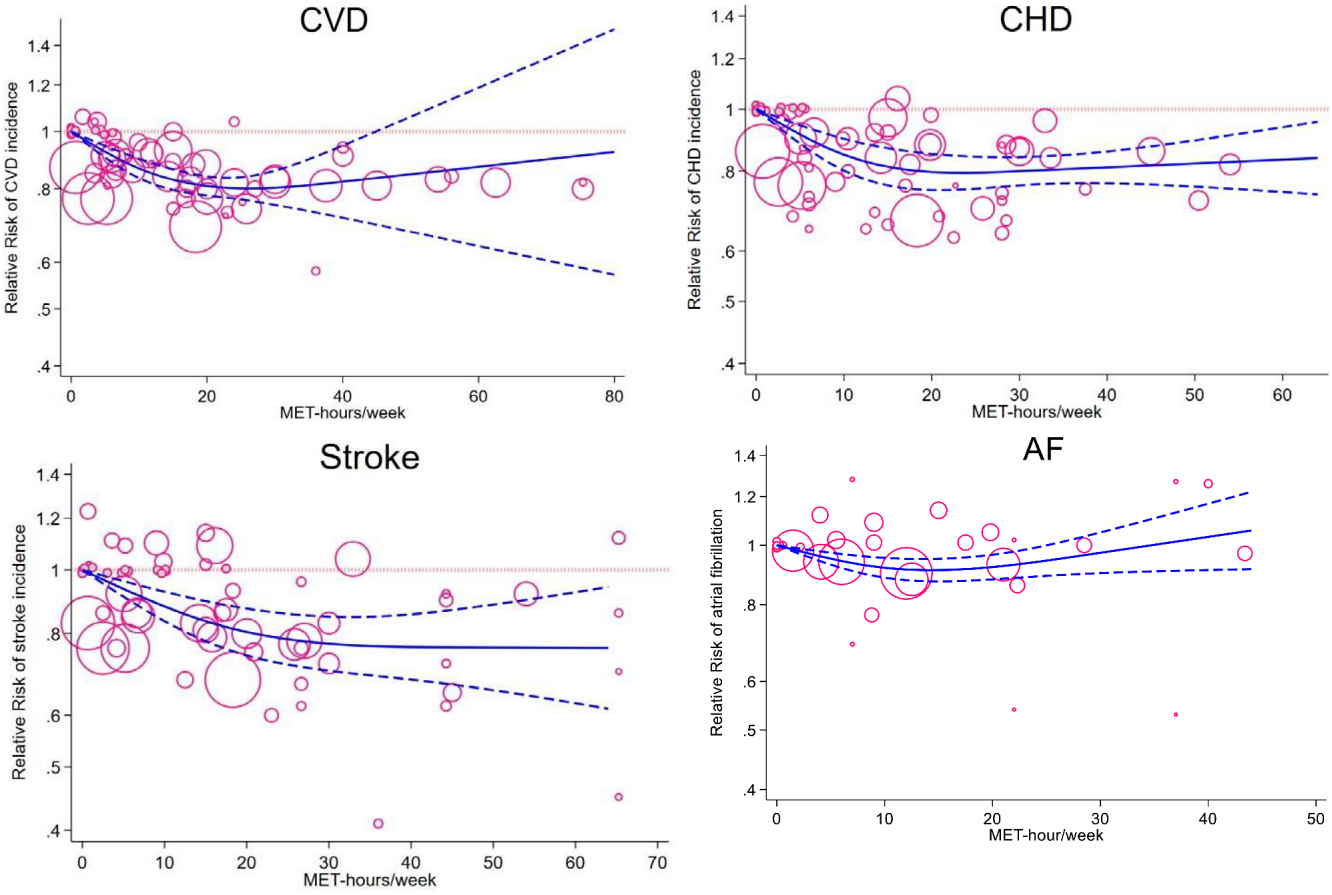
Dose-response relationship between leisure time physical activity (LTPA) and cardiovascular disease (CVD), coronary heart disease (CHD), stroke, and atrial fibrillation (AF). The solid line represents non-linear dose response and dotted lines represent 95% confidence interval. Circles represent HR point estimates for LTPA categories from each study with circle size proportional to inverse of standard error. Small grey circles represent baseline LTPA category for each separate study

Twenty-three studies were included in the non-linear dose-response analysis (17 reported physical activity in MET-hours/week [32, 40, 43, 44, 50, 53, 56, 57, 60, 61, 64, 70, 77, 97, 104, 121, 128], 2 in kcal/week [51, 114], and 4 in hours/week [41, 75, 91, 116]). In the non-linear dose-response analysis, a dose-dependent reduction in the risk of CVD incidence was observed up to 19% at 20 MET-hours/week, with little or no further decrease in risk at higher levels (P_nonlinearity_<0.001) (Fig. 3). Excluding studies with two categories (*n* = 2) [40, 70] did not change the results (Supplementary Fig. 3). For kcal/week and h/week, the largest reductions in risk was 25% and 16% which were observed at LTPA of 1300 kcal/week and 3 h/week, respectively, with no further reduction in risk observed at higher levels (Supplementary Figs. 4 and 5).

### LTPA and the risk of CHD

#### High vs. low analysis

Thirty-nine cohort studies (2,594,495 participants, 120,048 cases) investigated the association between LTPA and risk of CHD [36, 37, 39–41, 49, 52, 53, 55–57, 61, 64, 65, 71, 74, 79, 82, 84, 85, 90, 92, 94, 97, 98, 103, 105–109, 111, 113, 117–119, 122, 123, 127]. Compared with the lowest LTPA, the highest LTPA was associated with a lower risk of CHD (HR = 0.83; 95% CI: 0.79 to 0.88, *I*^*2*^ = 76.2%; RD: 0.78 fewer per 100 participants, 95% CI: 0.97 fewer, 0.60 fewer; GRADE = moderate) (Fig. 2). The summary estimate remained unchanged after the sequential omission of each study from the main analysis (HR range = 0.82–0.84). The estimated E value for point estimate was 1.53 with a lower CI of 1.41. The association was stronger among studies reported running and walking than other types of physical activity. There was no heterogeneity between subgroups for other factors (Supplementary Table 15). There was no clear evidence of publication bias with the Egger’s test or by inspection of the funnel plot (*P* = 0.28) (Supplementary Fig. 6).

#### Dose-response analysis

Eighteen studies were included in the linear dose-response meta-analysis [40, 46, 52, 53, 56, 57, 61, 64, 82, 85, 94, 97, 105–107, 119, 122, 128]. For each 20 MET-hours/week increment of LTPA, the summary HR of CHD was 0.88 (95% CI: 0.84 to 0.92, *I*^2^ = 88.1%, *P* < 0.001 for heterogeneity) (Supplementary Fig. 7).

Twenty-one studies were included in the non-linear dose-response analysis [MET-hours/week (*n* = 15) [40, 52, 53, 56, 57, 61, 64, 82, 85, 94, 97, 105–107, 119], kcal/week (*n* = 3) [51, 71, 113], and hours/week (*n* = 3) [41, 92, 108]]. A dose-dependent reduction in risk was observed up to 20 MET-hours/week where a 20% reduction in risk was observed, and there was little or no further decrease in risk at higher levels (P_nonlinearity_=0.001) (Fig. 3). Excluding the one study with two categories did not change the results (Supplementary Fig. 3) [40]. For the two other analyses of LTPA, the lowest risk was observed at LTPA of 1100 kcal/week and 2 h/week with 36% and 18% reductions in risk, respectively, and again there was no further reduction in risk at higher levels (Supplementary Figs. 8 and 9).

### LTPA and the risk of stroke

#### High vs. low analysis

Thirty-one cohort studies (2,595,295 participants, 77,215 cases) investigated the association between LTPA and risk of stroke [33, 34, 40, 41, 44, 47, 51, 53, 56, 57, 61, 65, 68, 72–74, 78, 81, 82, 86, 88, 97, 99, 101, 105, 106, 110, 112, 124, 125, 127]. The highest compared to the lowest level of LTPA was associated with a lower stroke risk (summary HR = 0.83; 95% CI: 0.79 to 0.88; *I*^*2*^ = 67.2%; RD: 0.48 fewer per 100 participants, 95%CI: 0.63 fewer, 0.33 fewer; GRADE = moderate) (Fig. 2). The summary estimate remained unchanged after sequential omission of each study from the main analysis (HR range = 0.84–0.85). The estimated E value for point estimate was 1.53 with a lower CI of 1.41. The association was slightly stronger among studies that used self-reported outcome assessment compared to medical record or doctor diagnosed outcome assessment and among studies that did not adjust for diabetes (Supplementary Table 16). The risk reduction appeared to be more evident in transient ischemic attack (HR = 0.69; 95% CI: 0.56 to 0.84; *I*^2^ = 0.0%) vs. ischemic and hemorrhagic types of stroke. No publication bias was detected with Egger test or by inspection of the funnel plot (*P* = 0.59) (Supplementary Fig. 10).

#### Dose-response analysis

Thirteen studies were included in the linear dose-response analysis of LTPA and stroke [40, 53, 56, 57, 61, 81, 82, 86, 97, 105, 106]. For each 20 MET-hours/week increment of LTPA, the pooled risk was reduced by 9% (summary HR = 0.91; 95% CI: 0.88 to 0.94; *I*^2^ = 59.5%) (Supplementary Fig. 11).

Sixteen studies were included in the non-linear dose-response analyses [MET-hours/week (*n* = 12) [40, 44, 53, 56, 57, 61, 81, 82, 86, 97, 105, 106], kcal/week (*n* = 2) [51, 101], and h/week (*n* = 2) [41, 92]]. A dose-dependent reduction in risk was observed up to 25 MET-hours/week where a 22% reduction in risk was observed, and there was no further reduction in risk at higher levels (P_nonlinearity_=0.06) (Fig. 3). Excluding one study with two categories did not change the results (Supplementary Fig. 3) [40]. For studies that reported kcal/week and hours/week, the lowest risk was observed at LTPA of 2500 kcal/week and 2 h/week and with a 34% and 22% reduction, respectively, and again there was no further reductions in risk at higher levels (Supplementary Figs. 12 and 13).

### LTPA and the risk of AF

#### High vs. low analysis

Twelve cohort studies (764,640 participants, 24,642 cases) investigated the association between LTPA and risk of AF [35, 38, 42, 58, 63, 67, 87, 93, 104, 115]. Comparing the highest to the lowest categories of LTPA, no association between LTPA and risk of AF was found (summary HR = 0.98; 95% CI: 0.92 to 1.05; *I*^2^ = 42.7%; RD: 0.06 fewer per 100 participants, 95%CI: 0.26 fewer, 0.16 more; GRADE = moderate) (Supplementary Table 17, Fig. 2). The non-significant association persisted across all subgroups and there was no between subgroup heterogeneity in the subgroup analyses, except for a stronger association among females (Supplementary Table 17). The summary estimate did not materially change when one study was excluded at a time (HR range = 0.94-1.00). The estimated E value for point estimate was 1.13 with a lower CI of 1.00. No publication bias was observed with the Egger’s test and or by inspection of the funnel plot (*P* = 0.15) (Supplementary Fig. 14).

#### Dose-response analysis

Six studies were included in the linear dose-response analysis [38, 42, 63, 87, 104, 115]. For each 20 MET hours/week increment in LTPA, the risk of AF was reduced by 8% (summary HR = 0.92; 95% CI: 0.85 to 0.99; *I*^2^ = 37.5%; P_heterogeneity_=0.16) (Supplementary Fig. 15). Nine studies were included in the non-linear dose-response analysis [MET-hours/week (*n* = 7) [38, 42, 63, 87, 104, 115] and h/week (*n* = 2) [35, 58]]. A U-shaped association with an 8% reduction in the risk of AF incidence was observed at 10 MET-hours/week, but the curve moved closer to the null at higher levels of activity (P_nonlinearity_<0.001) (Fig. 3). Analysis of studies that reported hours/week indicated no evidence of a non-linear association (Supplementary Fig. 16).

#### Association of the OPA with the risk of CVD, CHD, AF, and stroke

Seven studies with 733,300 participants and 46,543 cases were included in high vs. low meta-analysis for CVD [43, 46, 64, 77, 80, 91, 96], 12 studies with 630,236 participants and 14,122 cases for CHD [39, 46, 55, 64, 76, 79, 89, 95, 103, 107, 117], six studies with 625,347 participants and 37,238 cases for stroke [46, 48, 72, 78, 81], and two studies with 53,708 participants and 5,035 cases for AF [58, 115]. Comparing the highest with the lowest categories of OPA, no association was observed between OPA and risk of CVD (HR = 1.01; 95% CI: 0.77 to 1.32; *I*^*2*^ = 88.4%, P_heterogeneity_<0.001), CHD (HR = 0.90; 95% CI: 0.78 to 1.04; *I*^*2*^ = 87.5%, P_heterogeneity_<0.001), stroke (HR = 0.91; 95% CI: 0.80 to 1.04; *I*^*2*^ = 80.2%, P_heterogeneity_<0.001) and AF (HR = 1.17; 95% CI: 0.99 to 1.38; *I*^*2*^ = 37.8%, P_heterogeneity_=0.20) (Supplementary Figs. 17–20). The summary estimates remained unchanged after sequentially excluding each study for all four outcomes. The results of the subgroup analyses for CVD, CHD, and stroke are presented in Supplementary Tables 18–20. There was no indication of publication bias in the analysis of CHD statistically or visually (*P* = 0.83) (Supplementary Fig. 21). The heterogeneity of the data did not allow for dose–response analyses.

#### Certainty of evidence

The overall certainty of evidence is presented in Supplementary Tables 21 and 22. The certainty of evidence for the association between LTPA and risk of AF was rated “high”, whereas the certainty of evidence for the association between LTPA and CVD, CHD, and stroke were rated as “moderate”. The evidence was graded as “very low” for the association between OPA and risk of CVD, CHD, stroke, and AF.

## Discussion

### Principal findings

The results of this meta-analysis showed the highest vs. the lowest LTPA was associated with a 19%, 17%, and 17% lower risk of overall CVD, CHD, and stroke, respectively. Linear dose-response analyses showed a 10%, 12%, 9%, and 8% risk reduction in CVD, CHD, stroke, and AF incidence per 20 MET-hours/week increase in LTPA. The estimated E value for point estimate was 1.58 (lower CI: 1.51), 1.53 (lower CI: 1.41), and 1.53 (lower CI: 1.41) for the incidence of CVD, CHD, and stroke, respectively. These E values suggest unmeasured confounders should have this size of the association with both the exposure and the outcome to completely explain away the observed association.

In nonlinear dose-response analyses, there were dose-dependent inverse associations up to 20 MET-hours/week with 19% and 20% reduction in CVD and CHD risk, respectively, and up to 25 MET-hours/week with a 22% reduction in the risk of stroke, with no further reduction at higher LTPA levels. For AF, there was a U-shaped nonlinear association and the maximum reduction in the risk was 8% at around 10 MET-hours/week of LTPA, with going toward null association at higher LTPA levels. For hours/week measurements, the largest risk reduction for CVD was 16% at 3 h/week LTPA, and for CHD and stroke was 18% and 22%, respectively, at 2 h/week LTPA. Higher levels of OPA were not associated with lower risk of total CVD, CHD, stroke and AF.

Subgroup analyses based on risk of bias, location, follow-up duration, adjustment variables, and number of CVD incidence were in general consistent with the main analyses in the direction and magnitude. However, subgroup analysis based on sex showed different results between males and females for AF, where only females demonstrated protective effect of physical activity.

Associations were relatively similar between different intensities of physical activity, suggesting that physical activity in any intensity could be beneficial. Running appeared to be associated with a stronger reduction in the risk compared to other types of physical activity, although considering the limited data available, further studies are needed.

### Comparison with previous meta-analyses

This was an updated meta-analysis based on results of 103 prospective cohort studies with follow-up durations ranging from 3 to 44 years (74 studies > 10 years). The findings were in line with previous meta-analyses that found an inverse association between physical activity and CHD, stroke, and CVD morbidity/mortality [12–17] but were contrary to the positive association observed between OPA and CVD risk a decade ago based on reports of 23 prospective cohort studies [14]. The extent of risk reduction was almost comparable between results of this meta-analysis and a recent meta-analysis which found a curvilinear association between non-occupational physical activity levels and the incidence of CHD, heart failure and stroke [17].

### Mechanisms for the benefits of physical activity on cardiovascular system

CHD, stroke, and AF are types of CVD [129]. Atherosclerotic plaques are the primary core of these diseases which are formed and triggered upon exposure to risk factors such as high blood pressure, high low-density lipoprotein, obesity, smoking, unhealthy diet, sedentary lifestyle, and physical inactivity. Many of these risk factors are controlled by prevention of obesity and correcting lifestyle especially that of physical activity. Adherence to the recommendations of the guidelines for physical activity prevents accumulation of excess fat mass particularly around the abdomen and visceral areas, thus preventing development of cardiometabolic risk factors [130, 131]. Besides obesity prevention, aerobic physical activity improves endothelial function and prevents arterial stiffness [132]. LTPA is also negatively associated with oxidative stress and inflammatory markers which are important underlying factors in the process of atherosclerosis [133, 134]. Also, blood coagulation is diminished with regular physical activity, likely due to lower levels of coagulation factors such as blood fibrinogen and tissue plasminogen activator [135]. Parts of these benefits are exerted independently of the effect of physical activity on weight control. This has been documented in previous investigations [136–139] and also in results of this meta-analysis where subgroup analyses based on adjustment for BMI did not reveal a difference in the results.

### Physical activity and AF

The extent of LTPA benefits on AF was lower than that for other outcomes (i.e. CVD, CHD, and stroke): the risk reduction was 22.6% lower for linear and 60.7% lower for non-linear dose-response relationships. This low level of protection was only observed in females. This finding is in line with previous meta-analyses that found the inverse relationship between physical activity and AF risk only in females [140–142]. It has been known that the risk of AF in females may reduce with moderate to vigorous intensity physical activity, but in males, moderate intensity physical activity is beneficial while vigorous physical activity may increase AF risk [142, 143]. The reason of the opposite effect of physical activity on AF risk in males and females is not clear at this time. However, a study on athletes revealed that under identical training hours and race time, male athletes had more noticeable atrial remodeling, a concentric type of ventricular remodeling, blood pressure at rest and during exercise, and a sympathetic tone than female athletes [144]. Females also have lower amounts and intensity of physical activity, fewer heart comorbidities, and lower sympathetic tone, and lower blood pressure than males [145].

AF also showed a U-shaped nonlinear relationship with LTPA. Such a relationship has also been reported in previous meta-analyses [141, 146]. The high-intensity physical activity may increase volume load and promote atrial enlargement, remodeling, and fibrosis, increase vagal tone, and inflammation, while low/moderate and particularly regular physical activity may protect against AF through prevention of cardiometabolic risk factors, regulation of autonomic system, and improved cardiac structure and function.

### Occupational physical activity

OPA did not show a relationship with CVD, CHD, stroke, and AF incidence in this meta-analysis. Previous meta-analyses have produced contradictory results for this kind of relationship: Li et al. in a meta-analysis performed on prospective cohort studies published in the time course between 2011 and 2013, found a significant positive association between high levels of OPA and CHD as well as overall CVD but not with stroke and unclassified CVD or between moderate intensity OPA and any of CHD, stroke, and unclassified CVD risk [14]. In contrast, Wendel et al. in a meta-analysis published in 2004 reported protection by moderate and especially high intensity OPA against stroke [147]. The reason of discrepancies is not clear. The present meta-analysis was performed on more recent longitudinal studies with probably better methodological design and more appropriate analysis compared to studies conducted 10–20 years ago. For instance, in recent works, the control of confounders has been performed with more scrutiny and precision than before. Although non-significant, OPA showed a trend for reverse association with CHD (*n* = 12) and stroke (*n* = 6), and a trend for positive association with AF (*n* = 2). Future longitudinal studies are required to determine these relationships with greater certainty.

### Recommended levels of physical activity

According to the physical activity guidelines for Americans, adults should do at least 150 to 300 min a week (2.5 to 5 h/week) of moderate-intensity, or 75 to 150 min a week of vigorous-intensity aerobic physical activity [148]. These amounts are comparable to the 20 MET-hours/week found to be associated with 8–12% risk reduction in the outcomes of this study. Twenty MET-hours/week is roughly equivalent to 3.5 to 5 h/week moderate-intensity physical activity (3.5 h/week for activities such as brisk walking and slow jogging which have a MET value of 6, and 5 h/week for activities like moderate-intensity walking with a MET value of 4) [149]. Higher levels of physical activity may deliver additional benefits [16]. An individual participant meta-analysis of prospective cohort studies showed 60–75 min/day moderate-intensity physical activity might eliminate the increased risk of death associated with high sitting time [150].

Unfortunately, the cohort studies did not generally measure the intensity of physical activity. Measurement units of MET-hours/week, kcal/week, and hours/week are in fact indicative of either combined intensity and duration (MET-hours/week and kcal/week) or duration only (hours/week). According to the available evidence, it seems that the duration of physical activity has a quite important impact on the reduction of CVD risk [151] but the intensity of exercise should be adapted to the cardiorespiratory capacity and medical conditions of the individual [134]. A meta-analysis of 5 cohort studies that measured the intensity of physical activity by an accelerometer in older adults found that HR for CVD risk was lower in moderate-to-vigorous physical activity than light-intensity physical activity although HR for CVD death was almost equal in light- and moderate-to-vigorous intensity physical activity [152], suggesting that moderate-to-vigorous intensity physical activity may be more beneficial for CVD incidence than light-intensity activity.

### Strengths and limitations

Some important limitations should be considered in interpreting the results. First, only a few studies adjusted for diet or other types of physical activity; therefore, potential confounding factors should be considered. However, studies that controlled for these confounders showed similar results to those that did not, suggesting this may be less of an issue. Moreover, estimated E values showed that little unmeasured confounding would be needed to explain away the observed associations. Secondly, in most of the studies the potential changes in the level and type of physical activity during the follow-up period were not considered in the analyses. Additionally, physical activity was self-reported, which is not an accurate measurement of physical activity, and none of the included studies corrected for measurement errors. Moreover, relatively few studies provided information on the intensities of LTPA. Lastly, due to differences in how LTPA was measured and reported, only around half of the studies that were included in the high vs. low analysis could be included in the linear and nonlinear dose-response analyses. However, given the general consistency of the results across different analyses, and the large number of studies included in the dose-response analyses this should be less of an issue. The strengths of our study included the prospective design of the included studies. Furthermore, the large sample size for LTPA analysis, which included up to 306,694 cases and ∼ 5.3 million participants, provided sufficient statistical power to detect even modest associations. Moreover, the certainty of evidence for LTPA was moderate to high. Finally, examining nonlinear relationships clarified the shape of the dose–response relationships, suggesting that most of the benefit is observed at up to 20–25 MET-hours/week of activity.

## Conclusions and future implications

Overall, results of this meta-analysis showed an inverse dose-response relationship between LTPA and risk of CVD, CHD, stroke, and to a lesser extent AF. The dose-response relationship was most pronounced up to 20–25 MET-hours/week LTPA (equals to 3.5 to 5 h/week of moderate-intensity physical activity) for CVD, CHD, and stroke, and about half of that for AF, but no further reductions were observed with higher levels of LTPA. OPA showed no statistically significant association with total and type of CVD outcomes. Any further studies could benefit from more in-depth assessment of different types and intensities of physical activity as well as incorporating more objective measures of activity.

### Electronic supplementary material

Below is the link to the electronic supplementary material.

Supplementary Material 1

Supplementary Material 2

## Data Availability

The datasets used for the current study are available from the corresponding author on reasonable request.

## Abbreviations

AF: Atrial Fibrillation
BMI: Body Mass Index
CHD: Coronary Heart Disease
CI: Confidence Interval
CVD: Cardiovascular Disease
HR: Hazard Ratio
IHD: Ischemic Heart Disease
LTPA: Leisure-Time Physical Activity
OPA: Occupational Physical Activity
RD: Risk Difference
RR: Risk Ratio
