# Supplementary material for: Leisure-time and occupational physical activity and risk of cardiovascular disease incidence: a systematic-review and dose-response meta-analysis of prospective cohort studies
Source: Int J Behav Nutr Phys Act. 2024 Apr 24;21:45. doi: 10.1186/s12966-024-01593-8 (PMC11044601; doi:10.1186/s12966-024-01593-8)
Supplement: Supplementary file 1 — Supplementary Material 1 [file 12966_2024_1593_MOESM1_ESM.docx]

**Leisure-time and occupational physical activity and cardiovascular disease incidence: a systematic review and dose-response meta-analysis of prospective cohort studies**

**Supplementary Table 1. Search strategy**

| Pubmed: 8,078  (sedentar*[Title/Abstract]) OR (sedentar*[MeSH Terms])) OR (inactiv*[MeSH Terms])) OR (inactive*[Title/Abstract])) OR ("physical activity"[Title/Abstract])) OR ("physical activity"[MeSH Terms])) OR (exercise[MeSH Terms])) OR (exercise[Title/Abstract])) OR (walking[Title/Abstract])) OR (walking[MeSH Terms])) OR (sport[MeSH Terms])) OR (sport[Title/Abstract])) OR ("physical exertion"[Title/Abstract])) OR ("physical exertion"[MeSH Terms])) OR ("physical intensity"[MeSH Terms])) OR ("physical intensity"[Title/Abstract])) OR ("recreational activity"[Title/Abstract])) OR ("recreational activity"[MeSH Terms])) OR ("household activity"[MeSH Terms])) OR ("household activity"[Title/Abstract])) OR ("occupational activity"[Title/Abstract])) OR ("occupational activity"[MeSH Terms])) OR ("metabolic equivalent"[MeSH Terms])) OR ("metabolic equivalent"[Title/Abstract])) OR ("vigorous physical activity"[Title/Abstract])) OR ("vigorous physical activity"[MeSH Terms]))  AND  ("cardiovascular disease"[Title/Abstract]) OR ("cardiovascular disease"[MeSH Terms])) OR ("coronary artery disease"[MeSH Terms])) OR ("coronary artery disease"[Title/Abstract])) OR ("heart disease"[Title/Abstract])) OR ("heart disease"[MeSH Terms])) OR ("myocardial infarction"[MeSH Terms])) OR ("myocardial infarction"[Title/Abstract])) OR (stroke[Title/Abstract])) OR (stroke[MeSH Terms])) OR ("carotid artery disease"[MeSH Terms])) OR ("carotid artery disease"[Title/Abstract])) OR ("heart failure"[Title/Abstract])) OR ("heart failure"[MeSH Terms])) OR ("heart attack"[MeSH Terms])) OR ("heart attack"[Title/Abstract])) OR (Atherosclerosis[Title/Abstract])) OR (Atherosclerosis[MeSH Terms])) OR ("cerebrovascular disease"[MeSH Terms])) OR ("cerebrovascular disease"[Title/Abstract])) OR ("Peripheral vascular disease"[Title/Abstract])) OR ("Peripheral vascular disease"[MeSH Terms]))))  AND  (follow-up) OR ("relative risk")) OR ("hazard ratio")) OR ("odds ratio")))  AND  ((prospective*) OR (longitudinal)) OR (observation)) OR (observational)) OR (cohort*)) |
| --- |
| Scopus: 13,624  (TITLE-ABS-KEY(sedentar*) OR TITLE-ABS-KEY(inactive*) OR TITLE-ABS-KEY("physical activity") OR TITLE-ABS-KEY(exercise) OR TITLE-ABS-KEY(walking) OR TITLE-ABS-KEY(sport) OR TITLE-ABS-KEY("physical exertion") OR TITLE-ABS-KEY("physical intensity") OR TITLE-ABS-KEY("recreational activity") OR TITLE-ABS-KEY("household activity") OR TITLE-ABS-KEY("occupational activity") OR TITLE-ABS-KEY("metabolic equivalent") OR TITLE-ABS-KEY("vigorous physical activity"))  AND  (TITLE-ABS-KEY("cardiovascular disease") OR TITLE-ABS-KEY("coronary artery disease") OR TITLE-ABS-KEY("heart disease") OR TITLE-ABS-KEY("myocardial infarction") OR TITLE-ABS-KEY(stroke) OR TITLE-ABS-KEY("carotid artery disease") OR TITLE-ABS-KEY("heart failure") OR TITLE-ABS-KEY("heart attack") OR TITLE-ABS-KEY(Atherosclerosis) OR TITLE-ABS-KEY("cerebrovascular disease") OR TITLE-ABS-KEY("Peripheral vascular disease"))  AND  (TITLE-ABS-KEY (prospective) OR TITLE-ABS-KEY (prospectively) OR TITLE-ABS-KEY (observation) OR TITLE-ABS-KEY(observational) OR TITLE-ABS-KEY(cohort) OR TITLE-ABS-KEY(cohorts))  AND  (TITLE-ABS-KEY (follow-up)OR TITLE-ABS-KEY ("relative risk") OR TITLE-ABS-KEY("hazard ratio") OR TITLE-ABS-KEY("odds ratio") OR TITLE-ABS-KEY(duration)) |
| WOS: 8,617  (TS=(sedentar*) OR TS=(inactive*) OR TS=("physical activity") OR TS=(exercise) OR TS=(walking) OR TS=(sport) OR TS=("physical exertion") OR TS=("physical intensity") OR TS=("recreational activity") OR TS=("household activity") OR TS=("occupational activity") OR TS=("metabolic equivalent") OR TS=("vigorous physical activity"))  AND  (TS=("cardiovascular disease") OR TS=("coronary artery disease") OR TS=("heart disease") OR TS=("myocardial infarction") OR TS=(stroke) OR TS=("carotid artery disease") OR TS=("heart failure") OR TS=("heart attack") OR TS=(Atherosclerosis) OR TS=("cerebrovascular disease") OR TS=("Peripheral vascular disease"))  AND  (TS=(prospective) OR TS=(prospectively) OR TS=(observation) OR TS=(observational) OR TS=(cohort) OR TS=(cohorts))  AND  (TS=(follow-up) OR TS= ("relative risk") OR TS=("hazard ratio") OR TS=("odds ratio") OR TS=(duration)) |

**Supplementary Table 2. Reason for excluding of studies**

| **Design:**  Five studies were excluded since the design was not prospective cohort: Retrospective design: (1) case cohort (2), case control (3), case cohort(4, 5) |
| --- |
| **Outcome:**  The outcome of our interest (MI) was mixed with unrelated outcome (coronary artery bypass graft, percutaneous transluminal coronary angioplasty) and the result was not reported separately for each outcome (6)  Sarajilic (aortic valve stenosis)(7)  López-Laguna 2018 and Grenon 2014 assessed peripheral artery disease (8, 9)  The outcome was aortic stiffness (10)  The outcome was biomarkers of heart failure(11)  The outcome was Framingham Risk Scores of 15-year mortality due to CHD and CVD(12)  The outcome is the CVD risk score (13)  The outcome was coronary artery calcification (14, 15)  The outcome was risk factors for cardiovascular disease (16, 17)  The outcome was cardiovascular abnormalities detected through imaging (18)  The outcome was cardiovascular health Artero2012(19)  The outcome was CHD death, and also didn’t reported the required data (20)  The outcome is cardiac conduction disease (21) |
| **Participants:**  The participants are people newly diagnosed with diabetes; moreover, physical activity has been reported as change in physical activity (22)  The participants were veterans (23, 24)  Only athletes were included in the analysis(25)  Participants were hypertensive women Allesøe 2016  Participants were patients with high CV risk(26)  Participants were athlete (27) |
| Excluded because of the same cohort (duplicate, n=29)  Soares-Miranda(28)  Autenrieth et al.(29)  Veronesi 2018(59)  Willey 2009(30)  Allesøe 2017(31)  Chomestick 2015(32)  Chomestick 2013(33)  Lee 2001(34)  Alvarez 2018 (35)  Dı´az-Gutierrez(36)  Conroy 2005(37)  Ferrario 2019(38)  Wannamethee 1992 and 2000 (39, 40)  Ahmad 2010(41)  Lee 1999(42)  Li 2006(43)  Shaper 1991and 1994 (44, 45)  Bapat 2015(46)  Lee 2020 (47)  Su 2021(48)  Kurth 2006(49)  Mora 2007 (50)  Chiuve 2014 (51)  Harmsen 1990 (52)  Lapidus 1986 (53)  Evenson 1999 (54)  SALONEN 1982 (55) |
| **Insufficient data**  have no confidence intervals (56, 57, 58, 59)Poster, it doesn’t have sufficient data (60)  Relative risk and 95%CIs were not reported (56, 61)  Reported rate ratio (62)  The article had our required data, but didn’t report clearly which number related to which outcome and the author didn’t reply to our email contact.(63) |
| **Exposure:**  **Problem in unit of PA**  Lapidus 1986 study was excluded since the risk was reported in continuous with the unit (hour/week) that was not used by other studies which reported this outcome; moreover, Mantel-Haenszel Method was used for the analysis (53).  PA has been reported in min/week and continuous (per 300 min/week increase), since no study has reported this unit for stroke this study was excluded. (64)  **Other**  Self-assessed activity was evaluated (65)  Only change in PA was reported as the exposure(66). |
| **PA combined with other behaviors**  Reported the result in lifetime risk model, therefore, we couldn’t use the data(61)  PA was combined with other healthy behaviors as the exposure (67) |
| **Problem in type of PA**   - Only sedentary LPA has been assessed (68) - Although participants have been asked about both OPA and LTPA, the result has been reported for total PA (combined OPA and LPA) (69, 70, 71, 72, 73) - Only Bicycling has been assessed (74) - Only walking has been assessed (75) - The risk of CHD was reported for combination of LTPA and Lewis phenotypes(76). - Domestic activities and housework included in the measure of physical activity(77, 78, 79, 80) - Physical activities done in a home environment (81) - Only Leisure-time cross-country skiing was analyzed(82) - It is unclear what type of physical activity was measured(3, 83) (84, 85) - Different classes of OPA including administrators and professionals, manual workers, and self-employed was compared with non-manual workers (86) |
| **Short follow-up duration**  Millar & Chen 1999 study was excluded since adjusted odds ratios for two-year incidence of heart disease was reported. Because of too short duration reverse causality is probable (87). |
| **Other reason**  Byrne 2016 was excluded as this study didn’t adjust for any confounders (reported crude model and very low follow-up rate (26.4%(n=2,707) participated in all 10 years.) (88)  We couldn’t access to full texts (89, 90, 91, 92, 93, 94, 95, 96) |

| **Supplementary Table 3. List of articles used the same cohort data** |
| --- |
| **Cardiovascular Health Study (CHS): 3 publications**  Two publications assessed CVD (28, 97)(Greenlee and Soares-Miranda); two publications assessed both CHD/MI and stroke(98) (Patel 2013 and Soares-Miranda)  Excluded studies:   - Soares-Miranda study was excluded. |
| **Atherosclerosis Risk in Communities (ARIC): 7 publications**  Three publications assessed CVD (Bell 2013, Florido 2016, Porter 2019) (99, 100, 101); two article assessed CHD (102) (Bell 2013 and Cuthbertson 2019); three studies assessed stroke (Evenson 1999(54), Autenrieth 2013 (29), Cuthbertson 2019(102)), one assessed AF (Fletcher 2022(103))  Excluded studies:   - Florido study was excluded because of the fewer number of events (they assessed atherosclerotic CVD) - Porter 2019 was also excluded because the risk was reported only according to sport styles. - For non-linear dose response of LTPA, Florido 2016 was included instead of Cuthbertson 2019, because the level of PA was defined better in Florido study. - Autenrieth et al.(29) study was excluded, since Cuthbertson study has longer follow-up and a greater number of events - Bell was excluded from CHD analysis. |
| **Women’s Health Study: 8 publications**  Two study assessed CVD (Chomistek 2018 (104), Mora 2007(50)); 5 studies CHD (Lee 2001(34), Conroy 2005(37), Weinstein 2008(105), Mora 2007 (50), Chomistek 2018 (104)); one atrial fibrillation (Everett 2011)(106), two stroke (Kurth 2006(49), Chomistek 2018 (104))  Moreover, Chomestic 2013(33) and Ahmad 2010(41) used Women’s Genome Health Study which was derived from WHS.  Excluded study:   - Lee 2001 (34) and Conroy 2005 were excluded(37). - Mora 2007 was excluded from both CVD and CHD analysis. - Chomestic 2013 and Ahmad 2010(41) were excluded due to fewer number of events. - Chomestic 2018 was excluded for the CHD analysis, as Weinstein 2008 had more event cases. - Kurth 2006 was excluded. |
| **Women’s Health Initiative (WHI): 2 studies**   - One study assessed AF (Azarball 2014)(107), and one study assessed CHD, stroke, and CVD (108)(Chomistek 2013) |
| **Nurses’ Health Study: 6 publications**  Three studies assessed CHD (32, 43, 109, 110) (Manson 1999, Li 2006, Chomestick 2015 and 2016); one study MI and stroke: Elliott 2020(111); two studies (CVD Elliot 2020 (111)(Chiuve 2014)(51))  Excluded study:   - Li 2006, Chomestick 2015 and Manson 1999. Chiuve 2014 were excluded |
| **Copenhagen City Heart Study: 3 publications**  One study assessed atrial fibrillation (112)(Skielboe); One study MI (Holtermann 2012), one study CVD (Holtermann 2021) |
| **Swedish Mammography Cohort (SMC): 3 publications**  One study assessed AF (OPA, Drca 2021); one study stroke(113) (Larsson), one study MI (114)(Akesson 2007) |
| **Danish Nurse Cohort Study: 2 publications**  Two studies assessed IHD (31, 115)(Allesøe 2015 and Allesøe 2017)  Excluded study:   - Both studies have the same number of cases; Allesøe 2017 was excluded. |
| **Northern Manhattan Study: 2 publications**  Two studies assessed stroke (30, 116)(Willey 2009 and Willey 2017)  Excluded study:   - Willey 2009 (30) was excluded |
| **Health Professionals' Follow-up Study:** 3 **publications**  One study assessed CHD (Tanasescu 2002)(117) and two studies CVD (Chomiste 2012(118), Chuive 2014(51))  Excluded study:   - Chuive 2014 was excluded(51). |
| **Seven independent population surveys in 6 geographic areas of Finland: 3 publications**  Two studies assessed CHD (SALONEN 1982(55), Hu 2007(119)); Two studies Stroke (SALONEN 1982(55), Hu 2005 (120))   - Salonen 1982 was excluded. |
| **Population Study of Women in Gothenburg: 3 publications**  Three studies assessed stroke (121) (Lapidus 1986(53), Blomstrand 2014 and Blomstrand 2022(122))  Excluded study:   - Although Blomstrand 2022(122) had longer follow-up duration, it was excluded from the LTPA since it adjusted for only two confounders. But it was included in OPA analysis, since Blomstrand 2014(121) didn’t reported OPA |
| **Malmo¨ Diet and Cancer Study (MDCS): 4 publications**  One study assessed stroke (123)(Johansson, 2021); one study stroke and coronary events (Calling 2006)(124); one study atherosclerotic CVD(125) (Acosta 2021), one study assessed relationship between OPA and stroke (Johansson 2022(126))  Excluded study:   - Calling 2006 was excluded from stroke analysis but included in CHD analysis. |
| **SUN Project (Seguimiento Universidad de Navarra, University of Navarra Follow-up): 3 publications**  All three studies that used this data assessed CVD incidence (Dı´az-Gutierrez 2017, Alvarez 2018, Hidalgo-Santamaria 2018) (35, 36, 127). Hidalgo-Santamaria also reported the risk for MI and stroke, separately.  Excluded studies:   - Dı´az-Gutierrez 2018 and Alvarez 2018 were excluded. |
| **Physician's Health Study: 2 publications**  Both two studies assessed stroke (Lee 1999 and Rist 2021)  Excluded studies:   - Lee 1999 was excluded (42) |
| **BELSTRESS: 2 publications**  Two studies assessed CHD (Clay 2013(128) and Clay 2016(129))  Excluded study:   - Both with the same follow-up, therefore, Clay 2016 was excluded (129) |
| **British Regional Heart Study: 6 publications**  Two studies assessed stroke (Wannamethee 1992(39), Ahmed 2020(130)), and three assessed CHD (Shaper 1991, Shaper 1994, Wannamethee 2000, Jefferis, 2014)(40, 44, 45, 131), one assessed CVD(Ahmed 2020(130))  Excluded study:   - Shaper 1991 and 1994 and Wannamethee 1992 and 2000 were excluded |
| **Multi-Ethnic Study of Atherosclerosis (MESA): 3 publications**  Two studies assessed AF (Mokhayeri 2018, Bapat 2015)(46), one CVD (Thomas 2020) |
| **Korean National Health Insurance Service (KNHIS) databases: 6 Publications**  Three study assessed cerebrovascular disease/stroke (Jeong 2017, Su 2021(48)), two AF (Jin 2019, Lee 2020(47)), one MI and stroke (Kim 2019), one CVD (Yang 2021)  Excluded studies:   - Su 2021(48), since Jeong 2017 defined categories of activity but Su 2021 didn’t - Lee 2020 was excluded since it didn’t define categories of activity(47) |
| **Rotterdam Study: 2 publications**  One study assessed CHD (Koolhaas 2016) and one assessed AF (Albrecht, 2018) |
| **Multifactor Primary Prevention Study: 2 Publications**  Both 2 studies assessed stroke (Harmsen 1990(52) and 2006(132))   - Harmesen 1990 was excluded |
| **PAMELA, MONICA and SEMM**  Veronesi 2018(133) used data of 2 cohorts PAMELA + MONICA, and Ferrario 2018 (134)also used data of these two cohorts in addition to another cohort. Therefore, Veronesi 2018 was excluded.  Ferrario 2018 and Ferrario 2019(38), both used data of these 3 cohorts. Therefore, Ferrario 2019 was excluded. |

**Supplementary Table 4. List of studies that did not report required data for dose-response analysis according to reasons**

| First author, year | Outcome |
| --- | --- |
| **Studies that didn’t report the number of participants and events in each category (n=11 studies, CVD: 5, CHD: 4, stroke: 9)** | |
| Soares-Miranda 2016(28) | CHD, CVD, Stroke |
| Lakka 1994(135) | CHD |
| CALLING 2006(124) | CHD, stroke, CVD |
| Larsson 2014(113) | stroke |
| Willey 2017 NOMAS (116) | Stroke |
| Willey 2017 CTS (136) | Stroke |
| Blomstrand 2014(121) | Stroke |
| Agnarsson 1999 (137) | Stroke |
| Harmsen 2006 (132) | Stroke |
| Lear 2017(138) | CVD |
| **Studies that didn’t quantify PA in the categories (n=22, CVD: 5, CHD:12, stroke: 7, AF: 2)** | |
| Wagner 2002(139) | CHD |
| Allesøe 2015(115) | CHD |
| Petersen 2012(140) | CHD |
| Merry 2011(141) | CHD |
| Rosolva 1994(142) | CHD |
| Kaprio 2000(143) | CHD |
| Kuster 2021(144) | CVD |
| Paudel 2023(145) | CVD |
| Garnvik, 2018(146) | AF |
| Johansen 2022(147) | AF |
| Gillum 1996 (148) | stroke |
| Hu 2005(120) | Stroke |
| Johansson 2022(126) | Stroke |
| Hu 2007(119) | CHD |
| Hu 2014(149) | CVD |
| Hummel 2022(150) | CHD, CVD, stroke |
| Johansson, 2021(123) | Stroke |
| Kaprio, 2000(143) | CHD |
| Sieverdes 2015(151) | Stroke |
| Strippoli, 2022(152) | CHD |
| Sundquist, 2005(153) | CHD |
| Zhao 2020(154) | CVD, CHD, stroke |
| **Reported PA in score or index (n=3, CVD: 2, CHD: 1, stroke: 1)** | |
| Jefferis 2014(131) | CHD |
| Bell 2013(99) | CVD |
| Ahmed 2020(130) | CVD, stroke |
| Abbreviation: CHD, coronary heart disease; CVD, cardiovascular disease; PA, physical activity | |

**Supplementary Table 5. Characteristic of studies investigating the relationship between LTPA and CVD**

| **Author, year** | **Country** | **Study name** | **Population characteristics** | **Total participants, Type of CVD (num)** | **Sex, Duration (Year)** | **Age (range or mean)** | **Adjustment factors** | **Type of PA** | **PA level** | **RR (95%CI)** | **Exposure assessment** | **Outcome assessment** | **PA timed assessed (Baseline, repeated)** |
| --- | --- | --- | --- | --- | --- | --- | --- | --- | --- | --- | --- | --- | --- |
| Acosta 2021(125) | Sweden | Malmö Diet and Cancer study | Middle-aged individuals | 26990/ ACVD: 5858 | B, 21.1 | 57.3 | Sex, age, alcohol, smoking, BMI, HTN, DM, education, diet, EI | LTPA | <7.5 MET-h/wk | 1.00 | Minnesota LTPA Questionnaire(validated) | National Patient register  (The diagnoses of CVDs  were separately validated) | Baseline |
|  |  |  |  |  |  |  |  |  | 7.5–15 MET-h/wk | 0.92(0.83, 1.02) |  |  |  |
|  |  |  |  |  |  |  |  |  | 15.1–25.0 MET-h/wk | 0.77 (0.70, 0.85) |  |  |  |
|  |  |  |  |  |  |  |  |  | 25.1–50.0 MET-h/wk | 0.81 (0.73, 0.88) |  |  |  |
|  |  |  |  |  |  |  |  |  | >50 MET-h/wk | 0.82 (0.74, 0.91) |  |  |  |
| Ahmed 2020(130) | UK | British Regional Heart Study | General population | 3616, CVD: 984 | B, 16 | 68.6 | Age, social class and alcohol intake | LTPA | High vs. low | 0.60 (0.49, 0.75) | Validated Questionnaire | Medical records | Baseline |
| Amidei 2022(155) | Italy | Progetto Veneto Anziani | Elderly population | 2321, CVD:1037, CHD:676, stroke:420 | B, 7 | > 65 | Sex, education, household members, smoking, alcohol, some chronic diseases (cancer, COPD, asthma, DM, CKD, Parkinson’s disease), CVD (CHD, HF, stroke, angina, TIA, AF, HTN and PAD with the exclusion of interested outcome at baseline, yr of birth | Moderate to vigorous LTPA, male | Active vs. low inactive | 0.74 (0.58, 0.94) | Validated questionnaire | Electronic health records | Repeated |
|  |  |  |  |  |  |  |  | LTPA, female | High vs. low | 1.00 (0.85, 1.19) |  |  |  |
| Armstrong 2015(156) | UK | Million Women Study | General population | 1119239/CHD: 55685, Cerebrovascular Disease:19649 | F, 9 | 50- 64 | BMI-by-age, smoking-by-age, alcohol-by-age, and stratified by socioeconomic status and region | Total LTPA, CHD | Rarely/never  (inactive) | 1.00 | Non-validated questionnaire | Electronic health records | Repeated |
|  |  |  |  |  |  |  |  |  | At most once  per wk | 0.87 (0.85–0.88) |  |  |  |
|  |  |  |  |  |  |  |  |  | 2–3 times/wk | 0.84 (0.82–0.85) |  |  |  |
|  |  |  |  |  |  |  |  |  | 4–6 times/wk | 0.75 (0.73–0.78) |  |  |  |
|  |  |  |  |  |  |  |  |  | Daily | 0.83 (0.82–0.85) |  |  |  |
|  |  |  |  |  |  |  |  | Total LTPA, Cerebrovascular Disease | Rarely/never  (inactive) |  |  |  |  |
|  |  |  |  |  |  |  |  |  | At most once  per wk | 0.87 (0.84–0.90) |  |  |  |
|  |  |  |  |  |  |  |  |  | 2–3 times/wk | 0.80 (0.77–0.83) |  |  |  |
|  |  |  |  |  |  |  |  |  | 4–6 times/wk | 0.83 (0.79–0.88) |  |  |  |
|  |  |  |  |  |  |  |  |  | Daily | 0.88 (0.86–0.91) |  |  |  |
|  |  |  |  |  |  |  |  | Strenuous LTPA, CHD | Daily activity vs. Rarely/ never | 0.89 (0.84–0.93) |  |  |  |
|  |  |  |  |  |  |  |  | Strenuous LTPA, Cerebrovascular Disease | Daily activity vs. Rarely/ never | 0.96 (0.89–1.04) |  |  |  |
| Barengo, 2016(157) | Finland | National FINRISK | Elderly | 2456/ CVD: 416, stroke: 226 | B, 11.8 | 65- 74 | Age, sex, area, BMI, serum cholesterol, SBP, smoking, education, marital status, inability to perform PA | LTPA, all | Low | 1.00 | Self-administered questionnaire (non-validated) | National hospital discharge register | Baseline |
|  |  |  |  |  |  |  |  |  | Moderate | 0.73 (0.57, 0.95) |  |  |  |
|  |  |  |  |  |  |  |  |  | High | 0.58 (0.40, 0.86) |  |  |  |
|  |  |  |  |  |  |  |  | LTPA, men | Low | 1.00 |  |  |  |
|  |  |  |  |  |  |  |  |  | Moderate | 0.71 (0.51, 1.00) |  |  |  |
|  |  |  |  |  |  |  |  |  | High | 0.61 (0.38, 0.98) |  |  |  |
|  |  |  |  |  |  |  |  | LTPA, women | Low | 1.00 |  |  |  |
|  |  |  |  |  |  |  |  |  | Moderate | 0.74 (0.49, 1.11) |  |  |  |
|  |  |  |  |  |  |  |  |  | High | 0.49 (0.24, 1.00) |  |  |  |
| BakkerI 2021(158) | Netherland | Lifelines Cohort Study | General population | 142493, CVD:1019, | B, 6.8 | > 18 | Age, sex, income, education, alcohol, smoking, nutrient intake, fat, carbohydrate, kidney function, arrhythmia, hypothyroid, lung disease, osteoarthritis, and RA | LTPA | 0 MET-h/wk | 1.00 | Validated questionnaire | Hospital records | Baseline |
|  |  |  |  |  |  |  |  |  | 8 MET-h/wk | 0.91 (0.74, 1.15) |  |  |  |
|  |  |  |  |  |  |  |  |  | 24 MET-h/wk | 1.04 (0.7, 1.32) |  |  |  |
|  |  |  |  |  |  |  |  |  | 40 MET-h/wk | 0.94 (0.69,1.19) |  |  |  |
|  |  |  |  |  |  |  |  |  | 56 MET-h/wk | 0.84 (0.67,1.05) |  |  |  |
| Bell, 2013(99) | USA | Atherosclerosis Risk in Communities (ARIC) | General population | 13725/ CVD (fatal+ non-fatal): 3031 | B, 21 | 45- 64 | Age, sex, smoking, alcohol, education, diet, hormone therapy in female | Sport, African Americans, all | High vs. low | 0.74 (0.63, 0.88) | Validated Baecke questionnaire | Hospital records | Repeated |
|  |  |  |  |  |  |  |  | Sport, African Americans, men | High vs. low | 0.90 (0.69, 1.18) |  |  |  |
|  |  |  |  |  |  |  |  | Sport, African Americans, women | High vs. low | 0.64 (0.51, 0.79) |  |  |  |
|  |  |  |  |  |  |  |  | Sport, Caucasians, all | High vs. low | 0.88 (0.76, 1.03) |  |  |  |
|  |  |  |  |  |  |  |  | Sport, Caucasians, men | High vs. low | 0.85 (0.69, 1.06) |  |  |  |
|  |  |  |  |  |  |  |  | Sport, Caucasians, women | High vs. low | 0.91 (0.74, 1.12) |  |  |  |
| Chomistek, 2012(118) | US | Health Professionals Follow-up Study (HPFS) | Professional males | 51,533/ CVD: 4769 | M, 22 | 40-75 | parental history of MI and cancer ≤ age 60 yrs, smoking, aspirin, vitamin E supplement, intake of PUFA, trans fat, EPA+ DHA, and fiber, alcohol, pre-existing disease including DM, HTN, or hypercholesterolemia | LTPA (Total activity) | 0 MET-h/wk | 1.00 | validated self-reported questionnaire | Self-report | Repeated |
|  |  |  |  |  |  |  |  |  | 0.1 – 3.5 MET-h/wk | 1.06 (0.87, 1.30) |  |  |  |
|  |  |  |  |  |  |  |  |  | 3.6 – 8.8  MET-h/wk | 0.98 (0.80, 1.19) |  |  |  |
|  |  |  |  |  |  |  |  |  | 8.9 – 21  MET-h/wk | 0.89 (0.73, 1.07) |  |  |  |
|  |  |  |  |  |  |  |  |  | >21 MET-h/wk | 0.80 (0.66, 0.97) |  |  |  |
|  |  |  |  |  |  |  |  | LTPA (Vigorous activity) | 0 MET-h/wk | 1.00 |  |  |  |
|  |  |  |  |  |  |  |  |  | 0.1 – 3.5 MET-h/wk | 0.98 (0.90, 1.06) |  |  |  |
|  |  |  |  |  |  |  |  |  | 3.6 – 8.8  MET-h/wk | 0.91 (0.82, 1.00) |  |  |  |
|  |  |  |  |  |  |  |  |  | 8.9 – 21  MET-h/wk | 0.88 (0.80, 0.96) |  |  |  |
|  |  |  |  |  |  |  |  |  | >21 MET-h/wk | 0.78 (0.70, 0.86) |  |  |  |
|  |  |  |  |  |  |  |  | LTPA (Moderate activity) | 0 MET-h/wk | 1.00 |  |  |  |
|  |  |  |  |  |  |  |  |  | 0.1 – 3.5 MET-h/wk | 0.85 (0.78, 0.93) |  |  |  |
|  |  |  |  |  |  |  |  |  | 3.6 – 8.8  MET-h/wk | 0.90 (0.82, 0.98) |  |  |  |
|  |  |  |  |  |  |  |  |  | 8.9 – 21  MET-h/wk | 0.86 (0.78, 0.95) |  |  |  |
|  |  |  |  |  |  |  |  |  | >21 MET-h/wk | 0.80 (0.72, 0.88) |  |  |  |
| Chomistek, 2013(108) | Us | WHI-OS | postmenopausal women | 71,018 / CVD: 4235, CHD: 2411, stroke: 2050 | F, 12.2 | 50 – 79 | The multivariable model was stratified by age and includes sedentary time and PA simultaneously, race, education, income, marital status, smoking, FH of MI, depression, alcohol, sleep, total calories, saturated fat, fiber | LTPA | ≤ 1.7 MET-h/wk | 1.00 | validated self-reported questionnaire | Self-report + Medicare and Medicaid Services data which were available only for some participants | Repeated |
|  |  |  |  |  |  |  |  |  | 1.8 – 8.3  MET-h/wk | 0.91 (0.82, 1.01) |  |  |  |
|  |  |  |  |  |  |  |  |  | 8.4 – 20  MET-h/wk | 0.84 (0.76, 0.93) |  |  |  |
|  |  |  |  |  |  |  |  |  | > 20  MET-h/wk | 0.74 (0.67, 0.82) |  |  |  |
| Chomistek, 2018(104) | USA | Women’s Health Study | Female health professionals | 27536/ CVD: 1860, CHD: 458, stroke: 650 | F, 19.1 | ≥45 | Age, randomized treatment assignment, smoking, alcohol, saturated fat, fiber, fruits, vegetables; menopausal status; postmenopausal hormone use, FH of MI <60 years, PA | LTPA | 0-199 kcal/wk | 1.00 | validated self-reported questionnaire | Medical records | Repeated |
|  |  |  |  |  |  |  |  |  | 200–599  kcal/wk | 0.93 (0.75–1.15) |  |  |  |
|  |  |  |  |  |  |  |  |  | 600-1499 kcal/wk | 0.81 (0.66–1.00) |  |  |  |
|  |  |  |  |  |  |  |  |  | ≥1500  kcal/wk | 0.72 (0.57–0.90) |  |  |  |
| Clara, 2021(159) | Spain | REGICOR study | General population | 10876/ CVD (fatal+ non-fatal): 437 | B, 7.24 | 53.8 | Age, sex, survey, smoking, HDL-C and LDL-C, cholesterol treatment, SBP, anti-HTN treatment, glucose, DM treatment, BMI, FH of CVD | Light LTPA | Per 100 MET-min/d | 0.94 (0.83-1.08) | Minnesota  LTPA Questionnaire, validated | medical records (population-based MI registry, and  cross-checked these sources | Every 2 years |
|  |  |  |  |  |  |  |  | Moderate to vigorous LTPA | Per 100 MET-min/d | 0.95 (0.83-1.08) |  |  |  |
|  |  |  |  |  |  |  |  | Total LTPA | Per 100 MET-min/d | 0.91 (0.80-1.03) |  |  |  |
| Djousse, 2021(23) | USA | Million Veteran Program (MVP) | Veteran | 315,119/ CVD: 22,942 | B, 3.3 | 64.6 | Age, sex, race, education, BMI, smoking, alcohol, DASH score | LTPA | 0 MET-h/wk | 1.00 | NM about validation of questionnaire | Medical records | Baseline |
|  |  |  |  |  |  |  |  |  | 0.1–1.24  MET-h/wk | 0.87  (0.82, 0.92) |  |  |  |
|  |  |  |  |  |  |  |  |  | 1.25–3.80 MET-h/wk | 0.77  (0.73, 0.82) |  |  |  |
|  |  |  |  |  |  |  |  |  | 3.81–6.60 MET-h/wk | 0.77  (0.73, 0.82) |  |  |  |
|  |  |  |  |  |  |  |  |  | 6.61–30 MET-h/wk | 0.69  (0.65, 0.73) |  |  |  |
| Elliott, 2020(111) | USA | NHS | Nurse | 104990/ 6074 MI: 3,304 or stroke: 2,848 | F, 20 | 30–55 | Age, race, cancer, FH of MI, smoking, AHEI score, multivitamin, alcohol, census tract, income, home value, occupation of father and mother, husband’s education, registered nursing degree, marital status, retirement status | LTPA, overall | <3.7  MET-h/wk | 1.00 | Validated questionnaire | Medical records | Repeated |
|  |  |  |  |  |  |  |  |  | 3.7–10.8 MET-h/wk | 0.74(0.70,0.80) |  |  |  |
|  |  |  |  |  |  |  |  |  | 10.9–24.3 MET-h/wk | 0.69(0.64,0.74) |  |  |  |
|  |  |  |  |  |  |  |  |  | ≥24:4  MET-h/wk | 0.61(0.57,0.66) |  |  |  |
|  |  |  |  |  |  |  |  | LTPA, Vigorous | <1.4  MET-h/wk | 1.00 |  |  |  |
|  |  |  |  |  |  |  |  |  | 1.4 – 6.2  MET-h/wk | 1.04(0.88,1.22) |  |  |  |
|  |  |  |  |  |  |  |  |  | 6.3- 13.4  MET-h/wk | 0.96(0.80, 1.14) |  |  |  |
|  |  |  |  |  |  |  |  |  | ≥13.5  MET-h/wk | 0.80(0.67, 0.96) |  |  |  |
| Ekblom-Bak, 2021(160) | Sweden | People living in Stockholm, born between 1 July 1937–30 June 1938 | 60yr old women and men with no metabolic syndrome | 3693/ CVD: 641 | B, 20 | 60 | sex, smoking habits, education, alcohol, intake of fruit and vegetables, % of linoleic acid and % of EPA of total fatty acids, and CVD heredity. | LTPA, Metabolic syndrome, Overall | 0 MET-h/wk | 1.00 | validated self-reported questionnaire | National  Patient Register | Baseline |
|  |  |  |  |  |  |  |  |  | 6 MET-h/wk | 0.71 (0.50, 1.00) |  |  |  |
|  |  |  |  |  |  |  |  |  | 16.5 MET-h/wk | 0.73 (0.49, 1.09) |  |  |  |
|  |  |  |  |  |  |  |  | LTPA, Metabolic syndrome, Male | 0 MET-h/wk | 1.00 |  |  |  |
|  |  |  |  |  |  |  |  |  | 6 MET-h/wk | 0.74 (0.47, 1.18) |  |  |  |
|  |  |  |  |  |  |  |  |  | 16.5 MET-h/wk | 0.72 (0.43, 1.21) |  |  |  |
|  |  |  |  |  |  |  |  | LTPA, Metabolic syndrome, Female | 0 MET-h/wk | 1.00 |  |  |  |
|  |  |  |  |  |  |  |  |  | 6 MET-h/wk | 0.66 (0.39, 1.11) |  |  |  |
|  |  |  |  |  |  |  |  |  | 16.5 MET-h/wk | 0.85 (0.45, 1.62) |  |  |  |
|  |  |  |  |  |  |  |  | LTPA, No metabolic syndrome, Overall | 0 MET-h/wk | 0.47 (0.31, 0.72) |  |  |  |
|  |  |  |  |  |  |  |  |  | 6 MET-h/wk | 0.44 (0.32, 0.60) |  |  |  |
|  |  |  |  |  |  |  |  |  | 16.5 MET-h/wk | 0.40 (0.29, 0.57) |  |  |  |
|  |  |  |  |  |  |  |  | LTPA, No metabolic syndrome, Male | 0 MET-h/wk | 0.58 (0.33, 1.01) |  |  |  |
|  |  |  |  |  |  |  |  |  | 6 MET-h/wk | 0.51 (0.33, 0.78) |  |  |  |
|  |  |  |  |  |  |  |  |  | 16.5 MET-h/wk | 0.48 (0.31, 0.76) |  |  |  |
|  |  |  |  |  |  |  |  | LTPA, No metabolic syndrome, Female | 0 MET-h/wk | 0.36 (0.19, 0.69) |  |  |  |
|  |  |  |  |  |  |  |  |  | 6 MET-h/wk | 0.36 (0.22, 0.58) |  |  |  |
|  |  |  |  |  |  |  |  |  | 16.5 MET-h/wk | 0.30 (0.17, 0.51) |  |  |  |
| Elwood 2013(161) | UK | Caerphilly Cohort Study | General population | 2,235, CVD:752 | M, 30 | 45–59 | Age, social class | LTPA, Regular exercise | Yes vs. no | 0.89 (0.74, 1.07) | Non-validated questionnaire | Hospital records | Repeated |
| Fernandez‑Lazaro 2022(162) | Spain | Vascular Risk in Navarra Study (RIVANA) | General population | 3826/CVD: 194 | B, 12.8 | 52.8 | Age, sex, smoking, BMI, healthy diet, higher level of attained education, and occupation | LTPA | ≥150 min/wk or equivalent combination | 0.93 (0.59, 1.46) | validated Spanish version of the Minnesota Leisure Time Physical Activity Questionnaire | primary electronic health record database, hospital discharge database, population-based MI registry, and regional and national mortality registries | Baseline |
| Ferrario, 2018(134) | Italy | 3 population-based cohorts (the MONICA Brianza surveys II & III; the PAMELA; and one factory-based (SEMM) | Men working as salaried employees | 3574/ CVD: 174 | M, 14 | 25–64 | Age, cohort, educational level, BMI, total and HDL cholesterol, SBP, current smoking, DM and alcohol intake | Sport, OPA | Poor (0 MET-h/wk) | 1.00 | Baecke Questionnaire  (validated) | Hospital records | Baseline |
|  |  |  |  |  |  |  |  |  | Intermediate (5.3 MET-h/wk) | 0.81 (0.51, 1.27) |  |  |  |
|  |  |  |  |  |  |  |  |  | Recommended 22.7 MET-h/wk | 0.72(0.39, 1.32) |  |  |  |
| Ford, 2009 (163) | German | EPIC–Potsdam  study | General population | 23153, MI: 214, stroke: 195 | B, 7.8 | 49.3 (35-65) | Age, sex, education, occupational status, BMI, diet, smoking, | sports and bicycle riding, ≥ 3.5 h/wk | Yes vs. no | 0.74 (0.46, 1.03) | NM about validation | Medical records | Baseline |
| Fu 2018(164) | Sweden | Gothenburg Cohort of men born in 1913 and 1943 | 50-year-old men | 798, CVD:180 | M, 21 | > 50 year | Smoking, serum cholesterol, HTN, SBP, DM, BMI | LTPA | Active vs. sedentary | 0.63 (0.46, 0.87) | Saltin–Grimby questionnaire | National Health Registry, review of medical records, and the screening examinations | Baseline |
| Greenlee, 2017(97) | USA | Cardiovascular Health Study (CHS) | Older adult | 3491/ CVD: 1978 | B, 15 | 65–98 | Age, sex, self-rated health, smoking, Race/ethnicity, income, education, marital status, NSAID drug, limitations in instrumental activities of daily living | LTPA | Ideal vs. poor | 0.76 (0.65, 0.90) | Minnesota Leisure-Time Physical Activity Questionnaire  (Validated) | Hospital records | Baseline |
| Gunnell, 2014(165) | Australia | Busselton Health Study | General population | 2320/ fatal or non-fatal CVD events: 682 | B, 15 | 45–84 | Age, gender, marital status, occupation, smoking, alcohol, DM, history of COPD, BMI, SBP, history of HTN treatment, cholesterol, HDL, glucose, CRP | LTPA | > 150 minutes per week vs. < 150 minutes per week | 0.77 (0.66, 0.92) | self-reported questionnaire (validated unclear) | Hospital records | Baseline |
| Hidalgo-Santamaria, 2018(127) | Spain | SUN Project (Seguimiento Universidad de Navarra, University of Navarra Follow-up) | General population | 18737/ CVD (fatal+ nonfatal): 127, non-fatal MI:62, stroke:33 | B, 10.3 | 38 | Age, sex, BMI, EI, alcohol, adherence to Mediterranean diet, smoking, education, FH of CHD, TG, HDL-C, HTN, DM, cancer, EE in LTPA and changes in PA in the 2th and 4th year follow-up, antiaggregant & anticoagulant treatment, with age and year of entering the cohort as stratification variables | LTPA | Inactive | 1.0 | Validated 17-item self-administered questionnaire previously | Medical record | Baseline |
|  |  |  |  |  |  |  |  |  | <6 Average METs | 0.76(0.48−1.21) |  |  |  |
|  |  |  |  |  |  |  |  |  | ≥6 Average METs | 0.31(0.12−0.79) |  |  |  |
| Hoevenaar-Blom, 2010(166) | Netherland | Dutch Monitoring Project on Risk Factors for  Chronic Diseases (MORGEN) Study | General population | 16442, CVD (fatal and non-fatal: 362 | B, 9.8 | 20–65  (41.9) | Age, sex, other physical activities (both  occupational and leisure), smoking, alcohol, education | Sports | Yes. vs. no | 0.74 (0.64–0.87) | validated questionnaire designed for  the EPIC | Hospital admission  (Validated by another  study with an approximate 33% overlap of participants  from this study | Baseline |
| Hu, 2014(149) | Taiwan | Chin-Shan Community Cardiovascular Cohort study | General population | 1706/ CVD: 215 | B, 16.8 | 55.3 | Age, sex, BMI, marital status, job, education, other type PA, smoking, alcohol, HTN and DM at the baseline, SBP, DBP, TG, uric acid, FBS, lipid profiles | LTPA, OPA | Lowest tertile of LTPA | 1.00 | Baecke questionnaire | Imaging data and medical records | Repeated |
|  |  |  |  |  |  |  |  |  | Middle tertile of LTPA | 0.65 (0.46, 0.92) |  |  |  |
|  |  |  |  |  |  |  |  |  | Highest tertile of LTPA | 0.89 (0.63-1.25) |  |  |  |
| Hummel, 2022(150) | Sweden | Swedish National March Cohort (SNMC) | General population | 31580/ Stroke + MI: 3500 | B, 17.9 | 48.2 | Age, smoking, alcohol, education, BMI, HTN, DM, and lipid disturbance | LTPA, MI, Total | High vs. low | 0.79 (0.66–0.94) | Validated questionnaire | National inpatient and outpatient register | Baseline |
|  |  |  |  |  |  |  |  | LTPA, MI, Male | High vs. low | 0.78 (0.62–0.98) |  |  |  |
|  |  |  |  |  |  |  |  | LTPA, MI, Female | High vs. low | 0.80 (0.61–1.04) |  |  |  |
|  |  |  |  |  |  |  |  | LTPA, stroke, Total | High vs. low | 0.86 (0.73–1.00) |  |  |  |
|  |  |  |  |  |  |  |  | LTPA, stroke, Male | High vs. low | 0.78 (0.61–0.99) |  |  |  |
|  |  |  |  |  |  |  |  | LTPA, stroke, Female | High vs. low | 0.91 (0.74–1.13) |  |  |  |
| Holtermann, 2021(167) | Denmark | Copenhagen General Population Study | General population | 75195/ Major adverse CVE: 5813 | B, 10 | 20–100 | Age, sex, education, BMI, smoking, DM, SBP, BP medication, alcohol, dietary preferences, LDL-C, HDL-C, TG, COPD; and an extended multivariable sensitivity, resting heart rate, vital exhaustion score, occupation, cohabitation, marital status, income | LTPA, OPA | 0 Met-h/wk | 1.00 | Question  (non-validated) | Hospital record | Baseline |
|  |  |  |  |  |  |  |  |  | 6 Met-h/wk | 0.90 (0.80, 1.00) |  |  |  |
|  |  |  |  |  |  |  |  |  | 24 Met-h/wk | 0.82 (0.73, 0.91) |  |  |  |
|  |  |  |  |  |  |  |  |  | 40 Met-h/wk | 0.91 (0.78, 1.05) |  |  |  |
| Jasiukaitien˙e 2020(168) | Lithuania | Health, Alcohol and Psychosocial Factors in Eastern Europe (HAPIEE) | Urban Population | 4257, CVD: 458 | M, 11 | 45- 72 | Age, metabolic syndrome, nutrition factors (fresh vegetables and fruits, chicken, fish, eggs, high intake of meat and low cereals), smoking, education, respondents who  received treatment for DM and/or dyslipidemia were excluded | LTPA, men | Physically active vs. physically inactive | 0.85 (0.66–1.09) | Self-reported questionnaire, not validated | NM | Baseline |
|  |  |  |  |  |  |  |  | LTPA, women | Physically active vs. physically inactive | 1.13 (0.74–1.73) |  |  |  |
| Khaw 2006(71) | UK | EPIC-Norfolk | General population | 22191/ CVD (fatal+ non-fatal): 3079 | B, 8 | 45–79 | Age, Sex, BMI, SBP, cholesterol, smoking, alcohol, known diabetes, and social class | LTPA, OPA | None | 1.00 | Two questions  Validated | hospital admission  and/or died with CVD as underlying cause of death | Baseline |
|  |  |  |  |  |  |  |  |  | Up to 0.5 h | 0.96 (0.88–1.05) |  |  |  |
|  |  |  |  |  |  |  |  |  | 0.5–1 h daily | 0.95 (0.84–1.09) |  |  |  |
|  |  |  |  |  |  |  |  |  | More than 1 h | 0.76 (0.64–0.94) |  |  |  |
| Kuster, 2021(144) | Sweden | Attitude, Behavior, and Change Study | General population | 807/ CVD: 59 | B, 14.6 | 42.8 (18-75) | Age, sex, education and previous disease (HTN, DM, heart disease, cancer, arthritis) | LTPA, OPA | Active vs. sedentary | 0.74 (0.41–1.32) | Self-report questionnaire  (Validated) | National patient register | Baseline |
| Lacey, 2015(169) | Australia | The Health in Men Study | General population without prior vascular disease | 7564/ major CVE: 1557, IHD: 833, stroke: 551 | M, 11 | 72 | age at risk, education, smoking, BMI, place of birth, marital status, alcohol, frequency of salt addition to food, HTN medication, DM, cholesterol-lowering medication | Recreational PA | 0 Met-h/wk | 1.0 | Questionnaire (NM for validation) | Hospital record | Baseline |
|  |  |  |  |  |  |  |  |  | 1.14 Met-h/wk | 0.88 (0.79, 1.00) |  |  |  |
|  |  |  |  |  |  |  |  |  | 15-24 Met-h/wk | 0.81 (0.72, 0.91) |  |  |  |
|  |  |  |  |  |  |  |  |  | 25-39 Met-h/wk | 0.81 (0.72, 0.91) |  |  |  |
|  |  |  |  |  |  |  |  |  | ≥ 40 Met-h/wk | 0.80 (0.71, 0.89) |  |  |  |
| Lear 2017(138) | 17 countries: Canada, Sweden, UAE, Argentina, Brazil, Chile, Poland, Turkey, Malaysia, South Africa, China, Colombia, Iran,  Bangladesh, India, Pakistan,  Zimbabwe | PURE study | General population | 130843, CVD (fatal + non-fatal): 5753 | B, 6.9 | 35-70, 50 | age, sex, education, country income level, urban or rural residency, FH of CVD, smoking, taking into account household, community, and country clustering | LTPA | High vs. low | 0.93 (0.80, 1.08) | IPAQ, validated | Multiple approach (self-report, medical record, other sources of information) | Baseline |
| Meneton, 2016(170) | France | GAZEL cohort | Middle-aged volunteers working at the French  National Gas and Electricity Company | 10736/CVD: 1694 | B, 20 | 35–50 | Age, sex, diabetes, smoking, BMI, HTN, alcohol, Parental CVD, dyslipidemia, depression, sleep disorders | practice of a sport | Yes vs. no | 0.81 (0.73–0.90) | Questionnaire (NM for validation) | Self-reported | Repeated  (but we used baseline) |
| Mu, 2021(171) | China | Dongfeng‑Tongji cohort | General population | 26584/ CVD: 5704 | B, 5 | 63.3 | Age, sex, education, smoking, alcohol, consumption of food (meat, vegetables, and fruit), HTN, hyperlipidemia, DM, BMI, MET-hours/week, total sedentary time, and FH of CVD | LTPA | < 7.5 Met-h/wk | 1.00 | Questionnaire (NM about validation) | Hospital record | Baseline |
|  |  |  |  |  |  |  |  |  | 7.5 to < 22.5 Met-h/wk | 0.93 (0.86, 1.01) |  |  |  |
|  |  |  |  |  |  |  |  |  | 22.5 to < 37.5  Met-h/wk | 0.82 (0.75, 0.91) |  |  |  |
|  |  |  |  |  |  |  |  |  | ≥ 37.5  Met-h/wk | 0.81 (0.73, 0.90) |  |  |  |
| Paudel 2023(145) | UK | UK Biobank | White ethnic background | 328228, CVD: 98922 | B, 12.1 | 55.9 | Age, sex, ethnicity, sleep score, dietary pattern score, smoking and alcohol consumption, screening time | LTPA | high IPAQ_MVPA vs. low IPAQ_MVPA | 1.02 (1.0, 1.03) | Weekly self-reported MVPA and Accelerometer-measured MVPA | Record linkage | Baseline |
| Sesso, 1999(172) | USA | Pennsylvania College Alumni Health Study | Middle-aged and Older Women | 1564, CVD (fatal+ non-fatal): 181 | F, 20 | 37- 69 | Age, BMI, HTN, DM, smoking, FH of CHD, and total PA | Sport | None | 1.00 | A valid questionnaire | Self-report | Baseline |
|  |  |  |  |  |  |  |  |  | 1- 999 Kcal/wk | 0.99 (0.69, 1.41) |  |  |  |
|  |  |  |  |  |  |  |  |  | > 1000 Kcal/wk | 0.88 (0.62, 1.25) |  |  |  |
| Stamatakis, 2009(173) | Scotland | Scottish Health Survey | General population | 6033/ CVD (fatal + non-fatal): 468 | B, 8.4 | ≥35 | Age, sex, social economic group, marital status, BMI, longstanding illness, self-rated health, family disease history, smoking, and other types of PA | LTPA, men | None | 1.00 | Validated questionnaire | linked to the patient-based  database of hospital episodes and deaths | Baseline |
|  |  |  |  |  |  |  |  |  | 1 session/ wk | 1.10 (0.99, 1.35) |  |  |  |
|  |  |  |  |  |  |  |  |  | 2.5 session/ wk | 0.76 (0.58, 0.98) |  |  |  |
|  |  |  |  |  |  |  |  | LTPA, women | None | 1.00 |  |  |  |
|  |  |  |  |  |  |  |  |  | 1 session/wk | 0.80 (0.61, 1.03) |  |  |  |
|  |  |  |  |  |  |  |  |  | 2.5 session/ wk | 0.68 (0.50, 0.93) |  |  |  |
|  |  |  |  |  |  |  |  | Sport, men | None | 1.00 |  |  |  |
|  |  |  |  |  |  |  |  |  | 1 session/ wk | 0.70 (0.60, 1.06) |  |  |  |
|  |  |  |  |  |  |  |  |  | 2.5 session/ wk | 0.67 (0.44, 1.03) |  |  |  |
|  |  |  |  |  |  |  |  | Sport, women | None | 1.00 |  |  |  |
|  |  |  |  |  |  |  |  |  | 1 session/ wk | 0.82 (0.54, 1.25) |  |  |  |
|  |  |  |  |  |  |  |  |  | 2.5 session/ wk | 0.71 (0.42, 1.18) |  |  |  |
| Thomas, 20  20(174) | USA | MESA | General population | 3393, Hard CVD (MI, resuscitated cardiac arrest,  CHD death, stroke, or stroke death):520 | B, 13.7 | 45- 84 (66.3) | age, sex, race/ethnicity, income, education, mutual adjustment for the two PA variables, coronary artery calcium volume and density,  SBP, HTN medication, total and HDL-C, statin medication, smoking,  DM, BMI | recreational PA | per 2395 METmin/  week | 0.88 (0.79–0.98) | MESA typical week PA survey was used to assess the time spent and frequency of various PAs during a typical week in past month. (validated) | Medical records | Baseline |
| van Sloten, 2018(175) | UK | Whitehall II study | employed by the civil service | 9256, CVD: 2052 | B, 18.9 | 33- 55 | Sex, age, race/ethnicity, depression, education, occupation and family history of CVD | LTPA | None | 1.00 | Non-validated questionnaire | Electronic health records | Repeated |
|  |  |  |  |  |  |  |  |  | 1–74 min/wk vigorous PA, 1-149 min/wk moderate PA or a combination of the two | 0.84 (0.73, 0.95) |  |  |  |
|  |  |  |  |  |  |  |  |  | ≥75 min/wk of vigorous PA, ≥150 min/wk of moderate PA or a combination of the two | 0.88 (0.77, 1.01) |  |  |  |
| Yang, 2021(176) | Korea | Korea National Health Insurance Service–Senior | Elderly population | 218488, CVD: 20494 | B, 7.1 | > 60 | Sex, age, economic status, Hospital Frailty Score, living in metropolitan cities, and competing risk of death. | LTPA, Elderly (60~74 years) | None | 1.00 | Non-validated questionnaire | Hospital records | Baseline |
|  |  |  |  |  |  |  |  |  | 1–74 min/wk vigorous PA, 1-149 min/wk | 0.82 (0.77-0.87) |  |  |  |
|  |  |  |  |  |  |  |  |  | ≥75 min/wk of vigorous PA, ≥150 min/wk | 0.73 (0.68-0.78) |  |  |  |
|  |  |  |  |  |  |  |  | LTPA, Very-elderly (≥ 75 years) | None | 1.00 |  |  |  |
|  |  |  |  |  |  |  |  |  | 1–74 min/wk vigorous PA, 1-149 min/wk | 0.82 (0.72-0.93) |  |  |  |
|  |  |  |  |  |  |  |  |  | ≥75 min/wk vigorous PA, ≥150 min/wk | 0.80 (0.70-0.92) |  |  |  |
| Zhao, 2020(154) | China | Electronic health check-up database Xinzheng City | Elderly population | 32,942/ CVD: 6,857, stroke:2240, CHD: 4937 | B, 6.84 | ≥68 | Age, sex, current smoking, alcohol, BMI, medical history of T2DM, SBP, DBP, RHR, FPG, TC, TG and HDL-C | LTPA | Very highly active vs. inactive | 0.87 (0.71-1.06) | Questionnaire, NM about validation | data of the annual standard health check-up record  with digital linkage to the hospital dataset for admissions | Baseline |
| Abbreviations: AHEI, Alternate Healthy Eating Index; ASCVD, Atherosclerotic cardiovascular disease; B, both; BP, blood pressure; BMI, body mass index; CAD, coronary artery disease; CE, coronary events; CHD, coronary heart disease; CHF, congestive heart failure; CI, confidence interval; COPD, chronic obstructive pulmonary disease ; CRP, C-reactive protein; CVE, cardiovascular events; DBP, diastolic blood pressure; DM, diabetes mellitus; EE, energy expenditure; EI, energy intake; EPA, eicosapentaenoic acid; EPIC, European Prospective Investigation into Cancer and Nutrition; F, female; FBS, fasting blood sugar; FH, family history; HDL-C, high density lipoproteins cholesterol; HF, heart failure; HTN, hypertension; IHD, ischemic heart disease; LDL-C, low density lipoproteins cholesterol; LTPA, leisure time physical activity; M, Male; MONICA, Multinational Monitoring of Trends and Determinants in Cardiovascular Disease; MVPA, moderate-to-vigorous physical; NHIS, National Health Insurance Service; NM, not mentioned; NSAID, non-steroidal anti-inflammatory; IPAQ, international physical activity questionnaire; OPA, occupational physical activity; PA, physical activity; PAD, peripheral artery disease; PAMELA, Pressioni Arteriose Monitorate Loro Associazioni; PUFA, polyunsaturated fatty acid; PY, person-years; RR, risk ratio; SEMM, Study of Employed in the Municipality of Milan, SBP, systolic blood pressure; T-C, total cholesterol; TFA, trans fatty acids; TG, triglyceride; TIA, transient ischemic attack ; WC, waist circumference; WHI-OS, Women’s Health Initiative (WHI) Observational Study; wk, week | | | | | | | | | | | | | |

**Supplementary Table 6. Characteristic of studies investigating the relationship between LTPA and CHD**

| **Author, year** | **Country** | **Study name** | **Population characteristics** | **Total participants, Type of CVD (num)** | **Sex, Duration (Year)** | **Age (range or mean)** | **Adjustment factors** | **Type of PA** | **PA level** | **RR, 95% CI** | **Exposure assessment** | **Outcome assessment** | **PA timed assessed (Baseline, repeated)** |
| --- | --- | --- | --- | --- | --- | --- | --- | --- | --- | --- | --- | --- | --- |
| Allesøe, 2015(115) | Denmark | Danish Nurse Cohort Study | Nurses | 12093/ IHD: 580 | F, 15 | 45- 64 | Age, FH of IHD, HTN, DM, BMI, smoking, alcohol, LTPA, work pressure, job influence, shift work and work h/wk | LTPA,  OPA | Sedentary vs. vigorous | 1.50 (1.04-2.16) | Saltin and Grimby questionnaire | National patient  register | Baseline |
| Akesson 2007(114) | Sweden | Va¨stmanland and O¨rebro Cohort of  women born between 1914 -1948 | General population | 24444, MI:308 | F, 6.2 | 48- 83 | Age, education, FH of MI, presence of high cholesterol level or HTN, use of hormone therapy, or aspirin, total EI, dietary patterns, smoking, WHR | LTPA | Low risk (40 min/d of walking or bicycling  and 1 h/wk of exercise vs less) | 0.85 (0.65-1.10) | Validated questionnaire | Hospital records | Baseline |
| Akesson 2014(177) | Sweden | Va¨stmanland and O¨rebro Cohort of men born between 1918 and 1952 | General population | 20721, MI: 1361 | M, 11 | 45- 79 | Age, education, smoking, marital status, FH of MI, use of aspirin, non-Recommended Food Score, total EI, alcohol; abdominal obesity | LTPA | Low risk (40 min/day walking/bicycling  and 1 h vs. <1 h weekly exercise) | 0.97 (0.86–1.09) | Validated questionnaire | National Inpatient and Cause of Death Registers | Baseline |
| Amidei 2022(155) | Italy | Progetto Veneto Anziani | Elderly population | 2321, CVD:1037, CHD:676, stroke:420 | B, 7 | > 65 | Sex, education, age household members, smoking, alcohol, a number of chronic diseases (cancer, COPD, asthma, DM, CKD, Parkinson’s disease, etc.), CVD (CHD, HF, stroke, angina, TIA, AF, HTN and PAD with the exclusion of interested outcome) at baseline | Moderate to vigorous LTPA, male | Active vs. low inactive | 0.66 (0.50 to 0.87) | Validated questionnaire | Electronic health records | Repeated |
|  |  |  |  |  |  |  |  | Moderate to vigorous LTPA, female | Active vs. low inactive | 0.92 (0.75 to 1.14) |  |  |  |
| Armstrong 2015(156) | UK | Million Women Study | General population | 1119239/CHD: 55685, Cerebrovascular Disease:19649 | F, 9 | 50- 64 | BMI-by-age, smoking-by-age, alcohol-by-age, and stratified by socioeconomic status and region | Total LTPA, CHD | Rarely/never (inactive) | 1.00 | Non-validated questionnaire | Electronic health records | Repeated |
|  |  |  |  |  |  |  |  |  | At most once per wk | 0.87 (0.85-0.88) |  |  |  |
|  |  |  |  |  |  |  |  |  | 2–3 times/wk | 0.84 (0.82-0.85) |  |  |  |
|  |  |  |  |  |  |  |  |  | 4–6 times/wk | 0.75 (0.73-0.78) |  |  |  |
|  |  |  |  |  |  |  |  |  | Daily | 0.83 (0.82-0.85) |  |  |  |
|  |  |  |  |  |  |  |  | Strenuous LTPA, CHD | Daily activity vs. Rarely/ never | 0.89 (0.84-0.93) |  |  |  |
| Calling, 2006(124) | Sweden | Malmo¨ Diet and Cancer Study (MDCS) | General population | 26942/ CE:777,  ischemic stroke: 538 | B, 7.6 | 58.18 | age, height, smoking, alcohol, body fat%, DM, SBP, use of BP lowering drugs, and use of lipid-lowering drugs | LTPA, Low body fat % men | High PA (Q2–Q4) to low PA (Q1) | 0.93 (0.68-1.26) | Minnesota LTPA Questionnaire | Hospital records | Baseline |
|  |  |  |  |  |  |  |  | LTPA, High body fat% men | High PA (Q2–Q4) to low PA (Q1) | 0.75 (0.59-0.96) |  |  |  |
|  |  |  |  |  |  |  |  | LTPA, Low body fat %, women | High PA (Q2–Q4) to low PA (Q1) | 0.74 (0.44-1.24) |  |  |  |
|  |  |  |  |  |  |  |  | LTPA, High body fat% women | High PA (Q2–Q4) to low PA (Q1) | 0.66 (0.49-0.91) |  |  |  |
| Chomistek, 2013(108) | Us | WHI-OS | postmenopausal women | 71,018 / CVD: 4235, CHD: 2411, stroke: 2050 | F, 12.2 | 50 – 79 | The multivariable model was stratified by age and includes sedentary time and PA simultaneously, race, education, income, marital status, smoking, FH of MI, depression, alcohol, sleep, EI, saturated fat, fiber, HTN, DM, high T-C, BMI | LTPA | ≤ 1.7 MET-h/wk | 1.00 | validated self-reported questionnaire | Self-report + Medicare & Medicaid Services data which were available only for some participants | Repeated |
|  |  |  |  |  |  |  |  |  | 1.8 – 8.3 MET-h/wk | 1.20 (1.06-1.36) |  |  |  |
|  |  |  |  |  |  |  |  |  | 8.4 – 20 MET-h/wk | 1.28(1.13- 1.45) |  |  |  |
|  |  |  |  |  |  |  |  |  | > 20 MET-h/wk | 1.43 (1.25-1.63) |  |  |  |
| Chomistek, 2016(109) | US | NHS II | General population | 97230, CHD: 544 | F, 20 | 27-44 | Age, parental history of MI at age of <60 yrs, aspirin, menopausal status, postmenopausal hormone, parity, OCP | LTPA | <1 MET-h/wk | 1.00 | Validated questionnaire | Medical records | Repeated |
|  |  |  |  |  |  |  |  |  | 1–5.9 MET-h/wk | 0.96 (0.76-1.21) |  |  |  |
|  |  |  |  |  |  |  |  |  | 6- 14.9 MET-h/wk | 0.80 (0.63-1.03) |  |  |  |
|  |  |  |  |  |  |  |  |  | 15-29.9 MET-h/wk | 0.63 (0.47-0.83) |  |  |  |
|  |  |  |  |  |  |  |  |  | ≥ 30 MET-h/wk | 0.75 (0.57-0.99) |  |  |  |
| Chomistek, 2018(104) | USA | Women’s Health Study | Female health professionals | 27536/ CVD: 1860, CHD: 458, stroke: 650 | F, 19.1 | ≥45 | Age, randomized treatment assignment, smoking, alcohol, saturated fat, fiber, fruits, vegetables; PA; menopausal status; postmenopausal hormone use, FH of MI <60 y | LTPA | 0-199 kcal/wk | 1.00 | validated self-reported questionnaire | Medical records | Repeated |
|  |  |  |  |  |  |  |  |  | 200–599 kcal/wk | 0.72 (0.55-0.93) |  |  |  |
|  |  |  |  |  |  |  |  |  | 600-1499 kcal/wk | 0.64 (0.50-0.83) |  |  |  |
|  |  |  |  |  |  |  |  |  | ≥1500 kcal/wk | 0.66 (0.51-0.86) |  |  |  |
| Clays, 2013(128) | Belgium | BELSTRESS | General population | 14337/ CHD: 87 | M, 3.15 | 35–59 | Age, OPA, education, BMI, occupational class, job strain, smoking, alcohol, DM, SBP, T-C and HDL_C | LTPA | Moderate to high vs. low | 0.76 (0.43-1.32) | Job Content Questionnaire  (Validated) | Physician diagnosis | Baseline |
| Cuthbertson, 2019(102) | USA | Atherosclerosis Risk in Communities (ARIC) | General population | 13534/ Nonfatal CHD: 2426, stroke: 1144 | B, 27.2 | 45- 64 (54) | Age, sex, race by ARIC center, education, smoking, and alcohol intake | LTPA | No LTPA | 1.00 | Baecke questionnaire, validated | Diagnostic  algorithm and hospital records | Repeated |
|  |  |  |  |  |  |  |  |  | <13.2 MET h/wk | 0.92 (0.82-1.02) |  |  |  |
|  |  |  |  |  |  |  |  |  | ≥13.2 MET h/wk | 0.88 (0.79-0.97) |  |  |  |
| Djousse, 2021(23) | USA | Million Veteran Program (MVP) | Veteran | 315,119/ CHD: 19550 | B, 3.3 | 64.6 | Age, sex, race, education, BMI, smoking, alcohol, DASH score | LTPA | 0 MET h/wk | 1.00 | NM about validation of questionnaire | Medical records | Baseline |
|  |  |  |  |  |  |  |  |  | 0.1–1.24 MET-h/wk | 0.86 (0.81-0.91) |  |  |  |
|  |  |  |  |  |  |  |  |  | 1.25–3.80 MET-h/wk | 0.77 (0.72-0.82) |  |  |  |
|  |  |  |  |  |  |  |  |  | 3.81–6.60 MET-h/wk | 0.76 (0.71-0.81) |  |  |  |
|  |  |  |  |  |  |  |  |  | 6.61–30 MET-h/wk | 0.67 (0.63-0.71) |  |  |  |
| Elliott, 2020(111) | USA | NHS | Nurse | 104990/ 6074 MI: 3,304 or stroke: 2,848 | F, 20 | 30–55 | Age, race, cancer, FH of MI, smoking, AHEI score, multivitamin, alcohol, census tract, income, home value, occupation of father and mother, husband’s education, registered nursing degree, marital status, retirement status | LTPA, overall | <3.7 MET-h/wk | 1.00 | Validated questionnaire | Medical records | Repeated |
|  |  |  |  |  |  |  |  |  | 3.7–10.8 MET-h/wk | 0.76(0.70,0.83) |  |  |  |
|  |  |  |  |  |  |  |  |  | 10.9–24.3 MET-h/wk | 0.68(0.61,0.74) |  |  |  |
|  |  |  |  |  |  |  |  |  | ≥24.4 MET-h/wk | 0.64(0.58,0.71) |  |  |  |
|  |  |  |  |  |  |  |  | LTPA, Vigorous | <1.4 MET-h/wk | 1.00 |  |  |  |
|  |  |  |  |  |  |  |  |  | 1.4 – 6.2 MET-h/wk | 0.99(0.80,1.23) |  |  |  |
|  |  |  |  |  |  |  |  |  | 6.3- 13.4 MET-h/wk | 0.90(0.71,1.14) |  |  |  |
|  |  |  |  |  |  |  |  |  | ≥13.5 MET-h/wk | 0.76(0.59,0.97) |  |  |  |
| Ferrario, 2018(134) | Italy | 3 population-based cohorts (the MONICA Brianza surveys II & III; the PAMELA; and one factory-based (SEMM) | Men working as salaried employees | 3574/ CHD: 135 | M, 14 | 25–64 | Age, cohort, educational level, BMI, total and HDL cholesterol, SBP, current smoking, DM and alcohol intake | Sport, OPA | Poor (0 MET-h/wk) | 1.0 | Baecke Questionnaire  (validated) | Hospital records | Baseline |
|  |  |  |  |  |  |  |  |  | Intermediate (5.3 MET-h/wk) | 0.81 (0.51-1.27) |  |  |  |
|  |  |  |  |  |  |  |  |  | Recommended (22.7 MET-h/wk) | 0.72 (0.39-1.32) |  |  |  |
| Ford, 2009(163) | German | EPIC–Potsdam  study | General population | 23153, MI: 214, stroke: 195 | B, 7.8 | 49.3 (35-65) | Age, sex, education, occupational status, BMI, diet, smoking, | sports and bicycle riding, ≥ 3.5 h/wk | Yes vs. no | 1.03 (0.66-1.39) | NM about validation | Medical records | Baseline |
| Haapanen, 1997(178) | Finland | Census data of an industrial town and 2 rural municipalities in northeastern of Finland | middle-aged  (subjects with age >35 y) | 2840/ CHD: 183 | B, 10 | 35- 63 | Age, sex, smoking, alcohol, socioeconomic status, overweight, HTN, DM | Total LTPA, Males | High (>1500) | 1.0 | Self-administered questionnaire (validated unclear) | Self-reported doctor’s diagnoses | Repeated |
|  |  |  |  |  |  |  |  |  | Moderate (1101-1900) | 1.33 (0.78-2.27) |  |  |  |
|  |  |  |  |  |  |  |  |  | Low (0-1100) | 1.98 (1.22-3.23) |  |  |  |
|  |  |  |  |  |  |  |  | Total LTPA, females | High (>1900) | 1.0 |  |  |  |
|  |  |  |  |  |  |  |  |  | Moderate (901-1500) | 0.73 (0.38-1.39) |  |  |  |
|  |  |  |  |  |  |  |  |  | Low (0- 900) | 1.25 (0.72-2.15) |  |  |  |
|  |  |  |  |  |  |  |  | Vigorous LTPA, males | Less than once a wk vs. at least once a wk | 1.42 (0.92-2.17) |  |  |  |
|  |  |  |  |  |  |  |  | Vigorous LTPA, females | Less than once a wk vs. at least once a wk | 1.13 (0.62-2.07) |  |  |  |
| Hidalgo-Santamaria, 2018(127) | Spain | SUN Project (Seguimiento Universidad de Navarra, University of Navarra Follow-up) | General population | 18737/ CVD (fatal+ nonfatal): 127, non-fatal MI:62, stroke:33 | B, 10.3 | 38 | Age, sex, BMI, EI, alcohol, adherence to Mediterranean diet, smoking, education, FH of CHD, TG, HDL-C, HTN, DM, cancer, EE in LTPA and changes in PA in the 2th and 4th year follow-up, antiaggregant & anticoagulant treatment, with age and year of entering the cohort as stratification variables | LTPA | Inactive | 1.00 | Validated 17-item self-administered questionnaire previously | Medical record | Baseline |
|  |  |  |  |  |  |  |  |  | <6 Average METs | 0.47 (0.25-0.88) |  |  |  |
|  |  |  |  |  |  |  |  |  | ≥6 Average METs | 0.18 (0.05-0.62) |  |  |  |
| Hu, 2007(119) | Finland | 7 independent population surveys in 6 areas of Finland | General population | 21346/ CHD: 392 | B, 10 | 25- 64 | Study year, age, education, BMI, alcohol, FH of CHD, and FRS (total cholesterol, SBP, treatment for HTN, and cigarette), FH of CHD, OPA, commuting PA, LTPA | LTPA, Male | Low | 1.00 | self-administered questionnaire, not validated | Hospital record | Baseline |
|  |  |  |  |  |  |  |  |  | Moderate | 0.97 (0.86-1.10) |  |  |  |
|  |  |  |  |  |  |  |  |  | High | 0.66 (0.52-0.83) |  |  |  |
|  |  |  |  |  |  |  |  | LTPA, Female | Low | 1.00 |  |  |  |
|  |  |  |  |  |  |  |  |  | Moderate | 0.74 (0.60-0.92) |  |  |  |
|  |  |  |  |  |  |  |  |  | High | 0.54 (0.34-0.87) |  |  |  |
| Hummel, 2022(150) | Sweden | Swedish National March Cohort (SNMC) | General population | 31580/ Stroke + MI: 3500 | B, 17.9 | 48.2 | Age, smoking, alcohol, education, BMI, HTN, DM, and lipid disturbance | LTPA, total | High vs. low | 0.79 (0.66-0.94) | Validated questionnaire | National inpatient and outpatient register | Baseline |
|  |  |  |  |  |  |  |  | LTPA, male | High vs. low | 0.78 (0.62-0.98) |  |  |  |
|  |  |  |  |  |  |  |  | LTPA, female | High vs. low | 0.80 (0.61-1.04) |  |  |  |
| Jefferis, 2014(131) | UK | British Regional Heart Study | General population | 2641, CHD:236 | M, 11 | 40- 59 | Age, region, alcohol, smoking, plasma vitamin C, social class, total and HDL-c, TG, SBP, WC, forced expiratory volume in 1 second, GFR, depression, DM, CRP, von Willebrand factor, D-dimer, N-terminal pro-brain natriuretic peptide | Regular walking or cycling, recreational PA, sport (vigorous activity) | None | 1.00 | A PA score was derived from questionnaire (validated in relation to  heart rate and FEV1) | National health services central registers | Repeated |
|  |  |  |  |  |  |  |  |  | Occasional | 0.53 (0.35-0.81) |  |  |  |
|  |  |  |  |  |  |  |  |  | Light | 0.50 (0.32-0.78) |  |  |  |
|  |  |  |  |  |  |  |  |  | Moderate | 0.57 (0.35-0.92) |  |  |  |
|  |  |  |  |  |  |  |  |  | Moderately Vigorous and Vigorous | 0.51 (0.33-0.78) |  |  |  |
| Jensen, 2008(179) | Denmark | Danish Diet, Cancer and Health  study | General population | 25792, acute coronary syndrome: 858 | M, 7.7 | 50-64 | Age, PA, smoking, education, alcohol, postmenopausal status, hormone replacement therapy, self-reported physician-diagnosed HTN, hypercholesterolemia, DM | LTPA, male | <1 h/wk | 1.00 | Non-validated questionnaire | Hospital records | Baseline |
|  |  |  |  |  |  |  |  |  | 1- 3.5 h/wk | 0.88 (0.74-1.04) |  |  |  |
|  |  |  |  |  |  |  |  |  | ≥ 3.5 h/wk | 0.92 (0.77-1.10) |  |  |  |
|  |  |  |  |  |  |  |  | LTPA, female | <1 h/wk | 1.00 |  |  |  |
|  |  |  |  |  |  |  |  |  | 1- 3.5 h/wk | 0.67 (0.50-0.90) |  |  |  |
|  |  |  |  |  |  |  |  |  | ≥ 3.5 h/wk | 0.69 (0.50-0.96) |  |  |  |
| Kaprio, 2000(143) | Finland | Finnish Twin Cohort | Twin | 8205/ CHD: 311 | M, 6 | 25–69 | Age, BMI, smoking, HTN and DM | LTPA | Sedentary | 1.00 | Question  (unclear validation) | Hospital record | Baseline |
|  |  |  |  |  |  |  |  |  | Occasional exercisers | 0.84 (0.70-1.01) |  |  |  |
|  |  |  |  |  |  |  |  |  | Conditioning exercisers | 0.68 (0.50-0.92) |  |  |  |
| Kim 2019(180) | Korea | Korean NHIS—Health Screening (NHIS-HEALS) | General population | 257854/ MI: 3047 | B, 13 | 50.7 | Age, sex, BMI, BM, SBP, FBS, total cholesterol, FH of heart disease, stroke or HTN, smoking, alcohol | LTPA | None | 1.00 | IPAQ (non-validated) | presence of one hospitalization or two outpatient visits (validated) | Repeat |
|  |  |  |  |  |  |  |  |  | 1 – 2 times/wk | 0.87 (0.80-0.95) |  |  |  |
|  |  |  |  |  |  |  |  |  | 3- 4 times/wk | 0.79 (0.70-0.90) |  |  |  |
|  |  |  |  |  |  |  |  |  | 5- 6 times/wk | 0.98 (0.79-1.22) |  |  |  |
|  |  |  |  |  |  |  |  |  | Almost every day | 0.94 (0.82-1.09) |  |  |  |
| Koolhaas, 2016(181) | the Netherlands | The Rotterdam Study | Subjects aged ≥55 years in the municipality of Rotterdam | 5901/ CHD: 642 | B, 15 (median, 10.3) | aged >55 (median, 67 yr) | age sex, all other PA types, smoking, alcohol, diet, education, BMI, total and HDL cholesterol, DM, lipid-reducing agents, SBP, HTN | Walking | 9 MET-h/wk | 1.00 | adapted version of the Zutphen Physical Activity Questionnaire (validated) | Medical record | Baseline |
|  |  |  |  |  |  |  |  |  | 21 MET-h/wk | 1.16 (0.96-1.40) |  |  |  |
|  |  |  |  |  |  |  |  |  | 49.5 MET-h/wk | 0.97 (0.79-1.19) |  |  |  |
|  |  |  |  |  |  |  |  | Cycling | 0 MET-h/wk |  |  |  |  |
|  |  |  |  |  |  |  |  |  | 6 MET-h/wk | 0.80 (0.65-0.97) |  |  |  |
|  |  |  |  |  |  |  |  |  | 24 MET-h/wk | 0.76 (0.61-0.95) |  |  |  |
|  |  |  |  |  |  |  |  | Gardening | 9 MET-h/wk | 1.00 |  |  |  |
|  |  |  |  |  |  |  |  |  | 21 MET-h/wk | 0.90 (0.74-1.10) |  |  |  |
|  |  |  |  |  |  |  |  |  | 49.5 MET-h/wk | 0.99 (0.79-1.24) |  |  |  |
|  |  |  |  |  |  |  |  | Sport | 9 MET-h/wk | 1.00 |  |  |  |
|  |  |  |  |  |  |  |  |  | 21 MET-h/wk | 0.84 (0.67-1.05) |  |  |  |
|  |  |  |  |  |  |  |  |  | 49.5 MET-h/wk | 0.98 (0.79-1.23) |  |  |  |
| Lakka, 1994(135) | Finland | Kuopio Ischemic Heart Disease Risk Factor Study (KIHD) | General population | 1166/ MI: 42 | M, 4.9 | 42- 60 | Age, year of examination | LTPA | >2.2 h/wk vs. <0.7 h/wk | 0.31 (0.12-0.85) | Modified Minnesota LTPA questionnaire | Medical record | Baseline |
| Lacey, 2015(169) | Australia | The Health in Men Study | General population without prior vascular disease | 7564/ major CVE: 1557, IHD: 833, stroke: 551 | M, 11 | 72 | age at risk, education, smoking, BMI | Recreational PA | 0 Met-h/wk | 1 | Questionnaire (NM for validation) | Hospital record | Baseline |
|  |  |  |  |  |  |  |  |  | 1.14 Met-h/wk | 0.77 (0.65-0.91) |  |  |  |
|  |  |  |  |  |  |  |  |  | 15-24 Met-h/wk | 0.82 (0.70-0.96) |  |  |  |
|  |  |  |  |  |  |  |  |  | 25-39 Met-h/wk | 0.87 (0.74-1.02) |  |  |  |
|  |  |  |  |  |  |  |  |  | ≥ 40 Met-h/wk | 0.82 (0.70-0.95) |  |  |  |
| Merry, 2011(141) | Netherlands | CAREMA study | General population | 63444/ 472, Acute MI | B, 11.1 | 20- 59 | Age, sex, baseline cohort (PPHVZ or MORGEN), smoking, alcohol, total and HDL cholesterol, DM, SBP, and BMI | LTPA, male | Moderate to heavy PA vs. none to light PA | 1.03 (0.81-1.31) | Self-questionnaire  (unclear validation) | Cardiologic information system | Baseline |
|  |  |  |  |  |  |  |  | LTPA, female | Moderate to heavy PA vs. none to light PA | 1.81 (1.15-2.87) |  |  |  |
| Mu, 2021(171) | China | Dongfeng‑Tongji cohort | General population | 26584/ CHD: 4,659 | B, 5 | 63.3 | Age, sex, education, smoking, alcohol, consumption of meat, vegetables, and fruit, HTN, hyperlipidemia, DM, BMI, sedentary time, and FH of CVD | LTPA | < 7.5 Met-h/wk |  | Questionnaire (NM about validation) | Hospital record | Baseline |
|  |  |  |  |  |  |  |  |  | 7.5 to < 22.5 Met-h/wk | 0.97 (0.89-1.05) |  |  |  |
|  |  |  |  |  |  |  |  |  | 22.5 to < 37.5 Met-h/wk | 0.86 (0.77-0.96) |  |  |  |
|  |  |  |  |  |  |  |  |  | ≥ 37.5 Met-h/wk | 0.86 (0.77-0.97) |  |  |  |
| Patel, 2013(98) | USA | Cardiovascular Health Study (CHS) | aged 65 years and older | 5503 / Acute MI: ; Stroke: 1137 | B, 13 | 73 | age, sex, race, education, income, alcohol, smoking, BMI, CAD, AMI, HTN, DM, stroke, atrial fibrillation, LVH, left ventricular systolic dysfunction, SBP, DBP, depression, mental state, serum cholesterol, albumin, creatinine, and CRP | LTPA | 0 MET-mins/wk | 1.00 | modified Minnesota Leisure Time Activities questionnaire (validate) | self-reports of physician-diagnosed, which was then confirmed by review of  medical records | Baseline |
|  |  |  |  |  |  |  |  |  | 1–499 MET-mins/wk | 0.68 (0.51-0.91) |  |  |  |
|  |  |  |  |  |  |  |  |  | 500–999 MET-mins/wk | 0.65 (0.47-0.89) |  |  |  |
|  |  |  |  |  |  |  |  |  | ≥1000 MET-mins/wk | 0.68 (0.51-0.91) |  |  |  |
| Petersen, 2012(140) | Denmark | Danish National Health Interview Surveys | General population | 10689/MI:1384, IHD: 2824 | B, 13 | 16- 85 | Age, education, smoking, alcohol consumption, BMI, HTN, and self-perceived stress | LTPA, male | Moderate/vigorous vs. sedentary | 0.73 (0.56-0.95) | Saltin and Grimby questionnaire | National patient register | Baseline |
|  |  |  |  |  |  |  |  | LTPA, female | Moderate/vigorous vs. sedentary | 0.98 (0.61-1.58) |  |  |  |
| Raza, 2021(182) | Sweden | Västerbotten Intervention Programme (VIP) | General population | 34748, IHD: 1148 | B,12.4 | 45.8 | Age, sex, education, smoking, alcohol, occupation, neighborhood mean income, active commuting | LTPA  (exercise in training clothes) | Never | 1.00 | Questionnaire  (NM about validity) | National Patient Register and  the Cause of Death Register | Baseline |
|  |  |  |  |  |  |  |  |  | ≤Once/week | 1.03 (0.90-1.16) |  |  |  |
|  |  |  |  |  |  |  |  |  | ≥Twice/week | 0.76 (0.62-0.93) |  |  |  |
| Renninger, 2018(183) | Norwegian | Tromsø Study | General population | 16,572/ MI: 1613 | B, 34 | 20–54 | Age, sex, BMI/LTPA, daily smoking, SBP, DBP, total cholesterol, diabetes, HTN treatment | LTPA | High vs. low | 0.88 (0.77-1.01) | a validated  questionnaire  (Saltin and Grimby) | discharge diagnosis  register at the Hospital | Baseline |
| Rosolova, 1994(142) | Czech | Pilsen Longitudinal Study | Middle-Aged subjects | 3540/ CHD: 68 | M, 10.7 | 40- 50 | Age, smoking, SBP, DBP, serum cholesterol, BMI, FH, alcohol and coffee, education level | LTPA | High vs. low | 0.97 (0.69-1.37) | Questionnaire  (unclear validation) | Hospital records | Baseline |
| Sesso, 2000(184) | USA | Harvard Alumni Health Study | General population (middle-aged and older men) | 12516, CHD (Fatal + non-fatal): 2135  (1295  identified by questionnaire and 840 from  death certificates) | M, 20 | 39-88 (57.7) | age, BMI, alcohol, HTN, DM, smoking, early parental death | flights climbed, blocks walked, and sports played | <500 kcal/wk | 1.00 | A valid questionnaire | Self-report | Baseline |
|  |  |  |  |  |  |  |  |  | 500-1000 kcal/wk | 0.90 (0.79-1.03) |  |  |  |
|  |  |  |  |  |  |  |  |  | 1000- 2000 kcal/wk | 0.81 (0.71-0.92) |  |  |  |
|  |  |  |  |  |  |  |  |  | 2000 -3000 kcal/wk | 0.80 (0.69-0.93) |  |  |  |
|  |  |  |  |  |  |  |  |  | ≥ 3000 kcal/wk | 0.81 (0.71-0.94) |  |  |  |
| Strippoli, 2022(152) | Italy | Italian National Health Interview Surveys | General population | 40,220/ CHD: 1442 | B, 14 | 40–55 | Age, family type and macroeconomic region, each domain of PA separately, general health status, smoking, BMI, education, other domains of PA | LTPA, OPA | High vs. low | 0.93 (0.7- 0.15) | Non-valid questionnaire | linkage  with  hospital discharge records (Ministry of  Health) | Baseline |
| Sundquist, 2005(153) | Sweden | Swedish Annual Level-of-Living Survey (SALLS) | General population | 5196/ CHD (fatal+ non-fatal): 373 | B, 11.7 | 35–74 | sex, age, PA, income, smoking, BMI | LTPA | vigorously at least twice a week vs. none | 0.59 (0.37-0.95) | A valid questionnaire | first hospitalization for CHD event. Data were obtained from the Swedish National Hospital Discharge Register and the Cause-of-Death  Register | Baseline |
| Tanasescu, 2002(117) | USA | Health Professionals' Follow-up Study | Health Professionals | 44452/ fatal CHD+ non-fatal MI: 1700 | M, 10 | 40 -75 | Age, alcohol, smoking, FH of MI, vitamin E supplements, history of DM, HTN, and hypercholesterolemia at baseline, dietary intake of TFA, PUFAs, fiber, and folate, PA activity, BMI | LTPA | 0 6.32 MET-h/wk | 1.00 | Validated questionnaire | Self-reported MI were confirmed by a review of medical records based on WHO criteria | Repeated (every 2 years) |
|  |  |  |  |  |  |  |  |  | 6.33 – 14.49 MET-h/wk | 0.90 (0.78-1.04) |  |  |  |
|  |  |  |  |  |  |  |  |  | 14.5- 25.08 MET-h/wk | 0.88 (0.76-1.01) |  |  |  |
|  |  |  |  |  |  |  |  |  | 25.09- 41.98 MET-h/wk | 0.84 (0.72-0.98) |  |  |  |
|  |  |  |  |  |  |  |  |  | ≥41.99 MET-h/wk | 0.72 (0.61-0.85) |  |  |  |
| Wagner, 2002(139) | Northern Ireland and France | PRIME Study | Middle-aged  Men (50 to 59 years of age) | 9758/167 hard CHD, 154 | M, 5 | 50- 59 | Age, country, employment status, tobacco, alcohol, education, BMI | LTPA | Per 10 MET - h/week | 0.92 (0.86, 0.99) | MONICA Optional Study of Physical Activity Questionnaire, validated | practitioners’  notes | Repeated |
| Weinstein, 2008(105) | USA | Women’s Health Study | female health professionals | 38987, CHD: 948 | F, 10.9 | ≥45 | Age, randomized treatment assignments, parental history of MI, alcohol, smoking, hormone therapy, and dietary factors | LTPA | Active (≥1000 kcal/wk) vs. Inactive (<1000 kcal/wk) | 0.82 (0.70-0.96) | Validated self-reported questionnaire | Medical records | Repeated |
| Zhao, 2020(154) | China | Electronic health check-up database Xinzheng City | Elderly population | 32,942/ CVD: 6,857, stroke:2240, CHD: 4937 | B, 6.84 | ≥68 | Age, sex, current smoking, alcohol, BMI, medical history of T2DM, SBP, DBP, RHR, FPG, TC, TG and HDL-C | LTPA | Very highly active vs. inactive | 0.84 (0.66-1.06) | Questionnaire, NM about validation | data of the annual standard health check-up record  with digital linkage to the hospital dataset | Baseline |
| Abbreviations: AHEI, Alternate Healthy Eating Index; AMI, acute myocardial infarction; Atrial fibrillation, AF; B, both; BP, blood pressure; BMI, body mass index; CAD, coronary artery disease; CE, coronary events; CHD, coronary heart disease; CI, confidence interval; COPD, chronic obstructive pulmonary disease ; CRP, C-reactive protein; CVE, cardiovascular events; DBP, diastolic blood pressure; DM, diabetes mellitus; EE, energy expenditure; EI, energy intake; EPA, eicosapentaenoic acid; EPIC, European Prospective Investigation into Cancer and Nutrition; F, female; FBS, fasting blood sugar; FRS, Framingham risk score; FH, family history; GFR, glomerular filtration rate; HDL-C, high density lipoproteins cholesterol; HF, heart failure; HTN, hypertension; IHD, ischemic heart disease; LDL-C, low density lipoproteins cholesterol; LTPA, leisure time physical activity; LVH, left ventricular hypertrophy; M, Male; MONICA, Multinational Monitoring of Trends and Determinants in Cardiovascular Disease; NHIS, National Health Insurance Service; NM, not mentioned; NSAID, non-steroidal anti-inflammatory; OCP, oral contraceptive; OPA, occupational physical activity; PA, physical activity; PAD, peripheral artery disease; PAMELA, Pressioni Arteriose Monitorate Loro Associazioni; PUFA, polyunsaturated fatty acid; PY, person-years; RR, risk ratio; SEMM, Study of Employed in the Municipality of Milan, SBP, systolic blood pressure; SIMPLER, Swedish Infrastructure for Medical Population-based Life-course Environmental Research; T-C, total cholesterol; TFA, trans fatty acids; TG, triglyceride; TIA, transient ischemic attack ; WC, waist circumference; WHI-OS, Women’s Health Initiative (WHI) Observational Study; wk, week | | | | | | | | | | | | | |

**Supplementary Table 7. Characteristic of studies investigating the relationship between LTPA and stroke**

| **Author, year** | **Country** | **Study name** | **Population characteristics** | **Total participants, case (num)** | **Sex, Duration (Year)** | **Age (range or mean)** | **Adjustment factors** | **Type of PA** | **PA level** | **RR, 95% CI** | **Exposure assessment** | **Outcome assessment** | **PA timed assessed (Baseline, repeated)** |
| --- | --- | --- | --- | --- | --- | --- | --- | --- | --- | --- | --- | --- | --- |
| Ahmed 2020(130) | UK | British Regional Heart Study | General population | 7163, Stroke: 434 | B, 20 | 50 | Age, social class and alcohol intake | LTPA | High vs. low | 0.63 (0.45, 0.88) | Validated Questionnaire | Medical records | Baseline |
| Agnarsson, 1999(137) | Iceland | Reykjavı ´k Study | General population | 4484/ Stroke: 249 | M, 14.5 | 45-80 | Age and smoking | LTPA, total stroke | Yes vs. No | 0.69 (0.47–1.01) | Questionnaire  (non-validated) | Hospital records | Baseline |
|  |  |  |  |  |  |  |  | LTPA, Ischemic stroke | Yes vs. No | 0.62 (0.40–0.97) |  |  |  |
| Amidei 2022(155) | Italy | Progetto Veneto Anziani | Elderly population | 2321, stroke:420 | B, 7 | > 65 | Sex, education, household members, smoking, alcohol, a number of chronic diseases (cancer, COPD, asthma, DM, CKD, Parkinson’s disease), CVD (CHD, HF, stroke, angina, TIA, AF, HTN and PAD with the exclusion of interested outcome) at baseline, year of birth | Moderate to vigorous LTPA, male | Active vs. low inactive | 1.02 (0.69, 1.52) | Validated questionnaire | Electronic health records | Repeated |
|  |  |  |  |  |  |  |  | Moderate to vigorous LTPA, female | Active vs. low inactive | 1.14 (0.87, 1.48) |  |  |  |
| Armstrong 2015(156) | UK | Million Women Study | General population | 1119239/ Cerebrovascular Disease:19649 | F, 9 | 50- 64 | BMI-by-age, smoking-by-age, alcohol-by-age, and stratified by socioeconomic status and region | Total LTPA, Cerebrovascular Disease | Rarely/never  (inactive) | 1.00 | Non-validated questionnaire | Electronic health records | Repeated |
|  |  |  |  |  |  |  |  |  | At most once  per wk | 0.87 (0.84, 0.90) |  |  |  |
|  |  |  |  |  |  |  |  |  | 2–3 times/wk | 0.80 (0.77–0.83) |  |  |  |
|  |  |  |  |  |  |  |  |  | 4–6 times/wk | 0.83 (0.79–0.88) |  |  |  |
|  |  |  |  |  |  |  |  |  | Daily | 0.88 (0.86–0.91) |  |  |  |
|  |  |  |  |  |  |  |  | Total LTPA, Subarachnoid Hemorrhage | Daily activity vs. Rarely/ never | 0.87 (0.78–0.97) |  |  |  |
|  |  |  |  |  |  |  |  | Total LTPA, Intracerebral Hemorrhage | Daily activity vs. Rarely/ never | 0.98 (0.89–1.09) |  |  |  |
|  |  |  |  |  |  |  |  | Total LTPA, Cerebral Infarction | Daily activity vs. Rarely/ never | 0.91 (0.86–0.97) |  |  |  |
|  |  |  |  |  |  |  |  | Strenuous LTPA, Cerebrovascular Disease | Daily activity vs. Rarely/ never | 0.96 (0.89–1.04) |  |  |  |
| Barengo, 2016(157) | Finland | National FINRISK | Elderly | 2456/ stroke: 226 | B, 11.8 | 65- 74 | Age, sex, area, BMI, serum cholesterol, SBP, smoking, education, marital status, inability to perform PA | LTPA, all | Low | 1.00 | Self-administered questionnaire (non-validated) | National hospital discharge register | Baseline |
|  |  |  |  |  |  |  |  |  | Moderate | 0.60 (0.43, 0.84) |  |  |  |
|  |  |  |  |  |  |  |  |  | High | 0.41 (0.24, 0.69) |  |  |  |
|  |  |  |  |  |  |  |  | LTPA, men | Low | 1.00 |  |  |  |
|  |  |  |  |  |  |  |  |  | Moderate | 0.60 (0.39, 0.93) |  |  |  |
|  |  |  |  |  |  |  |  |  | High | 0.38 (0.20, 0.73) |  |  |  |
|  |  |  |  |  |  |  |  | LTPA, women | Low | 1.00 |  |  |  |
|  |  |  |  |  |  |  |  |  | Moderate | 0.61 (0.36, 1.03) |  |  |  |
|  |  |  |  |  |  |  |  |  | High | 0.48 (0.19, 1.22) |  |  |  |
| Blomstrand, 2014(121) | Sweden | Prospective Population Study of Women in Gothenburg | General population | 1462/ Ischemic stroke and Hemorrhagic stroke: 337 | F, 33 | 38–60 | Age, HTN, BMI, smoking, physical inactivity, cholesterol, TG, mental stress and educational level | LTPA, Total | Physical inactivity | 1.38 (1.04 to 1.83) | NM about validation of Questionnaire | Hospital records | Baseline |
|  |  |  |  |  |  |  |  | LTPA, Ischemic | Physical Inactivity | 1.22 (0.88 to 1.70) |  |  |  |
|  |  |  |  |  |  |  |  | LTPA, Hemorrhagic | Physical inactivity | 2.18 (1.04 to 4.58) |  |  |  |
| Chomistek, 2013(108) | Us | WHI-OS | postmenopausal women | 71,018 / stroke: 2050 | F, 12.2 | 50 – 79 | The multivariable model was stratified by age and includes sedentary time and PA simultaneously, race, education, income, marital status, smoking, FH of MI, depression, alcohol, sleep, EI, saturated fat, fiber, HTN, DM, high cholesterol, BMI | LTPA | ≤ 1.7 MET-h/wk | 1.00 | validated self-reported questionnaire | Self-report + Medicare and Medicaid Services data which were available only for some participants | Repeated |
|  |  |  |  |  |  |  |  |  | 1.8 – 8.3  MET-h/wk | 1.08 (0.95,1.23) |  |  |  |
|  |  |  |  |  |  |  |  |  | 8.4 – 20  MET-h/wk | 1.19 (1.04,1.36) |  |  |  |
|  |  |  |  |  |  |  |  |  | > 20  MET-h/wk | 1.30 (1.13, 1.50) |  |  |  |
| Chomistek, 2018(104) | USA | Women’s Health Study | Female health professionals | 27536/ stroke: 650 | F, 19.1 | ≥45 | Age, randomized treatment assignment, smoking, alcohol, saturated fat, fiber, fruits, vegetables; menopausal status; postmenopausal hormone use, FH of MI <60 y, T-C and HDL-C, SBP, DM, | LTPA | 0-199 kcal/wk | 1.00 | validated self-reported questionnaire | Medical records | Repeated |
|  |  |  |  |  |  |  |  |  | 200–599  kcal/wk | 0.93 (0.75–1.15) |  |  |  |
|  |  |  |  |  |  |  |  |  | 600-1499 kcal/wk | 0.81 (0.66–1.00) |  |  |  |
|  |  |  |  |  |  |  |  |  | ≥1500  kcal/wk | 0.72 (0.57–0.90) |  |  |  |
| Cuthbertson, 2019(102) | USA | Atherosclerosis Risk in Communities (ARIC) | General population | 13534/ stroke: 1144 | B, 27.2 | 45- 64 (54) | Age, sex, race by ARIC center, education, smoking, and alcohol intake | LTPA | No LTPA | 1.00 | Baecke questionnaire, validated | Diagnostic  algorithm and hospital records | Repeated |
|  |  |  |  |  |  |  |  |  | <13.2 MET h/wk | 0.86 (0.74–1.00) |  |  |  |
|  |  |  |  |  |  |  |  |  | ≥13.2 MET h/wk | 0.80 (0.68–0.92) |  |  |  |
| Djousse, 2021(23) | USA | Million Veteran Program (MVP) | Veteran | 315,119/ hemorrhagic stroke: 981  Ischemic stroke: 10404 | B, 3.3 | 64.6 | Age, sex, race, education, BMI, smoking, alcohol, DASH score | LTPA | 0 | 1.00 | NM about validation of questionnaire | Medical records | Baseline |
|  |  |  |  |  |  |  |  |  | 0.1–1.24  MET-h/wk | 0.83 (0.77, 0.91) |  |  |  |
|  |  |  |  |  |  |  |  |  | 1.25–3.80 MET-h/wk | 0.76 (0.7, 0.83) |  |  |  |
|  |  |  |  |  |  |  |  |  | 3.81–6.60 MET-h/wk | 0.76 (0.69, 0.83) |  |  |  |
|  |  |  |  |  |  |  |  |  | 6.61–30 MET-h/wk | 0.68 (0.63, 0.74) |  |  |  |
| Elliott, 2020(111) | USA | NHS | Nurse | 104990/ stroke: 2,848 | F, 20 | 30–55 | Age, race, cancer, FH of MI, smoking, AHEI score, multivitamin, alcohol, census tract, income, home value, occupation of father and mother, husband’s education, registered nursing degree, marital status, retirement status | LTPA | <3.7 MET-h/wk | 1.00 | Validated questionnaire | Medical records | Repeated |
|  |  |  |  |  |  |  |  |  | 3.7–10.8 MET-h/wk | 0.73(0.66,0.80) |  |  |  |
|  |  |  |  |  |  |  |  |  | 10.9–24.3 MET-h/wk | 0.71(0.64,0.78) |  |  |  |
|  |  |  |  |  |  |  |  |  | ≥24.4 MET-h/wk | 0.58(0.52,0.65) |  |  |  |
|  |  |  |  |  |  |  |  |  | <1.4 MET-h/wk | 1.00 |  |  |  |
|  |  |  |  |  |  |  |  |  | 1.4 – 6.2 MET-h/wk | 1.11(0.87,1.42) |  |  |  |
|  |  |  |  |  |  |  |  |  | 6.3- 13.4  MET-h/wk | 1.03(0.79,1.34) |  |  |  |
|  |  |  |  |  |  |  |  |  | ≥13.5 MET-h/wk | 0.86(0.66,1.13) |  |  |  |
| Ford, 2009(163) | German | EPIC–Potsdam  study | General population | 23153, stroke: 195 | B, 7.8 | 49.3 (35-65) | Age, sex, education, occupational status, BMI, diet, smoking, | sports and bicycle riding | Yes vs. no | 0.74 (0.46, 1.03) | NM about validation | Medical records | Baseline |
| Gillum, 1996(148) | USA | NHANES I | Black participants | 771/ Stroke: 104 | B, 11.6 | 45-74 | Age, sex, smoking, SBP, serum total cholesterol, history of DM, hemoglobin, BMI, history of heart disease, education | Recreational PA, Black | Low vs. high | 1.33 (0.67-2.63) | Questions on habitual physical activity were asked (unclear validation) | Hospital records | Baseline |
|  |  |  |  |  |  |  |  | Recreational, white women, 45-64 y | Low vs. high | 3.13 (0.95-10.32) |  |  |  |
|  |  |  |  |  |  |  |  | Recreational, white women, 65-74 y | Low vs. high | 1.55 (0.95-2.53) |  |  |  |
|  |  |  |  |  |  |  |  | Recreational, white men, 45 – 64 y | Low vs. high | 1.24 (0.63-2.41) |  |  |  |
|  |  |  |  |  |  |  |  | Recreational, white men, 65-74 y | Low vs. high | 1.29 (0.88-1.88) |  |  |  |
| Hall, 2019(185) | USA | Sister study | working women | 31270 / transient ischemic attack + Stroke: 715 | F, 5.7 | 35–74 | Age, LTPA, alcohol, smoking, BMI, discrimination at work, night work, heart rate and pulse pressure (SBP minus DBP) | LTPA, Total stroke | ≥6 MET at ≥75 mins/wk vs. no LTPA | 0.64 (0.44–0.94) | computer-assisted telephone interviews (non-validated) | Self-reported doctor’s diagnoses | Baseline |
|  |  |  |  |  |  |  |  | LTPA, TIA | ≥6 MET at ≥75 mins/wk vs. no LTPA | 0.69 (0.42–1.13) |  |  |  |
| Harmsen, 2006(132) | Sweden | Multifactor Primary Prevention Study | Middle-Aged subjects | 7457/ stroke: 1019 | M, 28 | 47- 55 | Age, Social class, BMI, Psychological stress, history of chest pain, DM, and stroke, smoking, FH of CE, antihypertensive medication, SBP. | LTPA | Low physical activity vs. high | 1.11 (0.90–1.36) | Questionnaire (validated unclear) | Register based | Baseline |
| Hidalgo-Santamaria, 2018(127) | Spain | SUN Project (Seguimiento Universidad de Navarra, University of Navarra Follow-up) | General population | 18737/ CVD (fatal+ nonfatal): 127, non-fatal MI:62, stroke:33 | B, 10.3 | 38 | Age, sex, BMI, EI, alcohol, adherence to Mediterranean diet, smoking, education, FH of CHD, TG, HDL-C, HTN, DM, cancer, EE in LTPA and changes in PA in the 2th and 4th year follow-up, antiaggregant & anticoagulant treatment, with age and year of entering the cohort as stratification variables | LTPA | Inactive | 1.00 | Validated 17-item self-administered questionnaire previously | Medical record | Baseline |
|  |  |  |  |  |  |  |  |  | <6 Average METs | 1.22 (0.44-3.43) |  |  |  |
|  |  |  |  |  |  |  |  |  | ≥6 Average METs | 0.90 (0.17-4.59) |  |  |  |
| Hu, 2005(120) | Finland | 7 independent population surveys in 6 areas of Finland | General population | 47721/ Stroke: 2863 | B, 19 | 25- 64 | Age, area, and study year, MI, SBP, cholesterol, education, smoking, alcohol, and DM, other ypes of PA | LTPA, total | High vs. low | 0.74 (0.64–0.85) | Self-reported questionnaire, not validated | Hospital record | Baseline |
|  |  |  |  |  |  |  |  | LTPA, Males | High vs. low | 0.72 (0.60–0.87) |  |  |  |
|  |  |  |  |  |  |  |  | LTPA, females | High vs. low | 0.77 (0.62–0.97) |  |  |  |
| Huerta, 2013(186) | Spain | EPIC-Spain | General population | 32992/ transient ischemic attack: 112, Stroke:271 | B, 12.3 | 29-69 | Age, center, educational level, self-reported HTN or hyperlipidemia, DM, smoking, age at start smoking, alcohol, EI, BMI, WC, consumption of protein, lipids, vegetables, red meat, and fish, PA. | Recreational PA, stroke | ≤19.50 MET-h/wk | 1.00 | EPIC-PAQ included 9 specific questions | Self-report and computerized primary care registers | Baseline |
|  |  |  |  |  |  |  |  |  | 19.51–33.75 MET-h/wk | 0.76 (0.55–1.06) |  |  |  |
|  |  |  |  |  |  |  |  |  | 33.76–54.75 MET-h/wk | 0.90 (0.64–1.27) |  |  |  |
|  |  |  |  |  |  |  |  |  | >54.75 MET-h/wk | 1.12 (0.78–1.60) |  |  |  |
|  |  |  |  |  |  |  |  | Recreational PA, TIA | ≤19.50 MET-h/wk | 1.00 |  |  |  |
|  |  |  |  |  |  |  |  |  | 19.51–33.75 MET-h/wk | 0.96 (0.58–1.56) |  |  |  |
|  |  |  |  |  |  |  |  |  | 33.76–54.75 MET-h/wk | 0.72 (0.42–1.25) |  |  |  |
|  |  |  |  |  |  |  |  |  | >54.75 MET-h/wk | 0.86 (0.49–1.53) |  |  |  |
| Hummel, 2022(150) | Sweden | Swedish National March Cohort (SNMC) | General population | 31580/ Stroke + MI: 3500 | B, 17.9 | 48.2 | Age, smoking, alcohol, education, BMI, HTN, DM, and lipid disturbance | LTPA, both sex, total stroke | High vs. low | 0.86 (0.73–1.00) | Validated questionnaire | National inpatient and outpatient register | Baseline |
|  |  |  |  |  |  |  |  | LTPA, male, total stroke | High vs. low | 0.78 (0.61–0.99) |  |  |  |
|  |  |  |  |  |  |  |  | LTPA, female, total stroke | High vs. low | 0.91 (0.74–1.13) |  |  |  |
|  |  |  |  |  |  |  |  | LTPA, both sex, ischemic stroke | High vs. low | 0.90 (0.74–1.09) |  |  |  |
|  |  |  |  |  |  |  |  | LTPA, male, ischemic stroke | High vs. low | 0.75 (0.56–1.00) |  |  |  |
|  |  |  |  |  |  |  |  | LTPA, female, ischemic stroke | High vs. low | 1.03 (0.79–1.33) |  |  |  |
|  |  |  |  |  |  |  |  | LTPA, both sex, hemorrhagic stroke | High vs. low | 0.75 (0.52–1.08) |  |  |  |
|  |  |  |  |  |  |  |  | LTPA, male, hemorrhagic stroke | High vs. low | 0.87 (0.48–1.58) |  |  |  |
|  |  |  |  |  |  |  |  | LTPA, female, hemorrhagic stroke | High vs. low | 0.70 (0.44–1.09) |  |  |  |
| Jeong, 2017(187) | Korea | Korean NHIS | General population | 336326/ stroke: 2213 | B, 3.6 | > 18 | Age, sex, socioeconomic status, current smoking, drinking, BMI, HTN, DM, hypercholesterolemia, SBP, FBS, and total cholesterol | moderate- to vigorous-intensity PA, total stroke | None vs. any moderate-to-vigorous PA | 1.24 (1.14–1.36) | IPAQ (non-validated) | presence of one hospitalization or two outpatient visits (validated) | Baseline |
|  |  |  |  |  |  |  |  | moderate- to vigorous-intensity PA, ischemic Stroke | None vs. any moderate-to-vigorous PA | 1.26 (1.14–1.39) |  |  |  |
|  |  |  |  |  |  |  |  | moderate- to vigorous-intensity PA, intracerebral hemorrhage | None vs. any moderate-to-vigorous PA | 1.18 (0.94–1.47) |  |  |  |
| Johansson, 2021(123) | Sweden | Malmö Diet and Cancer study | General population | 26549/ Ischemic Stroke: 2330 | B, 21.2 | 46–73 | Age, sex, education, FH of stroke, HTN, dyslipidemia, atherosclerotic disease, DM, smoking, diet quality, PA, BMI, alcohol | LTPA | High vs. low | 0.89 (0.80–0.98) | Questionnaire (validate) | National patient register | Baseline |
| Johansson, 2022(126) |  |  |  |  |  |  |  | LTPA | Per SD increase (20 MET-h/wk) | 0.96 (0.92-1.01) |  |  |  |
| Lacey, 2015(169) | Australia | The Health in Men Study | General population without prior vascular disease | 7564/ major CVE: 1557, IHD: 833, stroke: 551 | M, 11 | 72 | age at risk, education, smoking, BMI | Recreational PA | 0 Met-h/wk |  | Questionnaire (NM for validation) | Hospital record | Baseline |
|  |  |  |  |  |  |  |  |  | 1.14 Met-h/wk | 1.10 (0.91-1.32) |  |  |  |
|  |  |  |  |  |  |  |  |  | 15-24 Met-h/wk | 0.87 (0.71-1.06) |  |  |  |
|  |  |  |  |  |  |  |  |  | 25-39 Met-h/wk | 0.83 (0.68-1.03) |  |  |  |
|  |  |  |  |  |  |  |  |  | ≥ 40 Met-h/wk | 0.92 (0.76-1.11) |  |  |  |
| Larsson, 2014(113) | Sweden | Swedish Mammography Cohort | General population | 31696, Stroke: 1554 | F, 10.4 | >18 | Age, education, aspirin, history of DM, diagnosis of AF, FH of MI <60 years, total EI, Non- Recommended Food Score, alcohol, BMI, smoking | walking/bicycling and exercise, total stroke | ≥40 min/d walking/bicycling and ≥1 h/wk  exercise vs less | 0.94 (0.84–1.05) | Questionnaire (NM for validation) | National patient record | Baseline |
|  |  |  |  |  |  |  |  | walking/bicycling and exercise, Cerebral infarction | ≥40 min/d walking/bicycling and ≥1 h/wk  exercise vs less | 0.91 (0.81–1.04) |  |  |  |
|  |  |  |  |  |  |  |  | walking/bicycling and exercise, Hemorrhagic stroke | ≥40 min/d walking/bicycling and ≥1 h/wk  exercise vs less | 1.04 (0.80–1.36) |  |  |  |
| Lee, 1998(188) | USA | The Harvard Alumni Health Study | University students without CVD and cancer at baseline (1916- 1950 | 11130/Stroke: 378 | M, 13 | 58 | Age, smoking, alcohol consumption, and early parental death | Sport & recreation PA+ blocks walked & stairs  climbed daily, Activities at <6 METs) | None | 1.00 | Valid questionnaire | Self-reported, physician-diagnosed | Baseline |
|  |  |  |  |  |  |  |  |  | 1 -250 kcal/wk | 0.90 (0.64–1.26) |  |  |  |
|  |  |  |  |  |  |  |  |  | 250-600 kcal/wk | 0.69 (0.47–1.01) |  |  |  |
|  |  |  |  |  |  |  |  |  | 600 -1400 kcal/wk | 0.86 (0.61–1.21) |  |  |  |
|  |  |  |  |  |  |  |  |  | >1400 kcal/wk | 0.86 (0.63–1.19) |  |  |  |
|  |  |  |  |  |  |  |  | Activities at ≥6 METs, kcal/wk | None | 1.00 |  |  |  |
|  |  |  |  |  |  |  |  |  | 1 -250 kcal/wk | 0.90 (0.67–1.22) |  |  |  |
|  |  |  |  |  |  |  |  |  | 250 -600 kcal/wk | 0.67 (0.45–1.02) |  |  |  |
|  |  |  |  |  |  |  |  |  | 600 -1400 kcal/wk | 0.82 (0.56–1.18) |  |  |  |
|  |  |  |  |  |  |  |  |  | >1400 kcal/wk | 1.03 (0.73–1.44) |  |  |  |
| Mu, 2021(171) | China | Dongfeng‑Tongji cohort | General population | 26584/ Stroke: 1045 | B, 5 | 63.3 | Age, sex, education, smoking, alcohol, consumption of meat, vegetables, and fruit, HTN, hyperlipidemia, DM, BMI, total sedentary time, and FH of CVD | LTPA | < 7.5 Met-h/wk | 1 | Questionnaire (NM about validatio) | Hospital record | Baseline |
|  |  |  |  |  |  |  |  |  | 7.5 to < 22.5 Met-h/wk | 0.81 (0.68, 0.97) |  |  |  |
|  |  |  |  |  |  |  |  |  | 22.5 to < 37.5  Met-h/wk | 0.72 (0.58, 0.90) |  |  |  |
|  |  |  |  |  |  |  |  |  | ≥ 37.5 Met-h/wk | 0.65 (0.50, 0.83) |  |  |  |
| Patel, 2013(98) | USA | Cardiovascular Health Study (CHS) | aged 65 years and older | 5503 / HF, Acute MI, Angina pectoris, Stroke: 1137 | B, 13 | 73 | age, sex, race, education, income, alcohol, smoking, BMI, CAD, AMI, HTN, DM, stroke, AF, LVH, left ventricular systolic dysfunction, SBP, DBP, depression, mental state, serum cholestero, albumin, creatinine, and CRP | LTPA | 0 MET-min/wk | 1.00 | modified Minnesota Leisure Time Activities questionnaire (validate) | self-reports of physician-diagnosed, which was then confirmed by review of  medical records | Baseline |
|  |  |  |  |  |  |  |  |  | 1–499 MET-min/wk | 0.76 (0.59–0.99) |  |  |  |
|  |  |  |  |  |  |  |  |  | 500–999 MET-min/wk | 0.68 (0.52–0.91) |  |  |  |
|  |  |  |  |  |  |  |  |  | ≥1000 MET-min/wk | 0.75 (0.58–0.96) |  |  |  |
| Rist 2021(189) | USA | Physician's Health Study | Physicians | 21794, Stroke: 2139 | M, 20.2 | 53.8 | Age, smoking, alcohol, parental history of MI before age 60, and randomized treatment assignments | LTPA (vigorous activity), Total stroke | <1 time/wk | 1.00 | Non-validated questionnaire | Medical records | Repeated |
|  |  |  |  |  |  |  |  |  | 1 time/wk | 0.94 (0.81–1.10) |  |  |  |
|  |  |  |  |  |  |  |  |  | 2–4 times/wk | 0.86 (0.75–0.98) |  |  |  |
|  |  |  |  |  |  |  |  |  | ≥5 times/wk | 0.88 (0.75–1.03) |  |  |  |
|  |  |  |  |  |  |  |  | LTPA, Ischemic Stroke | <1 time/wk | 1.00 |  |  |  |
|  |  |  |  |  |  |  |  |  | 1 time/wk | 0.96 (0.81–1.14) |  |  |  |
|  |  |  |  |  |  |  |  |  | 2–4 times/wk | 0.89 (0.77–1.03) |  |  |  |
|  |  |  |  |  |  |  |  |  | ≥5 times/wk | 0.89 (0.74–1.06) |  |  |  |
|  |  |  |  |  |  |  |  | LTPA, Hemorrhagic Stroke | <1 time/wk | 1.00 |  |  |  |
|  |  |  |  |  |  |  |  |  | 1 time/wk | 0.90 (0.61–1.31) |  |  |  |
|  |  |  |  |  |  |  |  |  | 2–4 times/wk | 0.72 (0.52–1.01) |  |  |  |
|  |  |  |  |  |  |  |  |  | ≥5 times/wk | 0.88 (0.59–1.30) |  |  |  |
|  |  |  |  |  |  |  |  | LTPA, TIA | <1 time/wk | 1.00 |  |  |  |
|  |  |  |  |  |  |  |  |  | 1 time/wk | 0.99 (0.81–1.22) |  |  |  |
|  |  |  |  |  |  |  |  |  | 2–4 times/wk | 0.86 (0.72–1.02) |  |  |  |
|  |  |  |  |  |  |  |  |  | ≥5 times/wk | 0.66 (0.52–0.84) |  |  |  |
| Sieverdes, 2015(151) | USA | Aerobics Center Longitudinal Study | General population | 45689, Stroke (non-fatal): 465 | M, 15.5 | 18-100 | Age, examination year, survey response pattern for nonfatal and total stroke, smoking, alcohol, FH of CVD,  hypercholesterolemia, HTN, DM, cardiorespiratory fitness | LTPA | Sport vs. sedentary | 0.78 (0.57–1.07) | validated questionnaire | Responses to mail-back health surveys | Baseline |
| Willey, 2017(116) | USA | NOMAS: Northern Manhattan Stroke Study | stroke-free multi-ethnic community, | 3298/ stroke: 391 | B, 14 | ≥ 40, 69 | sex, race-ethnicity, education, insurance status, alcohol, HDL-C, LDL-C, tobacco, DM, HTN, AF, IHD, CHF, GFR | LTPA | LTP inactivity (vs. Any LTPA) | 0.89 (0.72–1.11) | A valid and reliable questionnaire | Physician diagnosis | Baseline |
| Willey, 2017(136) | USA | CTS: California Teachers Study | current and retired female teachers of California | 61256/ stroke: 987 | F, 10 | 53±14 | Age, race, socioeconomic status, tobacco, alcohol, BMI, HTN, DM, hyperlipidemia | Moderate to vigorous LTPA, total stroke | Either recommendation met vs. neither recommendation met | 0.83 (0.73–0.94) | A validated  questionnaire adapted from the NHISNCHS | Hospital records | repeated, 2 times |
|  |  |  |  |  |  |  |  | Moderate to vigorous LTPA, Ischemic Stroke | Either recommendation met vs. neither recommendation met | 0.82 (0.70–0.96) |  |  |  |
|  |  |  |  |  |  |  |  | Moderate to vigorous LTPA, Hemorrhagic Stroke | Either recommendation met vs. neither recommendation met | 0.86 (0.65–1.13) |  |  |  |
|  |  |  |  |  |  |  |  | Moderate LTPA, total Stroke | >150 min/wk vs. ≤150 min/wk | 0.84 (0.73–0.96) |  |  |  |
|  |  |  |  |  |  |  |  | Moderate LTPA, Ischemic stroke | >150 min/wk vs. ≤150 min/wk | 0.82 (0.70–0.96) |  |  |  |
|  |  |  |  |  |  |  |  | Moderate LTPA, Hemorrhagic Stroke | >150 min/wk vs. ≤150 min/wk | 0.93 (0.70–1.23) |  |  |  |
|  |  |  |  |  |  |  |  | strenuous LTPA, total Stroke | >75 min/wk vs. ≤75 min/wk | 0.91 (0.77–1.07) |  |  |  |
|  |  |  |  |  |  |  |  | strenuous LTPA, Ischemic Stroke | >75 min/wk vs. ≤75 min/wk | 0.89 (0.73–1.08) |  |  |  |
|  |  |  |  |  |  |  |  | strenuous LTPA, Hemorrhagic Stroke | >75 min/wk vs. ≤75 min/wk | 0.96 (0.69–1.33) |  |  |  |
| Zhao, 2020(154) | China | Electronic health check-up database Xinzheng City | Elderly population | 32,942/ CVD: 6,857, stroke:2240, CHD: 4937 | B, 6.84 | ≥68 | Age, sex, current smoking, alcohol, BMI, medical history of T2DM, SBP, DBP, RHR, FPG, TC, TG and HDL-C | LTPA | Very highly active vs. inactive | 1.08 (0.79-1.48) | Questionnaire, NM about validation | data of the annual standard health check-up record  with digital linkage to the hospital dataset for admissions. | Baseline |
| Abbreviations: AHEI, Alternate Healthy Eating Index; AMI, acute myocardial infarction; Atrial fibrillation, AF; B, both; BP, blood pressure; BMI, body mass index; CAD, coronary artery disease; CHD, coronary heart disease; CHF, congestive heart failure; CI, confidence interval; COPD, chronic obstructive pulmonary disease ; CRP, C-reactive protein; CVE, cardiovascular events; DBP, diastolic blood pressure; DM, diabetes mellitus; EE, energy expenditure; EI, energy intake; EPIC, European Prospective Investigation into Cancer and Nutrition; F, female; FBS, fasting blood sugar; FH, family history; GFR, glomerular filtration rate; HDL-C, high density lipoproteins cholesterol; HF, heart failure; HTN, hypertension; IHD, ischemic heart disease; LDL-C, low density lipoproteins cholesterol; LTPA, leisure time physical activity; LVH, left ventricular hypertrophy; M, Male; NHISNCHS, National Health Interview Survey of the National Center for Health Statistics; NHIS, National Health Insurance Service; NM, not mentioned; OPA, occupational physical activity; PA, physical activity; PAD, peripheral artery disease; PUFA, polyunsaturated fatty acid; PY, person-years; RR, risk ratio; SBP, systolic blood pressure; T-C, total cholesterol; TFA, trans fatty acids; TG, triglyceride; TIA, transient ischemic attack ; WC, waist circumference; WHI-OS, Women’s Health Initiative (WHI) Observational Study; wk, week | | | | | | | | | | | | | |

**Supplementary Table 8. Characteristic of studies investigating the relationship between LTPA and AF**

| **Author, year** | **Country** | **Study name** | **Population characteristics** | **Total participants, Case (num)** | **Sex, Duration (Year)** | **Age (range or mean)** | **Adjustment factors** | **Type of PA** | **PA levels** | **RR, 95% CI** | **Exposure assessment** | **Outcome assessment** | **PA timed assessed (Baseline, repeated)** |
| --- | --- | --- | --- | --- | --- | --- | --- | --- | --- | --- | --- | --- | --- |
| Aizer, 2009(190) | USA | Physicians’ Health Study | Male physicians | 16,921, AF: 1,661 | M, 12 | 40 − 84 | age, treatment assignment, BMI, history of DM, HTN, hyperlipidemia, parental premature MI, alcohol, smoking, fish consumption, multivitamin, vitamin C and E intake, LVH, CHF, and evidence of CVD | regular program of exercise vigorous enough to  sweat, at 3 Years | Non-Exercisers | 1.00 | Validated questionnaire | Self-reported by physician | Repeated |
|  |  |  |  |  |  |  |  |  | <1 day/wk | 0.90 (0.68, 1.20) |  |  |  |
|  |  |  |  |  |  |  |  |  | 1−2 days/wk | 1.09 (0.95, 1.26) |  |  |  |
|  |  |  |  |  |  |  |  |  | 3−4 days/wk | 1.04 (0.91, 1.19) |  |  |  |
|  |  |  |  |  |  |  |  |  | 5−7 days/wk | 1.20 (1.02−1.41) |  |  |  |
|  |  |  |  |  |  |  |  | regular program of exercise vigorous enough to  sweat, updated | Non-Exercisers | 1.00 |  |  |  |
|  |  |  |  |  |  |  |  |  | <1 day/wk | 1.14 (0.86−1.51) |  |  |  |
|  |  |  |  |  |  |  |  |  | 1−2 days/wk | 1.06 (0.91−1.23) |  |  |  |
|  |  |  |  |  |  |  |  |  | 3−4 days/wk | 1.01 (0.89−1.16) |  |  |  |
|  |  |  |  |  |  |  |  |  | 5−7 days/wk | 1.16 (0.99−1.36) |  |  |  |
| Albrecht 2018(191) | Netherlands | Rotterdam Study | subjects aged 55 years or older | 7018, AF: 800 | B, 16.8 | >55 (69.4) | Age, sex, other PA types, smoking, previous CVD, alcohol, diet, education, BMI, T-C and HDL-C, DM, lipid reducing agents, SBP, DBP, anti-thrombotic agents, ACE-inhibitor use | Sport | 0.0 MET-h/wk | 1.00 | adapted version of  the Zutphen Physical Activity Questionnaire (validated) | automated  follow-up system involving digital linkage of the  study database to medical records maintained by  general practitioners | Baseline |
|  |  |  |  |  |  |  |  |  | 5.5 MET-h/wk | 0.99 (0.82–1.19) |  |  |  |
|  |  |  |  |  |  |  |  |  | 19.8 MET-h/wk | 0.99 (0.82–1.20) |  |  |  |
| Azarball 2014(107) | USA | WHI-OS | postmenopausal  women | 81317, AF: 9792 | F, 11.5 | 63.4 | Age, race, BMI, smoking, alcohol, education, income, DM, HTN, hyperlipidemia, CAD, PAD | LTPA | No activity | 1.00 | Validated questionnaire | Self-report + Medicare and Medicaid Services data which were available only for some participants | Repeated |
|  |  |  |  |  |  |  |  |  | >0 to 3 MET-h/wk | 0.98 (0.91, 1.06) |  |  |  |
|  |  |  |  |  |  |  |  |  | >3 to 9 MET-h/wk | 0.94 (0.88, 1.01) |  |  |  |
|  |  |  |  |  |  |  |  |  | >9 MET-h/wk | 0.90 (0.85, 0.96) |  |  |  |
| Drca, 2014(192) | Sweden | Cohort of Swedish Men | General population | 44410 /AF: 4568 | M, 12 | 45-79  (60) | Age; education; smoking; BMI; DM; history of HTN, CHD, or, HF; FH of MI, aspirin use, alcohol, walking/bicycling | LTPA | < 1 h/wk | 1.00 | Validated questionnaire | National  Patient Register | Baseline |
|  |  |  |  |  |  |  |  |  | 1 h/wk | 0.98 (0.87, 1.1) |  |  |  |
|  |  |  |  |  |  |  |  |  | 2-3 h/wk | 1.01 (0.91, 1.12) |  |  |  |
|  |  |  |  |  |  |  |  |  | 4-5 h/wk | 0.96 (0.84, 1.09) |  |  |  |
|  |  |  |  |  |  |  |  |  | >5 h/wk | 1.05 (0.92, 1.18) |  |  |  |
| Everett, 2011(106) | US | Women’s Health Study | Female health professionals | 34759/ AF: 968 | F, 14.4 | >45, 53 | BMI, Age, randomized treatment, cholesterol, current smoking, past smoking, alcohol, DM, race | LTPA | < 7.5 MET-h/wk vs. ≥7.5 MET-h/wk | 0.97 (0.84–1.11) | validated self-reported questionnaire | Self-report | Repeated |
| Fletcher 2022(103) | US | Atherosclerosis Risk in Communities (ARIC) study | Elderlies (persons > 65 years old) | 5116/ AF: 703 | B, 6.3 | 75 | Age, sex, race, education, alcohol, cigarette smoking, DM, HTN, prevalent CHD, prevalent HF, BMI, Short Physical Performance Battery score | LTPA | High MVPA vs. low MVPA | 0.97 (0.78, 1.21) | Hospital discharge diagnoses and death certificates | valid Modified Baecke Physical Activity Questionnaire | Baseline |
| Garnvik, 2018(146) | Norway | HUNT 3 | Obese individuals | 43602/ AF: 1459 | B, 8.1 | > 18 | Age, sex, current smoking, alcohol use, self-reported CVD and occupational status, BMI. | LTPA | High vs. inactive | 0.91 (0.77–1.09) | answering three questions concerning frequency, intensity and duration (validated) | Hospital records | Baseline |
| Jin 2019(193) | Korea | Korean NHIS | General population | 501690, AF: 3443 | B, 4 | >18, 47.6 | age, sex, BMI, HF, HTN, DM, previous MI, prior stroke or  transient ischemic attack, CKD, smoking, alcohol | LTPA | None | 1.00 | IPAQ (non-validated) | presence of one hospitalization or two outpatient visits (validated) | Baseline |
|  |  |  |  |  |  |  |  |  | 1 to <500 MET min/week | 0.94 (0.86–1.03) |  |  |  |
|  |  |  |  |  |  |  |  |  | 500 to <1000 MET min/week | 0.88 (0.80–0.97) |  |  |  |
|  |  |  |  |  |  |  |  |  | ≥ 1000 MET min/wk | 0.93 (0.85–1.03) |  |  |  |
| Johansen 2022(147) 1 | Norway | Birkebeiner Ageing Study and the Tromsø Study | Malenon- athletes aged ≥65 | 1807, AF: 325 | M, 10 | 69.8 | Age, body height, education, smoking, alcohol, CHD (AF only), DM (AF only), BMI and antihypertensive  medication. | LTPA | Moderate to vigorous vs. non-active | 1.04 (0.70, 1.53) | Validated self-reported assessed by the Saltin-Grimby Physical Activity Level Scale | Self-reported and confirmed AF diagnoses by electrocardiography were retrieved from medical hospital records | Baseline |
| Knuiman, 2014(194) | Australia | The Busselton Health Study | General population | 4,267/ AF: 343 | B, 15 | 25–84  (52) | sex, age, height,  HTN treatment and BMI | LTPA (Exercis) | Exercise—some vigorous exercise each week vs. none | 0.80 (0.62, 1.03) | One question (doing any vigorous exercise in a usual week, not validated) | hospital admission  with a primary or other diagnosis of AF | Baseline |
| Mokhayeri, 2018(195) | USA | Multi-Ethnic Study of Atherosclerosis (MESA) | General population | 6,487, AF: 242 | B, 11 | 45-84  (62.4) | Race, sex, age, income, smoking, BMI, alcohol, HTN, total and HDL cholesterol, DM | walking for exercise, sports/dancing, Men | None | 1.00 | MESA Typical Week Physical Activity Survey (TWPAS), a self-reported standardized questionnaire, validated | Hospitalizations. cases associated with open surgery was excluded, all AF events was adjudicated | Baseline |
|  |  |  |  |  |  |  |  |  | 1- 14 MET-hr/wk | 1.28 (0.59, 2.77) |  |  |  |
|  |  |  |  |  |  |  |  |  | 15- 29 MET-hr/wk | 1.02 (0.42, 2.44) |  |  |  |
|  |  |  |  |  |  |  |  |  | ≥ 30 MET-hr/wk | 1.27 (0.57, 2.83) |  |  |  |
|  |  |  |  |  |  |  |  | walking for exercise, sports/dancing, Women | None | 1.00 |  |  |  |
|  |  |  |  |  |  |  |  |  | 1- 14 MET-hr/wk | 0.69 (0.29, 1.65) |  |  |  |
|  |  |  |  |  |  |  |  |  | 15- 29 MET-hr/wk | 0.54 (0.18, 1.63) |  |  |  |
|  |  |  |  |  |  |  |  |  | ≥ 30 MET-hr/wk | 0.53 (0.17, 1.66) |  |  |  |
| Skielboe, 2016(112) | Denmark | Copenhagen City Heart Study | General population | 17196/ AF: 338 | B, 20.3 | 20- 93 | Age, height, BMI, sex, smoking, drinking habits, education, BP, resting heart rate, spirometry, cardiac medication, DM, IHD and enrolment number | LTPA | Low volume (sedentary) | 0.92 (0.77−1.09) | CHS Physical Activity Questionnaire | National patient registry | Baseline |
|  |  |  |  |  |  |  |  |  | Moderate volume, light PA for 2–4 h/wk | 1.00 |  |  |  |
|  |  |  |  |  |  |  |  |  | high volume, light PA for >4 h/wk or more vigorous activity for 2–  4 h/wk | 1.05 (0.92−1.20) |  |  |  |
|  |  |  |  |  |  |  |  |  | very high volume, highly vigorous PA for >4 h/wk or regular heavy exercise or competitive sports several times/ wk | 1.16 (0.80−1.67) |  |  |  |

1 Only data of non-athlete cohort was used.

Abbreviations: Atrial fibrillation, AF; B, both; BP, blood pressure; BMI, body mass index; CAD, coronary artery disease; CE, coronary events; CHD, coronary heart disease; CHF, congestive heart failure; CI, confidence interval; CRP, C-reactive protein; CVE, cardiovascular events; DBP, diastolic blood pressure; DM, diabetes mellitus; EE, energy expenditure; EI, energy intake; F, female; FBS, fasting blood sugar; FH, family history; HDL-C, high density lipoproteins cholesterol; HF, heart failure; HTN, hypertension; IHD, ischemic heart disease; LDL-C, low density lipoproteins cholesterol; LTPA, leisure time physical activity; LVH, left ventricular hypertrophy; M, Male; NHIS, National Health Insurance Service; NM, not mentioned; PA, physical activity; PAD, peripheral artery disease; PUFA, polyunsaturated fatty acid; PY, person-years; RR, risk ratio; SBP, systolic blood pressure; T-C, total cholesterol; TFA, trans fatty acids; TG, triglyceride; TIA, transient ischemic attack ; WC, waist circumference; WHI-OS, Women’s Health Initiative (WHI) Observational Study; wk, week

**Supplementary Table 9. Characteristic of studies investigating the relationship between OPA and CVD**

| **Author, year** | **Country** | **Study name** | **Population characteristics** | **Total participants, Type of CVD (num)** | **Sex, Duration (Year)** | **Age (range or mean)** | **Adjustment factors** | **Type of PA** | **PA levels** | **HR, 95% CI** | **Exposure assessment** | **Outcome assessment** | **PA timed assessed (Baseline, repeated)** |
| --- | --- | --- | --- | --- | --- | --- | --- | --- | --- | --- | --- | --- | --- |
| Bakker 2021(158) | Netherland | Lifelines Cohort Study | General population | 142493, CVD:1019, | B, 6.8 | > 18 | Age, sex, income, education, alcohol, smoking, nutrient intake, fat, carbohydrate, kidney function, arrhythmia, hypothyroid, lung disease, osteoarthritis, RA | OPA | High vs. low | 0.79 (0.55, 1.13) | Validated questionnaire | Hospital records | Baseline |
| Bennett, 2017(196) | China | China Kadoorie Biobank (CKB) | General population | 487334/ Major vascular events: 36184 | B, 7.5 | 51 | age at risk, sex, region, income, education, BMI, alcohol, smoking, SBP, fresh fruit intake, sedentary leisure time, and self-rated health. OPA additionally adjusted for non-OPA. | OPA | 0 MET-h/day | 1.00 | Self-reported questionnaire but has not been compared directly with a reference method (accelerometer) | Local chronic disease registries | Baseline |
|  |  |  |  |  |  |  |  |  | 0.1-5.9 MET-h/day | 0.87 (0.84, 0.90) |  |  |  |
|  |  |  |  |  |  |  |  |  | 6-13.8 MET-h/day | 0.80 (0.78, 0.82) |  |  |  |
|  |  |  |  |  |  |  |  |  | 13.9-25.7 MET-h/day | 0.77 (0.75, 0.80) |  |  |  |
|  |  |  |  |  |  |  |  |  | ≥25.8 MET-h/day | 0.77 (0.74, 0.79) |  |  |  |
| Ferrario, 2018(134) | Italy | 3 population-based cohorts (the MONICA Brianza surveys II & III; the PAMELA; and one factory-based (SEMM) | Men working as salaried employees | 3574/ CVD: 174 | M, 14 | 25–64 | Age, cohort, educational level, BMI, total and HDL cholesterol, SBP, current smoking, DM and alcohol intake | OPA | Low | 1.50 (1.01, 2.23) | Baecke Questionnaire  (validated) | Hospital records | Baseline |
|  |  |  |  |  |  |  |  |  | Intermediate | 1.00 |  |  |  |
|  |  |  |  |  |  |  |  |  | High | 1.20 (0.78, 1.84) |  |  |  |
| Hu, 2014(149) | Taiwan | Chin-Shan Community Cardiovascular Cohort study | General population | 1706/ CVD: 215 | B, 16.8 |  | Age, sex, BMI, marital status, job, education, other type PA, smoking, alcohol, HTN &DM at baseline, SBP, DBP, TG, uric acid, FBS, lipid profiles | OPA | Highest tertile vs. lowest tertile | 1.75 (1.10, 2.80) | Baecke questionnaire | Imaging data and medical records | Repeated |
| Holtermann, 2021(167) | Denmark | Copenhagen General Population Study | General population | 75195/ Major adverse CVE: 5813 | B, 10 | 20–100 | Age, sex, education, BMI, smoking, DM, SBP, BP medication, alcohol, dietary preferences, LDL-C, HDL-C, TG, COPD; an extended multivariable sensitivity, resting heart rate, vital exhaustion score, occupation, cohabitation, marital status, income | OPA | Very high vs. low | 1.35 (1.13, 1.61) | Question  (non-validated) | Hospital record | Baseline |
| Khaw 2006(71) | UK | EPIC-Norfolk | General population | 22191/ CVD (fatal+ non-fatal): 3079 | B, 8 | 45–79 | Age, Sex, BMI, SBP, cholesterol, smoking, alcohol, known diabetes, and social class | OPA | Heavy manual vs. sedentary | 0.91 (0.62, 1.33) | Two questions  Validated | hospital admission  and/or died with CVD as underlying cause of death | Baseline |
| Kuster, 2021(144) | Sweden | Attitude, Behavior, and Change Study | General population | 807/ CVD: 59 | B, 14.6 | 42.8 (18-75) | Age, sex, education and previous disease | OPA | Active vs. sedentary | 1.65 (0.73–3.74) | Self-report questionnaire  (Validated) | National patient register | Baseline |
| B, both; BP, blood pressure; BMI, body mass index; CE, coronary events; CI, confidence interals; COPD, chronic obstructive pulmonary disease ; DBP, diastolic blood pressure; DM, diabetes mellitus; EE, energy expenditure; EI, energy intake; EPIC, European Prospective Investigation into Cancer and Nutrition; F, female; FBS, fasting blood sugar; FH, family history; HDL-C, high density lipoproteins cholesterol; HF, heart failure; HTN, hypertension; LDL-C, low density lipoproteins cholesterol; M, Male; MONICA, Multinational Monitoring of Trends and Determinants in Cardiovascular Disease; NM, not mentioned; OPA, occupational physical activity; PA, physical activity; PAD, peripheral artery disease; PAMELA, Pressioni Arteriose Monitorate Loro Associazioni; PY, person-years; RR, risk ratio; SEMM, Study of Employed in the Municipality of Milan, SBP, systolic blood pressure; T-C, total cholesterol; TFA, trans fatty acids; TG, triglyceride; WC, waist circumference; (WHI) Observational Study; wk, week | | | | | | | | | | | | | |

**Supplementary Table 10. Characteristic of studies investigating the relationship between OPA and CHD**

| **Author, year** | **Country** | **Study name** | **Population characteristics** | **Total participants, Case (num)** | **Sex, Duration (Year)** | **Age (range or mean)** | **Adjustment factors** | **Type of PA** | **PA levels** | **RR, 95%CI** | **Exposure assessment** | **Outcome assessment** | **PA timed assessed (Baseline, repeated)** |
| --- | --- | --- | --- | --- | --- | --- | --- | --- | --- | --- | --- | --- | --- |
| Allesøe, 2015(115) | Denmark | Danish Nurse Cohort Study | Nurses | 12093/ IHD: 580 | F, 15 | 45- 64 | Age, FH of IHD, HTN, DM, BMI, smoking, alcohol, LTPA, work pressure, job influence, shift work and work h/wk | OPA | High vs. sedentary | 1.19 (0.92, 1.54) | Saltin and Grimby questionnaire | National patient  register | Baseline |
| Alleose 2023(197) | Denmark | Danish Monica 1 | General population | 3105, CHD: 510 | B, 25.5 | 30–61 | Age, LTPA, smoking, alcohol, BMI, self-reported fitness, DM, serum cholesterol, HDL, TG, familial predisposition for heart disease, working hours, civil status, SES. | OPA | Strenuous, heavy lifting vs.  sedentary | 1.14 (0.75, 1.74) | single question, based on the Saltin and Grimby question | Linkage to the Danish National Patient Registry | Baseline |
| Bennett, 2017(196) | China | China Kadoorie Biobank (CKB) | General population | 487334/ major CE: | B, 7.5 | 51 | age at risk, sex, region, income, education, BMI, alcohol, smoking, SBP, fresh fruit intake, sedentary leisure time, and self-rated health. Non-OPA additionally adjusted for OPA | OPA | 0 MET-h/day | 1.00 | Self-reported questionnaire was not compared directly with a reference method (accelerometer) | Local chronic disease registries | Baseline |
|  |  |  |  |  |  |  |  |  | 0.1-5.9 MET-h/day | 0.77 (0.70, 0.84) |  |  |  |
|  |  |  |  |  |  |  |  |  | 6-13.8 MET-h/day | 0.70 (0.65, 0.76) |  |  |  |
|  |  |  |  |  |  |  |  |  | 13.9-25.7 MET-h/day | 0.69 (0.63, 0.76) |  |  |  |
|  |  |  |  |  |  |  |  |  | ≥25.8 MET-h/day | 0.68 (0.62, 0.76) |  |  |  |
| Clays, 2013(128) | Belgium | BELSTRESS | General population | 14337/ CHD: 87 | M, 3.15 | 35–59 | Age, LTPA, educational, BMI, occupational class, job strain, smoking, alcohol intake, DM, SBP, T-C, HDL_C | OPA | High vs. low | 1.28 (0.68–2.44) | Job Content Questionnaire, Validated | Physician diagnosis | Baseline |
| Ferrario, 2018(134) | Italy | 3 population cohorts (the MONICA Brianza surveys II & III; the PAMELA; & one factory-based (SEMM) | Men working as salaried employees | 3574/ CHD: 174 | M, 14 | 25–64 | Age, cohort, educational level, BMI, total and HDL cholesterol, SBP, current smoking, DM and alcohol intake | Sport, OPA | Low | 1.61 (1.02, 2.52) | Baecke Questionnaire  (validated) | Hospital records | Baseline |
|  |  |  |  |  |  |  |  |  | Intermediate | 1.00 |  |  |  |
|  |  |  |  |  |  |  |  |  | High | 1.26 (0.76, 2.08) |  |  |  |
| Holtermann, 2012(198) | Denmark | Copenhagen City Heart Study | General population | 4538/ MI: 276 | B, 17.8 | 25- 66 | Age, smoking, alcohol, BMI, LTPA, SBP, DM, cholesterol, blood pressure medication and household income | OPA, men | Low | 1.00 | single question with four categories  (unclear validation) | Hospital record | Repeated |
|  |  |  |  |  |  |  |  |  | Moderate | 1.30 (1.03, 1.64) |  |  |  |
|  |  |  |  |  |  |  |  |  | High | 1.20 (0.93, 1.55) |  |  |  |
|  |  |  |  |  |  |  |  | OPA, women | Low | 1.00 |  |  |  |
|  |  |  |  |  |  |  |  |  | Moderate | 0.76 (0.56, 1.02) |  |  |  |
|  |  |  |  |  |  |  |  |  | High | 0.98 (0.67, 1.44) |  |  |  |
| Hu, 2007(119) | Finland | 7 independent population surveys in 6 areas of Finland | General population | 21346/ CHD: 392 | B, 10 | 25- 64 | Study year, age, education, BMI, alcohol, FH of CHD, and FRS (total cholesterol, SBP, treatment for HTN, and cigarette), FH of CHD, OPA, commuting PA, LTPA | OPA, Male | Low | 1.00 | self-administered questionnaire, not validated | Hospital record | Baseline |
|  |  |  |  |  |  |  |  |  | Moderate | 0.66 (0.55–0.79) |  |  |  |
|  |  |  |  |  |  |  |  |  | High | 0.74 (0.65–0.85) |  |  |  |
|  |  |  |  |  |  |  |  | OPA, Female | Low | 1.00 |  |  |  |
|  |  |  |  |  |  |  |  |  | Moderate | 0.53 (0.40–0.70) |  |  |  |
|  |  |  |  |  |  |  |  |  | High | 0.58 (0.44–0.76) |  |  |  |
| Johnsen, 2016(199) | Sweden | Swedish Work, Lipids and Fibrinogen (WOLF) study | General population | 9961/ MI: 249 | B, 13.1 | 19- 70 | Age, sex, lifestyle factors (smoking, LTPA, alcohol, fruit and vegetable consumption) | OPA | seated for > 50% of working day, no lifting or carrying | 1.00 | Three questions in the questionnaire  (unclear validation) | National patient register | Baseline |
|  |  |  |  |  |  |  |  |  | standing or walking for > 50% of working day, no lifting or carrying | 1.15 (0.84, 1.58) |  |  |  |
|  |  |  |  |  |  |  |  |  | lifting or carrying regardless of whether seated or standing/walking | 0.90 (0.64, 1.28) |  |  |  |
| Krause, 2015 (200) | Finland | Kuopio Ischemic Heart Disease Risk Factor Study (KIHD) | Men aged 42, 48, 54,  or 60 years | 1891/ Acute MI: 495 | M,  19.56 | 19.56 | Age, BMI, alcohol, smoking, SES, Conditioning LTPA, LDL and HDL cholesterol, FBS, SBP, Lipid-lowering drug, Anti-HTN medication | OPA | Per 500 kcal/day | 1.00 (0.95, 1.06) | occupational interview, validated | medical examinations +  interview survey + record linkage with the national MI register established under the WHO MONICA project | Baseline |
| Merry, 2011(141) | Netherlands | CAREMA study | General population | 63444/ 472, Acute MI | B, 11.1 | 20- 59 | Age, sex, baseline cohort (PPHVZ or MORGEN), smoking, alcohol, total and HDL cholesterol, DM, SBP, and BMI | OPA, male | Moderate to heavy PA vs. none to light PA | 1.37 (1.05, 1.80) | Self-questionnaire  (unclear validation) | Cardiologic information system | Baseline |
|  |  |  |  |  |  |  |  | OPA, female | Moderate to heavy PA vs. none to light PA | 0.57 (0.25, 1.32) |  |  |  |
| Petersen, 2012(140) | Denmark | Danish National Health Interview Surveys | General population | 10689/MI:1384, IHD: 2824 | B, 13 | 16- 85 | Age, education, smoking, alcohol consumption, BMI, HTN, and self-perceived stress | OPA, male | High vs. low | 0.50 (0.37, 0.68) | Saltin and Grimby questionnaire | National patient register | Baseline |
|  |  |  |  |  |  |  |  | OPA, female | High vs. low | 1.55 (0.98, 2.44) |  |  |  |
| Strippoli, 2022(152) | Italy | Italian National Health Interview Surveys | General population | 40,220/ CHD: 1442 | B, 14 | 40–55 | Age, family type and macroeconomic region, general health status, smoking, BMI, education, other domains of PA | OPA | Heavy vs. light | 0.65 (0.34, 1.23) | Non-valid questionnaire | Linkage with  Hospital discharge records (Ministry of  Health) | Baseline |
| Abbreviations: AMI, acute myocardial infarction; Atrial fibrillation, AF; B, both; BP, blood pressure; BMI, body mass index; CE, coronary events; CHD, coronary heart disease; DBP, diastolic blood pressure; DM, diabetes mellitus; F, female; FBS, fasting blood sugar; HDL-C, high density lipoproteins cholesterol; HTN, hypertension; IHD, ischemic heart disease; LDL-C, low density lipoproteins cholesterol; LTPA, leisure time physical activity; M, Male; MONICA, Multinational Monitoring of Trends and Determinants in Cardiovascular Disease; NM, not mentioned; OPA, occupational physical activity; PA, physical activity; PY, person-years; SEMM, Study of Employed in the Municipality of Milan, SBP, systolic blood pressure; SES, socioeconomic status; T-C, total cholesterol; wk, week | | | | | | | | | | | | | |

**Supplementary Table 11. Characteristic of studies investigating the relationship between OPA and stroke**

| **Author, year** | **Country** | **Study name** | **Population characteristics** | **Total participants, Type of CVD (num)** | **Sex, Duration (Year)** | **Age (range or mean)** | **Adjustment factors** | **Type of PA** | **PA levels** | **RR, 95% CI** | **Exposure assessment** | **Outcome assessment** | **PA timed assessed (Baseline, repeated)** |
| --- | --- | --- | --- | --- | --- | --- | --- | --- | --- | --- | --- | --- | --- |
| Bennett, 2017(196) | China | China Kadoorie Biobank (CKB) | General population | 487334/ ischemic stroke: 25647, intracerebral  Hemorrhage: 5252 | B, 7.5 | 51 | Age at risk, sex, region, income, education, BMI, alcohol, smoking, SBP, fresh fruit intake, sedentary leisure time, and self-rated health. OPA additionally adjusted for non-OPA. Non-OPA additionally adjusted for OPA | OPA, ischemic stroke | 0 MET-h/day | 1.00 | Self-reported questionnaire but has not been compared directly with a reference method (accelerometer) | Local chronic disease registries | Baseline |
|  |  |  |  |  |  |  |  |  | 0.1-5.9 MET-h/day | 0.89 (0.86, 0.93) |  |  |  |
|  |  |  |  |  |  |  |  |  | 6-13.8 MET-h/day | 0.83 (0.81, 0.86) |  |  |  |
|  |  |  |  |  |  |  |  |  | 13.9-25.7 MET-h/day | 0.82 (0.79, 0.85) |  |  |  |
|  |  |  |  |  |  |  |  |  | ≥25.8 MET-h/day | 0.79 (0.75, 0.82) |  |  |  |
|  |  |  |  |  |  |  |  | OPA, intracerebral  Hemorrhage | 0 MET-h/day | 1.00 |  |  |  |
|  |  |  |  |  |  |  |  |  | 0.1-5.9 MET-h/day | 0.87 (0.81, 0.94) |  |  |  |
|  |  |  |  |  |  |  |  |  | 6-13.8 MET-h/day | 0.75 (0.71, 0.80) |  |  |  |
|  |  |  |  |  |  |  |  |  | 13.9-25.7 MET-h/day | 0.7 (0.65, 0.75) |  |  |  |
|  |  |  |  |  |  |  |  |  | ≥25.8 MET-h/day | 0.75 (0.69, 0.82) |  |  |  |
| Blomstrand, 2022(121) | Sweden | Population Study of Women in Gothenburg | General population | 1462/ Stroke: 172 | F, 44 | 60-82 | Age, sex | OPA | Professional work vs. no work | 0.92 (0.51, 1.00) | NM about validation of Questionnaire | National patient register | Baseline |
| Hall, 2019(185) | USA | Sister study | working women | 31270 / transient ischemic attack + Stroke: 715 | F, 5.7 | 35–74 | Age, LTPA, alcohol, smoking, BMI, discrimination at work, night work, and rate pressure product. | OPA, Total stroke | High intensity work vs. mostly sitting | 1.22 (0.80, 1.84) | computer-assisted telephone interviews (non-validated) | Self-reported doctor’s diagnoses | Baseline |
|  |  |  |  |  |  |  |  | OPA, TIA | High intensity work vs. mostly sitting | 1.57 (1.04, 2.38) |  |  |  |
| Hu, 2005(120) | Finland | 7 independent population surveys in 6 areas of Finland | General population | 47721/ Stroke: 2863 | B, 19 | 25- 64 | Age, area, and study year, MI, SBP, cholesterol, education, smoking, alcohol, and DM, other 2 types of PA | OPA, total | Active vs. light | 0.89 (0.81–0.98) | Self-reported questionnaire, not validated | Hospital record | Baseline |
|  |  |  |  |  |  |  |  | OPA, Males | Active vs. light | 0.90 (0.80–1.03) |  |  |  |
|  |  |  |  |  |  |  |  | OPA, females | Active vs. light | 0.89 (0.78–1.03) |  |  |  |
| Huerta, 2013(186) | Spain | EPIC-Spain | General population | 32992/ transient ischemic attack: 112, Stroke:271 | B, 12.3 | 29-69 | Age, center, educational level, self-reported HTN or hyperlipidemia, DM, smoking, age at start smoking, alcohol, EI, BMI, WC, intake of protein, lipids, vegetables, red meat, and fish, PA | OPA, total stroke | Manual/heavy manual occupation vs.  Sedentary occupation | 0.99 (0.56, 1.72) | EPIC-PAQ included 9 specific questions | Self-report and computerized primary care registers | Baseline |
|  |  |  |  |  |  |  |  | OPA, TIA | Manual/heavy manual occupation vs.  Sedentary occupation | 0.94 (0.66–1.33) |  |  |  |
| Johansson 2022(126) | Sweden | Malmö Diet and Cancer Study cohort | General population | 23797, atherothrombotic ischemic stroke: 1937 | B, 21.5 | 58 | Age, sex, smoking, education, stroke heredity, dyslipidemia, HTN, BMI and diet quality index score | OPA | Q4 vs. Q1 | 1.05 (0.91-1.22) | Linkage to National Patient register and the Cause of Death Register Participants | Self-reported questionaire- NR about validation | Baseline |
| Abbreviations: B, both; BP, blood pressure; BMI, body mass index; CE, coronary events; CHD, coronary heart disease; DBP, diastolic blood pressure; DM, diabetes mellitus; F, female; HTN, hypertension; LTPA, leisure time physical activity; M, Male; NM, not mentioned; OPA, occupational physical activity; PA, physical activity; PY, person-years; SBP, systolic blood pressure; wk, week | | | | | | | | | | | | | |

**Supplementary Table 12. Characteristic of studies investigating the relationship between OPA and AF**

| **Author, year** | **Country** | **Study name** | **Population characteristics** | **Total participants, Type of CVD (num)** | **Sex, Duration (Year)** | **Age (range or mean)** | **Adjustment factors** | **Type of PA** | **PA level** | **RR, 95% CI** | **Exposure assessment** | **Outcome assessment** | **PA timed assessed (Baseline, repeated)** |
| --- | --- | --- | --- | --- | --- | --- | --- | --- | --- | --- | --- | --- | --- |
| Drca 2021(192) | Sweden | Cohort of Swedish Men+ Swedish Mammography Cohort | General population | 36512/ AF: 4697 | B, 17 | 49–83 | Age, education, smoking, BMI, walking or bicycling, history of cardiac disease, HTN, and DM, FH of MI before age 60, alcohol | OPA, men | Low | 1.00 | Validated questionnaire | National  Patient Register | Baseline |
|  |  |  |  |  |  |  |  |  | Medium | 1.06 (1.00–1.12) |  |  |  |
|  |  |  |  |  |  |  |  |  | High | 1.19 (1.10–1.29) |  |  |  |
|  |  |  |  |  |  |  |  | OPA, women | Low | 1.00 |  |  |  |
|  |  |  |  |  |  |  |  |  | Medium | 0.98 (0.92–1.05) |  |  |  |
|  |  |  |  |  |  |  |  |  | High | 1.11 (0.99–1.24) |  |  |  |
| Skielboe, 2016(112) | Denmark | Copenhagen City Heart Study | General population | 17196/ AF: 338 | B, 20.3 | 20- 93 | Age, height, BMI, sex, smoking, drinking habits, education, BP, resting heart rate, spirometry, cardiac medication, DM, IHD and enrolment number | LTPA, OPA | Very high vs. moderate volume | 1.39 (1.03−1.88) | CHS Physical Activity Questionnaire | National patient registry | Baseline |

Abbreviations: Atrial fibrillation, AF; B, both; BP, blood pressure; BMI, body mass index; DM, diabetes mellitus; CI, confidence intervals; F, female; FH, family history; HTN, hypertension; IHD, ischemic heart disease; M, Male; OPA, occupational physical activity; PA, physical activity; RR, risk ratio

**Supplementary Table13. The risk of bias assessment by the Cochrane’s ROBINS-I tool (Cochrane Risk of bias in Non-randomized Studies of Interventions).**

| **Study** | **Bias due to confounding** | **Bias due to selection of participants** | **Bias due to exposure assessment** | **Bias due to misclassification during follow-up** | **Bias due to missing data** | **Bias due to measurement of outcome** | **Bias due to selective reporting of the results** | **Overall judgment** |
| --- | --- | --- | --- | --- | --- | --- | --- | --- |
| Acosta 2021 | Moderate | Low | Moderate | Moderate | Low | Moderate | Low | Moderate |
| Agnarsson, 1999 | Serious | Low | Serious | Moderate | Low | Moderate | Low | Serious |
| Ahmed, 2020 | Serious | Low | Moderate | Moderate | Low | Moderate | Low | Serious |
| Aizer, 2009 | Moderate | Moderate | Moderate | Low | Low | Moderate | Low | Moderate |
| Akesson, 2007 | Serious | Low | Moderate | Moderate | Low | Moderate | Low | Serious |
| Akesson, 2014 | Moderate | Low | Moderate | Moderate | Low | Moderate | Low | Moderate |
| Allesøe, 2015 | Serious | Moderate | Moderate | Moderate | Low | Moderate | Low | Serious |
| Allesøe, 2023 | Serious | Low | Moderate | Moderate | Low | Moderate | Low | Serious |
| Amidei 2022 | Serious | Moderate | Moderate | Low | Serios | Moderate | Low | Serious |
| Andersen, 2014 | Serious | Low | Moderate | Moderate | Moderate | Moderate | Low | Serious |
| Armstrong, 2015 | Serious | Low | Serious | Low | Low | Moderate | Low | Serious |
| Azarball, 2014 | Serious | Moderate | Moderate | Low | Low | Moderate | Low | Serious |
| Bakker, 2021 | Serious | Low | Moderate | Moderate | Low | Serious | Low | Serious |
| Barengo, 2016 | Serious | Moderate | Serious | Moderate | Serious | Moderate | Low | Serious |
| Bell, 2013 | Serious | Low | Moderate | Low | Low | Moderate | Low | Serious |
| Bennett, 2017 | Moderate | Low | Serious | Moderate | Low | Moderate | Low | Serious |
| Blomstrand, 2014 | Serious | Low | Serious | Moderate | Low | Moderate | Low | Serious |
| Blomstrand, 2022 | Serious | Low | Serious | Moderate | Serious | Moderate | Low | Serious |
| Calling, 2006 | Serious | Low | Moderate | Moderate | Low | Moderate | Low | Serious |
| Chomistek, 2012 | Moderate | Moderate | Moderate | Low | Low | Serious | Low | Serious |
| Chomistek, 2013 | Moderate | Moderate | Moderate | Low | Low | Serious | Low | Serious |
| Chomistek, 2016 | Serious | Low | Moderate | Low | Low | Moderate | Low | Serious |
| Chomistek, 2018 | Serious | Low | Moderate | Low | Low | Moderate | Low | Serious |
| Clara, 2021 | Serious | Low | Moderate | Low | Low | Moderate | Low | Serious |
| Clays, 2013 | Serious | Low | Moderate | Moderate | Low | Moderate | Low | Serious |
| Cuthbertson, 2019 | Serious | Low | Moderate | Low | Low | Moderate | Low | Serious |
| Djousse 2021 | Moderate | Moderate | Serious | Moderate | Low | Moderate | Low | Serious |
| Drca 2014 | Serious | Low | Moderate | Moderate | Low | Moderate | Low | Serious |
| Drca, 2021 | Serious | Low | Moderate | Low | Low | Moderate | Low | Serious |
| Ekblom-Bak, 2021 | Serious | Moderate | Moderate | Moderate | Low | Moderate | Low | Serious |
| Elliott, 2020 | Serious | Moderate | Moderate | Low | Low | Moderate | Low | Serious |
| Elwood, 2013 | Serious | Low | Serious | Low | Low | Moderate | Low | Serious |
| Everett, 2011 | Serious | Moderate | Moderate | Low | Low | Serious | Low | Serious |
| Fernandez 2022 | Moderate | Low | Moderate | Moderate | Low | Moderate | Low | Moderate |
| Ferrario, 2018 | Serious | Low | Moderate | Moderate | Low | Moderate | Low | Serious |
| Fltecher, 2022 | Serious | Moderate | Moderate | Moderate | Low | Moderate | Low | Serious |
| Florido, 2016 | Serious | Moderate | Moderate | Moderate | Low | Moderate | Low | Serious |
| Ford, 2014 | Serious | Low | Serious | Moderate | Low | Moderate | Low | Serious |
| Fu, 2018 | Serious | Moderate | Moderate | Moderate | Moderate | Moderate | Low | Serious |
| Garnvik, 2018 | Serious | Moderate | Moderate | Moderate | Low | Moderate | Low | Serious |
| Gillum, 1996 | Serious | Moderate | Serious | Moderate | Low | Moderate | Low | Serious |
| Greenlee, 2017 | Serious | Moderate | Moderate | Moderate | Low | Moderate | Low | Serious |
| Gunnell, 2014 | Serious | Low | Serious | Moderate | Serious | Moderate | Low | Serious |
| Haapanen, 1997 | Serious | Low | Serious | Low | Serious | Moderate | Low | Serious |
| Hall, 2019 | Serious | Moderate | Serious | Moderate | Low | Moderate | Low | Serious |
| Harmsen, 2006 | Serious | Moderate | Serious | Moderate | Low | Moderate | Low | Serious |
| Hidalgo-Santamaria, 2018 | Serious | Low | Moderate | Moderate | Low | Moderate | Low | Serious |
| Hoevenaar-Blom, 2010 | Serious | Low | Moderate | Low | Low | Moderate | Low | Serious |
| Holtermann, 2012 | Serious | Low | Serious | Low | Serious | Moderate | Low | Serious |
| Holtermann, 2021 | Serious | Low | Serious | Moderate | Low | Moderate | Low | Serious |
| Hu, 2005 | Serious | Low | Serious | Moderate | Low | Moderate | Low | Serious |
| Hu, 2007 | Serious | Low | Serious | Moderate | Low | Moderate | Low | Serious |
| Hu, 2014 | Serious | Low | Moderate | Low | Low | Moderate | Low | Serious |
| Huerta, 2013 | Moderate | Low | Moderate | Moderate | Low | Moderate | Low | Moderate |
| Hummel, 2022 | Serious | Low | Moderate | Moderate | Low | Moderate | Low | Serious |
| Jasiukaitiene, 2020 | Serious | Low | Serious | Moderate | Serious | Serious | Low | Serious |
| Jefferis, 2014 | Serious | Moderate | Moderate | Low | Low | Moderate | Low | Serious |
| Jensen, 2008 | Serious | Low | Serious | Moderate | Low | Moderate | Low | Serious |
| Jeong, 2017 | Serious | Low | Serious | Moderate | Serious | Moderate | Low | Serious |
| Jin, 2019 | Serious | Moderate | Serious | Moderate | Low | Moderate | Low | Serious |
| Johansen 2022 | Serious | Low | Serious | Moderate | Low | Moderate | Low | Serious |
| Johansson, 2021 | Moderate | Low | Moderate | Moderate | Low | Moderate | Low | Moderate |
| Johansson, 2022 | Serious | Low | Serious | Moderate | Low | Moderate | Low | Serious |
| Johnsen, 2016 | Moderate | Low | Serious | Moderate | Low | Moderate | Low | Serious |
| Kaprio, 2000 | Serious | Moderate | Serious | Moderate | Serious | Moderate | Low | Serious |
| Khaw 2006 | Serious | Low | Moderate | Moderate | Low | Moderate | Low | Serious |
| Kim, 2019 | Serious | Low | Moderate | Low | Moderate | Moderate | Low | Serious |
| Knuiman, 2014 | Serious | Low | Serious | Moderate | Low | Moderate | Low | Serious |
| Koolhaas, 2016 | Serious | Moderate | Moderate | Moderate | Low | Moderate | Low | Serious |
| Kruase 2015 | Serious | Low | Moderate | Moderate | Serious | Moderate | Low | Serious |
| Kuster, 2021 | Serious | Low | Moderate | Moderate | Low | Moderate | Low | Serious |
| Lacey, 2015 | Serious | Low | Serious | Moderate | Low | Moderate | Low | Serious |
| Lakka, 1994 | Serious | Low | Moderate | Moderate | Serious | Moderate | Low | Serious |
| Larsson, 2014 | Moderate | Low | Serious | Moderate | Low | Moderate | Low | Serious |
| Lear 2017 | Serious | Low | Moderate | Moderate | Low | Moderate | Low | Serious |
| Lee 1998 | Serious | Moderate | Moderate | Moderate | Low | Serious | Low | Serious |
| Meneton, 2016 | Serious | Moderate | Serious | Moderate | Serous | Moderate | Low | Serious |
| Merry, 2011 | Serious | Low | Serious | Moderate | Low | Moderate | Low | Serious |
| Mokhayeri, 2018 | Serious | Low | Moderate | Moderate | Low | Moderate | Low | Serious |
| Mu, 2021 | Moderate | Low | Serious | Moderate | Serious | Moderate | Low | Serious |
| Patel, 2013 | Serious | Moderate | Moderate | Moderate | Low | Moderate | Low | Serious |
| Paudel 2023 | Moderate | Low | Moderate | Moderate | Low | Moderate | Low | Moderate |
| Petersen, 2012 | Serious | Low | Moderate | Moderate | Serious | Moderate | Low | Serious |
| Raza, 2021 | Serious | Low | Serious | Moderate | Low | Moderate | Low | Serious |
| Renningera, 2018 | Serious | Low | Moderate | Moderate | Low | Moderate | Low | Serious |
| Rist, 2021 | Serious | Moderate | Serious | Low | Low | Moderate | Low | Serious |
| Rosolova, 1994 | Serious | Moderate | Serious | Moderate | Serious | Moderate | Low | Serious |
| Sesso, 1998 | Serious | Moderate | Moderate | Moderate | Low | Serious | Low | Serious |
| Sesso, 2000 | Serious | Moderate | Moderate | Moderate | Low | Serious | Low | Serious |
| Sieverdes 2017 | Serious | Low | Moderate | Moderate | Serious | Serious | Low | Serious |
| Skielboe, 2016 | Serious | Low | Moderate | Moderate | Low | Moderate | Low | Serious |
| Stamatakis 2009 | Serious | Low | Moderate | Moderate | Low | Moderate | Low | Serious |
| Strippoli, 2022 | Serious | Low | Serious | Moderate | Low | Moderate | Low | Serious |
| Sundquist, 2022 | Serious | Low | Moderate | Moderate | Moderate | Moderate | Low | Serious |
| Tanasescu, 2002 | Moderate | Moderate | Moderate | Moderate | Low | Moderate | Low | Moderate |
| Thomas, 2020 | Serious | Low | Moderate | Moderate | Low | Moderate | Low | Serious |
| van Sloten, 2018 | Serious | Moderate | Serious | Low | Low | Moderate | Low | Serious |
| Wagner, 2002 | Serious | Moderate | Moderate | Low | Low | Moderate | Low | Serious |
| Weinstein, 2008 | Serious | Moderate | Moderate | Moderate | Low | Moderate | Low | Serious |
| Willey NOMAS, 2017 | Serious | Moderate | Moderate | Moderate | Low | Moderate | Low | Serious |
| Willey CTS, 2017 | Serious | Moderate | Moderate | Moderate | Low | Moderate | Low | Serious |
| Yang, 2021 | Serious | Moderate | Serious | Moderate | Low | Moderate | Low | Serious |
| Zhao, 2020 | Serious | Moderate | Serious | Moderate | Low | Moderate | Low | Serious |

Supplementary Table 14. Subgroup analysis of studies assessing relationship between leisure-time physical activity and cardiovascular diseases incidence according to potential important factors, high vs. low analysis

| **Subgroup factor** | | | | **N of study** | **HR (95%CI)** | ***I*^2^ (%)** | **P heterogeneity** | **P between**  **group** |
| --- | --- | --- | --- | --- | --- | --- | --- | --- |
| All studies | - | | | 40 | 0.81(0.77, 0.86) | 91.9 | <0.001 | - |
| Sex^1^ | Male | | | 9 | 0.77 (0.71, 0.84) | 29.2 | 0.19 | 0.36 |
|  | Female | | | 8 | 0.84 (0.77, 0.92) | 77.3 | <0.001 |  |
|  | Both | | | 26 | 0.81 (0.75, 0.88) | 93.3 | <0.001 |  |
| Risk of bias | Moderate | | | 3 | 0.92 (0.76, 1.11) | 76.9 | <0.001 | 0.17 |
|  | Serious | | | 37 | 0.81 (0.77, 0.84) | 87.9 | <0.001 |  |
| Risk of bias^2^ | Moderate | |  | 6 | 0.89(0.78, 1.00) | 84.1 | <0.001 | 0.13 |
|  | Serious | |  | 34 | 0.80 (0.77, 0.84) | 76.6 | <0.001 |  |
| Follow-up duration | ≤13 year | | | 21 | 0.82 (0.76, 0.89) | 94.6 | <0.001 | 0.56 |
|  | >13 year | | | 19 | 0.80 (0.74, 0.86) | 73.1 | <0.001 |  |
| Number of cases | ≤500 | | | 14 | 0.79 (0.73, 0.86) | 40.3 | 0.06 | 0.58 |
|  | >500 | | | 26 | 0.82 (0.77, 0.87) | 94.3 | <0.001 |  |
| Geographical location | Europe | | | 24 | 0.84 (0.79, 0.89) | 87.9 | 0.001 | 0.18 |
|  | USA | | | 9 | 0.77 (0.70, 0.86) | 88.0 | <0.001 |  |
|  | East Asia | | | 4 | 0.77 (0.71, 0.84) | 36.1 | 0.20 |  |
|  | Australia | | | 2 | 0.81 (0.73, 0.89) | 0.0 | 0.47 |  |
|  | Multiple continents | | | 1 | 0.93 (0.80, 1.08) | - | - |  |
| Time of PA assessment | Baseline | | | 27 | 0.80 (0.74, 0.87) | 92.9 | <0.001 | 0.64 |
|  | Updated | | | 13 | 0.82 (0.77, 0.87) | 76.1 | <0.001 |  |
| PA assessment | Validated | | | 26 | 0.81 (0.75, 0.87) | 89.2 | <0.001 | 0.83 |
|  | Non-validated | | | 14 | 0.82 (0.76, 0.87) | 81.6 | <0.001 |  |
| Outcome assessment | Non-self-reported | | | 33 | 0.80 (0.76, 0.85) | 92.7 | <0.001 | 0.21 |
|  | Self-reported | | | 4 | 0.79 (0.70, 0.89) | 48.8 | 0.12 |  |
|  | Mixed | | | 1 | 0.93 (0.80, 1.08) | - | - |  |
|  | Not mentioned | | | 1 | 0.91 (0.73, 1.13) | - | - |  |
| Type of PA | Sport/exercise | | | 7 | 0.78 (0.65, 0.93) | 89.0 | <0.001 | <0.001^4^/  0.09^5^ |
|  | Walking | | | 9 | 0.88 (0.83, 0.92) | 72.2 | <0.001 |  |
|  | Cycling | | | 6 | 0.80 (0.75, 0.86) | 34.5 | 0.18 |  |
|  | Communing  (Walking and cycling) | | | 1 | 0.91 (0.84, 0.99) | - | - |  |
|  | Running | | | 1 | 0.55 (0.34, 0.89) | - | - |  |
|  | Jogging | | | 1 | 0.69 (0.33, 1.48) |  |  |  |
|  | Stairs climbing | | | 1 | 1.01 (0.69, 1.47) | - | - |  |
|  | Domestic | | | 2 | 0.99 (0.98, 1.0) | 0.0 | 0.52 |  |
| Adjustment for confounders | Other type of PA | Yes | | 7 | 0.79 (0.72, 0.87) | 40.3 | 0.12 | 0.61 |
|  |  | No | | 33 | 0.82 (0.77, 0.87) | 92.9 | <0.001 |  |
|  | BMI | Yes | | 21 | 0.81 (0.76, 0.86) | 73.7 | <0.001 | 0.88 |
|  |  | No | | 19 | 0.82 (0.76, 0.88) | 93.7 | <0.001 |  |
|  | Alcohol | Yes | | 24 | 0.80 (0.75, 0.86) | 94.2 | <0.001 | 0.47 |
|  |  | No | | 16 | 0.83 (0.77, 0.89) | 57.1 | 0.002 |  |
|  | Dietary pattern/ indices | Yes | | 10 | 0.81 (0.71, 0.93) | 96.5 | <0.001 | 0.91 |
|  |  | No | | 29 | 0.82 (0.78, 0.86) | 65.8 | <0.001 |  |
|  |  | Not explained | | 1 | 0.72 (0.39, 1.32) | - | - |  |
|  | Diabetes | Yes | | 24 | 0.83 (0.79, 0.87) | 50.3 | 0.003 | 0.33 |
|  |  | No | | 16 | 0.79 (0.72, 0.86) | 96.1 | <0.001 |  |
|  | HTN | Yes | | 24 | 0.83 (0.78, 0.87) | 53.7 | 0.001 | 0.58 |
|  |  | No | | 16 | 0.80 (0.74, 0.88) | 96.1 | <0.001 |  |
|  | Hyperlipidemia/hypercholesterolemia | Yes | | 19 | 0.81 (0.77, 0.86) | 59.9 | <0.001 | 0.89 |
|  |  | No | | 21 | 0.82 (0.76, 0.88) | 94.9 | <0.001 |  |
| BMI, body mass index; CVD, cardiovascular disease; HR, hazard ratio; LTPA, leisure time physical activity; PA, physical activity  1 Three studies reported the result for males and females, separately  2Only two studies had moderate risk of bias when diet adjustment was considered, therefore, we ignored adjustment for diet  3 One studies reported the results for both sport and total LTPA  ^4^ p-value for between subgroup heterogeneity including all type of activities  ^5^ p-value for between subgroup heterogeneity excluding domestic activity | | | | | | | | |

Supplementary Table 15. Subgroup analysis of studies assessing relationship between leisure-time physical activity and CHD/IHD/MI incidence according to potential important factors, high vs. low analysis

| **Subgroup factor** | | | **N of study** | **HR (95%CI)** | ***I*^2^ (%)** | **P heterogeneity** | **P between**  **group** |
| --- | --- | --- | --- | --- | --- | --- | --- |
| All studies | - | | 38 | 0.83(0.79, 0.87) | 75.6 | <0.000 | - |
| Sex^1^ | Male | | 19 | 0.83 (0.77, 0.90) | 64.6 | <0.001 | 1.0 |
|  | Female | | 15 | 0.83 (0.76, 0.91) | 63.0 | 0.001 |  |
|  | Both | | 12 | 0.83 (0.74, 0.93) | 85.4 | 0.12 |  |
| Risk of bias | Moderate | | 3 | 0.84 (0.69, 1.04) | 75.8 | 0.02 | 0.84 |
|  | Serious | | 35 | 0.83 (0.78, 0.87) | 76.2 | <0.001 |  |
| Rob2 | Moderate | | 11 | 0.86 (0.79, 0.92) | 59.4 | 0.006 | 0.39 |
|  | Serious | | 27 | 0.82 (0.77, 0.87) | 78.1 | <0.001 |  |
| Follow-up duration | ≤13 year | | 29 | 0.81 (0.76, 0.86) | 80.1 | <0.001 | 0.02 |
|  | >13 year | | 9 | 0.89 (0.85, 0.93) | 0.0 | 0.72 |  |
| Number of cases | ≤500 | | 14 | 0.80 (0.70, 0.93) | 69.8 | <0.001 | 0.74 |
|  | 501-1000 | | 8 | 0.81 (0.75, 0.88) | 13.8 | 0.32 |  |
|  | >1000 | | 16 | 0.84 (0.79, 0.90) | 84.2 | <0.001 |  |
| Geographical location | Europe | | 26 | 0.85 (0.81, 0.90) | 58.0 | <0.001 | 0.06 |
|  | USA | | 9 | 0.78 (0.71, 0.87) | 83.9 | <0.001 |  |
|  | East Asia | | 2 | 0.93 (0.86, 1.01) | 0.0 | 0.38 |  |
|  | Australia | | 1 | 0.82 (0.70, 0.96) | 0.0 | - |  |
| Time of PA assessment | Baseline | | 26 | 0.82 (0.76, 0.88) | 72.5 | <0.001 | 0.43 |
|  | Updated | | 12 | 0.85 (0.80, 0.90) | 71.1 | <0.001 |  |
| Exposure assessment | validated | | 24 | 0.83 (0.78, 0.88) | 60.9 | <0.001 | 0.73 |
|  | Non-validated | | 14 | 0.84 (0.77, 0.93) | 85.2 | <0.001 |  |
| Outcome assessment | Self-reported | | 3 | 0.94 (0.72, 0.12) | 79.6 | 0.007 | 0.34 |
|  | Medical record | | 35 | 0.82 (0.78, 0.87) | 75.9 | <0.001 |  |
| Adjustment for | Other type of PA | Yes | 10 | 0.80 (0.70, 0.90) | 71.7 | <0.001 | 0.49 |
|  |  | No | 28 | 0.84 (0.79, 0.88) | 76.8 | <0.001 |  |
|  | BMI | Yes | 27 | 0.83 (0.77, 0.89) | 80.3 | <0.001 | 0.66 |
|  |  | No | 11 | 0.85 (0.80, 0.90) | 34.3 | 0.12 | 0.12 |
|  | Alcohol | Yes | 28 | 0.83 (0.79, 0.88) | 80.7 | <0.001 | 0.69 |
|  |  | No | 10 | 0.81 (0.74, 0.89) | 19.9 | 0.26 |  |
|  | Diabetes | Yes | 23 | 0.83 (0.78, 0.89) | 59.8 | <0.001 | 0.77 |
|  |  | No | 15 | 0.82 (0.76, 0.89) | 85.5 | <0.001 |  |
|  | HTN | Yes | 26 | 0.82 (0.77, 0.87) | 60.1 | <0.001 | 0.67 |
|  |  | No | 12 | 0.84 (0.77, 0.92) | 87.6 | <0.001 |  |
|  | Hyperlipidemia/Cholesterol | Yes | 20 | 0.82 (0.76, 0.88) | 61.6 | <0.001 | 0.58 |
|  |  | No | 18 | 0.84 (0.78, 0.91) | 83.3 | <0.001 |  |
|  | Dietary pattern/ indices | Yes | 6 | 0.83 (0.68, 1.01) | 90.1 | <0.001 | 0.94 |
|  |  | No | 31 | 0.83 (.080, 0.87) | 60.4 | <0.001 |  |
| BMI, body mass index; CHD, coronary heart disease; HR, hazard ratio; IHD, ischemic heart; MI, myocardial infarction; PA, physical activity  ^1^Eight studies reported the result for males and females, separately  ^2^ One study adjusted for diet but didn’t explain which dietary factors was included so it was excluded from the subgroup analysis by adjustment for dietary factors | | | | | | | |

Supplementary Table 16. Subgroup analysis of studies assessing relationship between leisure-time physical activity and stroke incidence according to the potential important factors, high vs. low analysis

| **Subgroup factor** | | | **N of study** | **HR (95%CI)** | ***I*^2^ (%)** | **P heterogeneity** | **P between**  **group** |
| --- | --- | --- | --- | --- | --- | --- | --- |
| All studies | - | | 30 | 0.84 (0.79, 0.89) | 66.7 | <0.001 | - |
| Sex^1^ | Male | | 9 | 0.87 (0.79, 0.95) | 6.2 | 0.38 | 0.88 |
|  | Female | | 10 | 0.85 (0.79, 0.91) | 58.7 | 0.01 |  |
|  | Both | | 13 | 0.84 (0.75, 0.93) | 78.5 | <0.001 |  |
| Risk of bias^2^ | Moderate | | 6 | 0.90 (0.81, 1.01) | 61.3 | 0.02 | 0.15 |
|  | Serious | | 24 | 0.82 (0.77, 0.87) | 65.7 | <0.001 |  |
| Follow-up duration | ≤13 year | | 17 | 0.82 (0.76, 0.89) | 67.6 | <0.001 | 0.50 |
|  | >13 year | | 13 | 0.86 (0.79, 0.92) | 60.7 | 0.002 |  |
| Number of cases | ≤500 | | 11 | 0.81 (0.68, 0.97) | 61.4 | 0.004 | 0.70 |
|  | 501-1000 | | 5 | 0.81 (0.75, 0.88) | 0.0 | 0.42 |  |
|  | >1000 | | 14 | 0.85 (0.79, 0.91) | 76.8 | <0.001 |  |
| Geographical location | Europe | | 14 | 0.85 (0.78, 0.92) | 63.1 | 0.001 | 0.71 |
|  | USA | | 13 | 0.82 (0.75, 0.90) | 71.0 | <0.001 |  |
|  | East Asia | | 2 | 0.90 (0.69, 1.18) | 66.3 | 0.09 |  |
|  | Australia | | 1 | 0.92 (0.76, 1.11) | - | - |  |
| PA assessment | Validated | | 15 | 0.87 (0.81, 0.93) | 56.3 | 0.004 | 0.24 |
|  | Non-Validated | | 15 | 0.81 (0.75, 0.88) | 69.6 | <0.001 |  |
| Outcome assessment | Medical record/  Doctor diagnosed | | 26 | 0.85 (0.80, 0.90) | 69.3 | <0.001 | 0.05 |
|  | Self-reported | | 4 | 0.76 (0.69, 0.84) | 0.0 | 0.94 |  |
| Time of PA assessment | Baseline | | 21 | 0.82 (0.76, 0.89) | 71.6 | <0.001 | 0.41 |
|  | Repeated | | 9 | 0.86 (0.81, 0.91) | 40.4 | 0.10 |  |
| Type of stroke2 | Ischemic | | 8 | 0.84 (0.74, 0.95) | 82.6 | <0.001 | 0.08 |
|  | TIA | | 3 | 0.69 (0.56, 0.84) | 0.0 | 0.78 |  |
|  | Hemorrhagic | | 6 | 0.90 (0.79, 1.04) | 0.0 | 0.43 |  |
|  |  | |  |  |  |  |  |
| Adjustment for | Other type of PA | Yes | 3 | 0.82 (0.66, 1.02) | 26.1 | 0.26 | 0.87 |
|  |  | No | 27 | 0.84 (0.79, 0.89) | 69.0 | <0.001 |  |
|  | BMI | Yes | 18 | 0.83 (0.76, 0.90) | 70.7 | <0.001 | 0.58 |
|  |  | No | 12 | 0.85 (0.79, 0.92) | 57.2 | 0.007 |  |
|  | Alcohol | Yes | 23 | 0.85 (0.80, 0.90) | 70.3 | <0.001 | 0.43 |
|  |  | No | 7 | 0.78 (0.65, 0.94) | 53.2 | 0.05 |  |
|  | Diabetes | Yes | 19 | 0.87 (0.82, 0.93) | 53.3 | 0.003 | 0.04 |
|  |  | No | 11 | 0.77 (0.70, 0.85) | 76.7 | <0.001 |  |
|  | HTN | Yes | 19 | 0.86 (0.80, 0.92) | 60.6 | <0.001 | 0.37 |
|  |  | No | 11 | 0.81 (0.74, 0.89) | 75.1 | <0.001 |  |
|  | Hyperlipidemia/Cholesterol | Yes | 16 | 0.85 (0.79, 0.92) | 60.6 | 0.001 | 0.67 |
|  |  | No | 14 | 0.83 (0.76, 0.90) | 73.3 | <0.001 |  |
|  | Dietary pattern/ indices | Yes | 6 | 0.84 (0.73, 0.98) | 85.8 | <0.001 | 0.92 |
|  |  | No | 24 | 0.84 (0.79, 0.89) | 55.4 | 0.001 |  |
| BMI, body mass index; CI, confidence interval; HR, hazard ratio; TIA, transient ischemic attack; PA, physical activity  ^1^ Two studies reported the result for males and females, separately  ^2^Only two studies had moderate risk of bias when adjustment for diet was considered, therefore, we ignored adjustment for diet. | | | | | | | |

Supplementary Table 17. Subgroup analysis of studies assessing relationship between leisure-time physical activity and atrial fibrillation according to potential important factors, high vs. low analysis

| **Subgroup factor** | | | **N of study** | **HR (95%CI)** | ***I*^2^ (%)** | **P heterogeneity** | **P between**  **group** |
| --- | --- | --- | --- | --- | --- | --- | --- |
| All studies | - | | 12 | 1.01(0.93, 1.09) | 61.7 | 0.003 | - |
| Sex | Male | | 4 | 1.09 (1.0, 1.20) | 0.0 | 0.60 | 0.003 |
|  | Female | | 3 | 0.91 (0.86, 0.96) | 0.0 | 0.65 |  |
|  | Both | | 6 | 0.94 (0.87, 1.02) | 3.3 | 0.40 |  |
| Risk of bias^1^ | Moderate | | 9 | 1.02 (0.94, 1.12) | 51.7 | 0.04 | 0.06 |
|  | Serious | | 3 | 0.91 (0.84, 0.99) | 0.0 | 0.55 |  |
| Follow-up duration | ≤13 year | | 8 | 0.98 (0.91, 1.05) | 50.5 | 0.05 | 0.86 |
|  | >13 year | | 4 | 0.99 (0.85, 1.15) | 33.7 | 0.21 |  |
| Number of cases | ≤500 | | 4 | 0.96 (0.77, 1.20) | 23.7 | 0.27 | 0.85 |
|  | >500 | | 8 | 0.98 (0.92, 1.06) | 54.1 | 0.03 |  |
| Geographical location | Europe | | 5 | 1.02 (0.94, 1.11) | 0.0 | 0.55 |  |
|  | USA | | 5 | 1.00 (0.87, 1.15) | 63.9 | 0.03 | 0.20 |
|  | Australia | | 1 | 0.80 (0.62, 1.03) | - | - |  |
|  | Korea | | 1 | 0.93 (0.84, 1.02) | - | - |  |
| PA assessment | Validated | | 11 | 1.01 (0.93, 1.09) | 47.0 | 0.05 | 0.13 |
|  | Non-Validated | | 2 | 0.90 (0.81, 1.01) | 15.5 | 0.28 |  |
| Outcome assessment | Medical record/  Doctor diagnosed | | 10 | 1.00 (0.93, 1.08) | 33.0 | 0.14 | 0.04 |
|  | Self-reported | | 2 | 0.91 (0.86, 0.96) | 0.0 | 0.36 |  |
| Time of PA assessment | Baseline | | 9 | 0.97 (0.91, 1.03) | 0.0 | 0.48 | 0.64 |
|  | Repeated | | 3 | 1.02 (0.84, 1.23) | 81.8 | 0.004 |  |
| Adjustment for | Other type of PA | Yes | 1 | 1.05 (0.87, 1.27) | - | - | 0.47 |
|  |  | No | 11 | 0.97 (0.91, 1.04) | 44.8 | 0.05 |  |
|  | BMI | Yes | 12 | 0.98 (0.92, 1.05) | 42.7 | 0.06 | - |
|  |  | No | 0 | - | - | - |  |
|  | Alcohol | Yes | 11 | 0.99 (0.93, 1.06) | 42.4 | 0.07 | 0.11 |
|  |  | No | 1 | 0.80 (0.62, 1.03) | - | - |  |
|  | Diabetes | Yes | 10 | 1.00 (0.93, 1.08) | 47.1 | 0.05 | 0.10 |
|  |  | No | 2 | 0.87 (0.76, 1.01) | 0.0 | 0.41 |  |
|  | Hypertension | Yes | 8 | 0.99 (0.90, 1.10) | 57.7 | 0.02 | 0.43 |
|  |  | No | 4 | 0.95 (0.88, 1.02) | 0.0 | 0.46 |  |
|  | Hyperlipidemia/  cholesterol | Yes | 7 | 1.00 (0.91, 1.10) | 58.4 | 0.03 | 0.63 |
|  |  | No | 5 | 0.97 (0.88, 1.06) | 9.2 | 0.35 |  |
|  | Dietary pattern/ indices | Yes | 1 | 1.05 (0.87, 1.27) | - | - | 0.47 |
|  |  | No | 9 | 0.97 (0.91, 1.04) | 44.8 | 0.05 |  |
| HR, hazard ratio; CI, confidence interval  ^1^ Only one study had a moderate risk of bias when diet was considered, therefore, we ignored adjustment for diet. | | | | | | | |

Supplementary Table 18. Subgroup analysis of studies assessing relationship between occupational physical activity and CVD according to potential important factors, high vs. low analysis

| **Subgroup factor** | | | **N of study** | **HR (95%CI)** | ***I*^2^ (%)** | **P heterogeneity** | **P between**  **group** |
| --- | --- | --- | --- | --- | --- | --- | --- |
| All studies |  | | 7 | 1.01 (0.77, 1.32) | 88.4 | <0.001 | - |
| Sex | Male | | 1 | 0.80 (0.47, 1.37) | - | - | 0.40 |
|  | Female | | 0 | - | - | - |  |
|  | Both | | 6 | 1.04 (0.77, 1.40) | 90.3 | <0.001 |  |
| Risk of bias^1^ | Moderate | | 2 | 1.14 (0.52, 2.51) | 88.6 | 0.003 | 0.72 |
|  | Serious | | 5 | 0.97 (0.69, 1.37) | 90.1 | <0.001 |  |
| Follow-up duration | ≤13 year | | 4 | 0.90 (0.66, 1.23) | 92.0 | <0.001 | 0.25 |
|  | >13 year | | 3 | 1.30 (0.76, 2.23) | 60.4 | 0.08 |  |
| Number of cases | ≤500 | | 3 | 1.30 (0.76, 2.23) | 60.4 | 0.08 | 0.25 |
|  | 501-1000 | | 0 | - | - | - |  |
|  | >1000 | | 4 | 0.90 (0.66, 1.23) | 92.0 | <0.001 |  |
| Geographical location | Europe | | 5 | 0.98 (0.71, 1.36) | 76.8 | 0.002 | 0.77 |
|  | USA | | 0 | - | - | - |  |
|  | Australia | | 0 | - | - | - |  |
|  | East Asia | | 2 | 1.12 (0.50, 2.50) | 91.5 | 0.001 |  |
| PA assessment | Validated | | 5 | 1.00 (0.72, 1.41) | 65.9 | 0.02 | 0.98 |
|  | Non-Validated | | 2 | 1.01 (0.58, 1.75) | - | - |  |
| Outcome assessment | Medical record/  Doctor diagnosed | | 7 | 1.01 (0.77, 1.32) | 88.4 | <0.001 | - |
|  | Self-reported | | 0 | - | - | - |  |
| Time of PA assessment | Baseline | | 6 | 0.93 (0.71, 1.22) | 87.7 | <0.001 | 0.02 |
|  | Repeated | | 1 | 1.75 (1.10, 2.79) | - | - |  |
| Adjustment for | Other type of PA | Yes | 1 | 0.77 (0.75, 0.80) | - | - | 0.03 |
|  |  | No | 6 | 1.08 (0.79, 1.46) | 76.4 | 0.001 |  |
|  | BMI | Yes | 4 | 1.05 (0.73, 1.52) | 93.8 | <0.001 | 0.52 |
|  |  | No | 3 | 0.89 (0.63, 1.27) | 26.6 | 0.26 |  |
|  | Alcohol | Yes | 5 | 1.00 (0.73, 1.36) | 91.8 | <0.001 | 0.85 |
|  |  | No | 2 | 1.07 (0.53, 2.15) | 52.6 | 0.15 |  |
|  | Diabetes | Yes | 4 | 1.32 (1.00, 1.75) | 41.0 | 0.17 | <0.001 |
|  |  | No | 3 | 0.77 (0.75, 0.80) | 0.0 | 0.99 |  |
|  | Hypertension | Yes | 5 | 1.15 (0.77, 1.70) | 92.3 | <0.001 | 0.10 |
|  |  | No | 2 | 0.78 (0.63, 0.97) | 0.0 | 0.96 |  |
|  | Hyperlipidemia/  cholesterol | Yes | 3 | 1.28 (0.91, 1.80) | 58.5 | 0.09 | 0.006 |
|  |  | No | 4 | 0.78 (0.71, 0.85) | 10.8 | 0.34 |  |
|  | Dietary pattern/ indices | Yes | 0 | - | - | - | - |
|  |  | No | 6 | 1.04 (0.77, 1.40) | 90.3 | <0.001 |  |
| BMI, body mass index; CHD, coronary heart disease; CI, confidence interval; CVD, cardiovascular disease; HR, hazard ratio; PA, physical activity  ^1^Only one study had a moderate Risk of bias for OPA, therefore, we ignored adjustment for diet | | | | | | | |

Supplementary Table 19. Subgroup analysis of studies assessing relationship between occupational physical activity and CHD according to potential important factors, high vs. low analysis

| **Subgroup factor** | | | **N of study** | **HR (95%CI)** | ***I*^2^ (%)** | **P heterogeneity** | **P between**  **group** |
| --- | --- | --- | --- | --- | --- | --- | --- |
| All studies |  | | 12 | 0.90 (0.78, 1.04) | 87.5 | <0.001 | - |
| Sex | Male | | 9 | 0.96(0.81, 1.15) | 85.3 | <0.001 | 0.07 |
|  | Female | | 7 | 0.87(0.64, 1.17) | 73.6 | 0.001 |  |
|  | Both | | 2 | 0.70(0.60, 0.82) | 16.4 | 0.02 |  |
| Risk of bias^1^ | Moderate | | 5 | 0.99 (0.78, 1.26) | 92.1 | <0.001 | 0.30 |
|  | Serious | | 7 | 0.84 (0.69, 1.03) | 79.3 | <0.001 |  |
| Follow-up duration | ≤13 year | | 5 | 0.82 (0.62, 1.07) | 79.1 | 0.001 | 0.33 |
|  | >13 year | | 7 | 0.96 (0.80, 1.15) | 88.9 | <0.001 |  |
| Number of cases | ≤500 | | 9 | 0.85 (0.71, 1.02) | 89.9 | <0.001 | 0.16 |
|  | >500 | | 3 | 1.04 (0.84, 1.29) | 64.5 | 0.06 |  |
|  |  | |  |  |  |  |  |
|  |  | |  |  |  |  |  |
|  |  | |  |  |  |  |  |
|  |  | |  |  |  |  |  |
| Geographical location | Europe | | 11 | 0.93 (0.81, 1.07) | 81.0 | <0.001 | <0.001 |
|  | USA | | 0 | - | - | - | - |
|  | Australia | | 0 | - | - | - | - |
|  | East Asia | | 1 | 0.88 (0.75, 1.03) | - | - |  |
| PA assessment | Validated | | 6 | 0.96 (0.78, 1.18) | 75.6 | 0.001 | 0.43 |
|  | Non-validated | | 6 | 0.86 (0.72, 1.02) | 83.5 | <0.001 |  |
| Outcome assessment | Medical record/  Doctor diagnosed | | 12 | 0.90 (0.78, 1.04) | 87.5 | <0.001 | - |
|  | Self-reported | | 0 | - | - | - |  |
| Time of PA assessment | Baseline | | 11 | 0.88 (0.75, 1.03) | 88.0 | <0.001 | 0.05 |
|  | Repeated | | 1 | 1.15 (0.92, 1.43) | - | - |  |
| Adjustment for | Other PA | Yes | 9 | 0.93 (0.79, 1.10) | 89.2 | <0.001 | 0.50 |
|  |  | No | 3 | 0.78(0.47, 1.29) | 84.8 | 0.001 |  |
|  | Alcohol | Yes | 8 | 0.97 (0.81, 1.15) | 90.8 | <0.001 | 0.10 |
|  |  | No | 4 | 0.75 (0.58, 0.96) | 56.1 | 0.08 |  |
|  | BMI | Yes | 10 | 0.91 (0.78, 1.06) | 89.8 | <0.001 | 0.66 |
|  |  | No | 2 | 0.84(0.59, 1.18) | 0.0 | 0.80 |  |
|  | Diabetes | Yes | 8 | 1.01 (0.86, 1.18) | 81.1 | <0.001 | 0.006 |
|  |  | No | 4 | 0.72 (0.60, 0.87) | 65.8 | 0.03 |  |
|  | HTN | Yes | 9 | 0.88 (0.74, 1.06) | 90.4 | <0.001 | 0.57 |
|  |  | No | 3 | 0.96 (0.78, 1.18) | 48.7 | 0.14 |  |
|  | Hyperlipidemia/cholesterol | Yes | 7 | 0.99 (0.83, 1.17) | 82.7 | <0.001 | 0.15 |
|  |  | No | 5 | 0.80 (0.63, 1.00) | 82.5 | <0.001 |  |
|  | Dietary pattern/ indices | Yes | 3 | 0.69 (0.63, 0.76) | 0.0 | 0.51 | 0.001 |
|  |  | No | 9 | 0.94 (0.81, 1.10) | 84.7 | <0.001 |  |

BMI, body mass index; CHD, coronary heart disease; CI, confidence interval; CVD, cardiovascular disease; HR, hazard ratio; PA, physical activity

^1^Only one study had a moderate Risk of bias for OPA, therefore, we ignored adjustment for diet

^2^ One study adjusted for diet but didn’t explain which dietary factors was included so it was excluded from the subgroup analysis by adjustment for dietary factors

Supplementary Table 20. Subgroup analysis of studies assessing relationship between occupational physical activity and stroke according to potential important factors, high vs. low analysis

| **Subgroup factor** | | | **N of study** | **HR (95%CI)** | ***I*^2^ (%)** | **P heterogeneity** | **P between**  **group** |
| --- | --- | --- | --- | --- | --- | --- | --- |
| All studies |  |  | 6 | 0.91 (0.80, 1.04) | 80.2 | <0.001 | - |
| Sex | Male | | 0 | - | - | - | 0.22 |
|  | Female | | 2 | 0.82 (0.74, 0.92) | 0.0 | 0.35 |  |
|  | Both | | 4 | 0.88 (0.76, 1.02) | 84.6 | <0.001 |  |
| Risk of bias^1^ | Moderate | | 1 | 0.80 (0.49, 1.30) | - | - | 0.61 |
|  | Serious | | 5 | 0.91 (0.79, 1.05) | 80.3 | <0.001 |  |
| Follow-up duration | ≤13 year | | 3 | 0.88 (0.68, 1.15) | 68.3 | 0.04 | 0.63 |
|  | >13 year | | 3 | 0.95 (0.84, 1.07) | 41.9 | 0.18 |  |
| Number of cases | ≤500 | | 2 | 0.88 (0.67, 1.16) | 0.0 | 0.64 | 0.76 |
|  | >500 | | 4 | 0.92 (0.79, 1.08) | 87.8 | <0.001 |  |
| Geographical location | Europe | | 4 | 0.94 (0.85, 1.04) | 21.6 | 0.28 | <0.001 |
|  | USA | | 1 | 1.14 (0.85, 1.53) | - | - |  |
|  | Australia | | 0 | - | - | - |  |
|  | East Asia | | 1 | 0.78 (0.75, 0.81) | - | - |  |
| PA assessment | Validated | | 1 | 0.80 (0.49, 1.30) | - | - | 0.61 |
|  | Non-validated | | 5 | 0.91 (0.79, 1.05) | 80.3 | <0.001 |  |
| Outcome assessment | Medical record/  Doctor diagnosed | | 5 | 0.93 (0.76, 1.13) | 81.1 | <0.001 | 0.71 |
|  | Self-reported | | 1 | 0.89 (0.81, 0.98) | - | - |  |
| Time of PA assessment | Baseline | | 6 | 0.91 (0.80, 1.04) | 80.2 | <0.001 | - |
|  | Repeated | | 0 | - | - | - |  |
| Adjustment for | PA | Yes | 5 | 0.87 (0.77, 0.98) | 68.5 | 0.01 | 0.05 |
|  |  | No | 1 | 1.05 (0.91, 1.22) | - | - |  |
|  | Alcohol | Yes | 4 | 0.87 (0.76, 0.99) | 75.1 | 0.007 | 0.08 |
|  |  | No | 2 | 1.03 (0.90, 1.18) | 0.0 | 0.48 |  |
|  | BMI | Yes | 6 | 0.91 (0.80, 1.04) | 80.2 | <0.001 | - |
|  |  | No | 0 | - | - | - |  |
|  | Diabetes | Yes | 3 | 0.94 (0.82, 1.08) | 47.7 | 0.15 | 0.78 |
|  |  | No | 3 | 0.90 (0.71, 1.16) | 72.0 | 0.03 |  |
|  | HTN | Yes | 5 | 0.91 (0.79, 1.06) | 83.8 | < 0.001 | 0.97 |
|  |  | No | 1 | 0.92 (0.66, 1.29) | - | - |  |
|  | Hyperlipidemia/  cholesterol | Yes | 3 | 0.94 (0.82, 1.08) | 47.7 | 0.15 | 0.78 |
|  |  | No | 3 | 0.90 (0.71, 1.16) | 72.0 | 0.03 |  |
|  | Dietary pattern/ indices | Yes | 3 | 0.93 (0.82, 1.05) | 19.0 | 0.29 | 0.69 |
|  |  | No | 3 | 0.88 (0.69, 1.12) | 68.5 | 0.01 |  |
| BMI, body mass index; CHD, coronary heart disease; CI, confidence interval; CVD, cardiovascular disease; HR, hazard ratio; PA, physical activity  ^1^Only one study had a moderate Risk of bias for OPA, therefore, we ignored adjustment for diet | | | | | | | |

**Supplementary Table 21:** Results of grade assessment for relationship between leisure time physical activity and CVD, CHD, stroke, and AF in general population

| **Certainty assessment** | | | | | | | **№ of patients** | **Effect** | | **Certainty** | **Importance** |
| --- | --- | --- | --- | --- | --- | --- | --- | --- | --- | --- | --- |
| **№ of studies** | **Study design** | **Risk of bias** | **Inconsistency** | **Indirectness** | **Imprecision** | **Other considerations** |  | **Relative (95% CI)** | **Absolute (95% CI)** |  |  |
| **Coronary heart disease (follow-up: median 3.15 -34 years)** | | | | | | | | | | | |
| 39 | observational studies | serious^a^ | not serious^b^ | not serious | Serious^c^ | dose response gradient | 120048/ 2594495 (4.6%) | **HR 0.83** (0.79 to 0.87) | **0.78 fewer per 100** (from 0.97 fewer to 0.60 fewer) | ⨁⨁⨁◯ Moderate | CRITICAL |
| **Cardiovascular diseases (follow-up: median 3.3-30 years)** | | | | | | | | | | | |
| 40 | observational studies | very serious^d^ | not serious^e^ | not serious | not serious | dose response gradient | 290811/ 2,876,417 (10.1%) | **HR 0.81** (0.77 to 0.86) | **1.92 fewer per 100** (from 2.32 fewer to 1.41 fewer) | ⨁⨁⨁◯ Moderate | CRITICAL |
| **Stroke (follow-up: median 3.3-33 years)** | | | | | | | | | | | |
| 31 | observational studies | serious^f^ | not serious^g^ | not serious | Serious^c^ | dose response gradient | 77215/ 2595295  (3.0%) | **HR 0.84** (0.79 to 0.89) | **0.48 fewer per 100** (from 0.63 fewer to 0.33 fewer) | ⨁⨁⨁◯ Moderate | CRITICAL |
| **Atrial fibrillation (follow-up: median 4-20.3 years)** | | | | | | | | | | | |
| 12 | observational studies | serious^h^ | not serious^i^ | not serious | Serious^c^ | dose response gradient | 24642/ 764,640 (3.2%) | **HR 0.98** (0.92 to 1.05) | **0.06 fewer per 100** (from 0.26 fewer to 0.16 more) | ⨁⨁⨁◯ Moderate | CRITICAL |

**AF,** atrial fibrillation; **CHD,** coronary heart disease; **CI:** confidence interval; **CVD,** cardiovascular diseases; **HR:** hazard Ratio

#### Explanations

**CHD**

a. Although 27 studies (35, when ignored diet) were at high risk of bias we downgrade only one level since the result in the subgroup of moderate risk of bias was the same as the overall result both when adjustment for diet was ignored [HR=0.86 (95%CI: 0.79, 0.92), n=11], and when adjustment for diet was considered [HR=0.84 (95%CI: 0.69, 1.04), n=3].

b. Although I2 was > 50%, we did not downgrade for inconsistency since in one subgroup with the follow-up duration of > 13 year [HR=0.89 (95%CI: 0.85, 0.93), I2=0.0%; n=9] and case number of 501-1000 [HR=0.81 (95%CI: 0.75, 0.88), I2=13.8%; n=8] heterogeneity was reduced. Not downgraded

c. Downgraded 1 level, since 95%CIs of RD were not larger than the MID threshold for important benefit (-1% in absolute effect). Downgraded.

**CVD:**

d. Downgraded 2 levels since 34 studies (89.5%, when ignored adjustment for diet) were at serious risk of bias

e. Although I2 was > 50%, we did not downgrade for inconsistency since in one subgroup conducted in males [HR=0.77 (95%CI: 0.71, 0.84), I2=29.2%; n=9] and East Asia studies [HR=0.77 (95%CI: 0.71, 0.84), I2=36.1%; n=4] heterogeneity was reduced. Not downgraded

**Stroke:**

f. Downgraded 2 level, since 24 studies (80.0%) were at serious risk of bias (we ignored adjustment for diet)

g. Although I2 was > 50%, we did not downgrade for inconsistency since in the subgroup of studies with male participants [HR=0.87 (95%CI: 0.79, 0.95), I2=6.2%; n=9] and studies that assessed PA at the follow-up, in addition to follow-up, [HR=0.81 (95%CI: 0.81, 0.91), I2=40.4%; n=9] heterogeneity was reduced. Not downgraded

**AF**

h. Seven studies (70.0%) were at moderate risk of bias (when adjustment for diet was ignored, only one study adjusted for diet) so only one level was downgraded for the residual confounding

i. Although I2 was 52.5 %, we did not downgrade for inconsistency since in the subgroup of studies with a follow-up duration of >13 years [HR=099 (95%CI: 0.85, 1.15), I2=33.7%; n=4], heterogeneity was reduced. Not downgraded

**Supplementary Table 22:** Results of grade assessment for relationship between occupational physical activity and CVD, CHD, stroke, and AF in general population

| **Certainty assessment** | | | | | | | **№ of Case/ patients** | **Effect** | | **Certainty** | **Importance** |
| --- | --- | --- | --- | --- | --- | --- | --- | --- | --- | --- | --- |
| **№ of studies** | **Study design** | **Risk of bias** | **Inconsistency** | **Indirectness** | **Imprecision** | **Other considerations** |  | **Relative (95% CI)** | **Absolute (95% CI)** |  |  |
| **Coronary heart diseases (follow-up: median 3.15-19.56 years; assessed with: MET-hour/week)** | | | | | | | | | | | |
| 12 | observational studies | serious^a^ | serious^b^ | serious^c^ | serious^d^ | none | 14122/ 630,236 (2.2%) | **HR 0.90** (0.78 to 1.04) | **0.22 fewer per 100** (from 0.48 fewer to 0.09 more) | ⨁◯◯◯ Very low | Critical |
| **Total cardiovascular diseases (follow-up: median 6.8- 16.8 years; assessed with: Met-hour/week)** | | | | | | | | | | | |
| 7 | observational studies | very serious^e^ | very serious^f^ | not serious | very serious^g^ | none | 46543/ 733300 (6.3%) | **HR 1.01** (0.77 to 1.32) | **0.06 more per 100** (from 1.45 fewer to 2.02 more) | ⨁◯◯◯ Very low | Critical |
| **Stroke (follow-up: median 5.7-44 years; assessed with: Met-hour/week)** | | | | | | | | | | | |
| 5 | observational studies | very serious^h^ | serious^i^ | not serious | serious^d^ | none | 37342/ 625347  (5.9%) | **HR 0.91** (0.80 to 1.04) | **0.53 fewer per 100** (from 1.18 fewer to 0.24 more) | ⨁◯◯◯ Very low | Critical |
| **Atrial fibrillation (follow-up: median 17-20.3 years; assessed with: MET-hour/week)** | | | | | | | | | | | |
| 2 | observational studies | very serious^g^ | not serious | not serious | very serious^i^ | none | 5035 /53708 (9.4%) | **HR 1.17** (0.99 to 1.38) | **1.6 more per 100** (from 0.94 fewer to 3.57 more) | ⨁◯◯◯ Very low | Critical |

**AF,** atrial fibrillation; **CHD,** coronary heart disease; **CI:** confidence interval; **CVD,** cardiovascular diseases; **HR:** hazard Ratio

#### Explanations

#### CHD

a. Downgraded one level since 79.6% of pooled person year were from studies with a moderate risk of bias (when diet was ignored, and 74% when diet was considerd.

b. Downgraded one level since I2=88.1, and the source of heterogeneity was not found

c. Downgraded one level, since 95% CIs of RD included -1 (1 per 100 fewer)

d. All the studies were at serious risk of bias

**CVD**

e. Since *I*^2^= 88.4%

f. Downgraded 2 levels, since 95% CIs of RD included both +1 and -1 (1.13 fewer to 3.38 more)

g. Downgraded 2 levels, since 5 studies were at serious risk of bias

**Stroke**

h. Downgraded one level since *I*^2^=68.5%

i. Downgraded 2 levels, since 95% CIs of RD included both +1 and -1 (0.94 fewer to 3.57 more)

1. Ma Y, Wang YJ, Chen BR, Shi HJ, Wang H, Khurwolah MR, et al. Study on association of working hours and occupational physical activity with the occurrence of coronary heart disease in a Chinese population. Plos One. 2017;12(10).

2. Wang CL, De Roos AJ, Fujishiro K, Allison MA, Wallace R, Seguin RA, et al. Occupational Physical Activity and Coronary Heart Disease in Women's Health Initiative Observational Study. Journals of Gerontology Series a-Biological Sciences and Medical Sciences. 2019;74(12):1952-8.

3. Maessen MF, Verbeek AL, Bakker EA, Thompson PD, Hopman MT, Eijsvogels TM, editors. Lifelong exercise patterns and cardiovascular health. Mayo Clinic Proceedings; 2016: Elsevier.

4. Fortuin-De Smidt MC, Sewe MO, Lassale C, Weiderpass E, Andersson J, Huerta JM, et al. Physical activity attenuates but does not eliminate coronary heart disease risk amongst adults with risk factors: EPIC-CVD case-cohort study. European Journal of Preventive Cardiology. 2022;29(12):1618-29.

5. Fortuin-de Smidt M, Bergman F, Grönlund C, Hult A, Norberg M, Wennberg M, et al. Early adulthood exercise capacity, but not muscle strength, associates with subclinical atherosclerosis 40 years later in Swedish men. Eur J Prev Cardiol. 2023;30(5):407-15.

6. Andresdottir MB, Sigurdsson G, Sigvaldason H, Gudnason V. Fifteen percent of myocardial infarctions and coronary revascularizations explained by family history unrelated to conventional risk factors. The Reykjavik Cohort Study. Eur Heart J. 2002;23(21):1655-63.

7. Sarajlic P, Wolk A, Bäck M, Larsson SC. Physical activity does not reduce aortic valve stenosis incidence. Circulation Journal. 2018;82(9):2372-4.

8. López-Laguna N, Martínez-González MA, Toledo E, Babio N, Sorlí JV, Ros E, et al. Risk of peripheral artery disease according to a healthy lifestyle score: the PREDIMED study. Atherosclerosis. 2018;275:133-40.

9. Grenon SM, Cohen BE, Smolderen K, Vittinghoff E, Whooley MA, Hiramoto J. Peripheral arterial disease, gender, and depression in the Heart and Soul Study. Journal of vascular surgery. 2014;60(2):396-403.

10. Ahmadi-Abhari S, Sabia S, Shipley MJ, Kivimäki M, Singh-Manoux A, Tabak A, et al. Physical Activity, Sedentary Behavior, and Long-Term Changes in Aortic Stiffness: The Whitehall II Study. J Am Heart Assoc. 2017;6(8).

11. Defilippi CR, De Lemos JA, Tkaczuk AT, Christenson RH, Carnethon MR, Siscovick DS, et al. Physical activity, change in biomarkers of myocardial stress and injury, and subsequent heart failure risk in older adults. Journal of the American College of Cardiology. 2012;60(24):2539-47.

12. Dhaliwal SS, Welborn TA, Howat PA. Recreational physical activity as an independent predictor of multivariable cardiovascular disease risk. PLoS One. 2013;8(12):e83435.

13. Pitanga FJG, Matos S, Almeida MdC, Barreto SM, Aquino EM. Leisure-time physical activity, but not commuting physical activity, is associated with cardiovascular risk among ELSA-Brasil participants. Arquivos Brasileiros de Cardiologia. 2018;110:36-43.

14. Aengevaeren VL, Mosterd A, Braber TL, Prakken NHJ, Doevendans PA, Grobbee DE, et al. Relationship Between Lifelong Exercise Volume and Coronary Atherosclerosis in Athletes. Circulation. 2017;136(2):138-48.

15. Sung KC, Hong YS, Lee JY, Lee SJ, Chang Y, Ryu S, et al. Physical activity and the progression of coronary artery calcification. Heart. 2021;107(21):1710-6.

16. Martinez-Gomez D, Esteban-Cornejo I, Lopez-Garcia E, García-Esquinas E, Sadarangani KP, Veiga OL, et al. Physical activity less than the recommended amount may prevent the onset of major biological risk factors for cardiovascular disease: A cohort study of 198 919 adults. British Journal of Sports Medicine. 2020;54(4):238-44.

17. Alves CPD, Crochemore-Silva I, Lima NP, Coenen P, Horta BL. Prospective Association of Occupational and Leisure-Time Physical Activity With Cardiovascular Risk Factors in Early Adulthood: Findings From Pelotas (Brazil) 1982 Birth Cohort. Journal of Physical Activity & Health. 2023.

18. Bierig SM, Arnold A, Einbinder LC, Armbrecht E, Burroughs T. Unrecognized Cardiovascular Abnormalities Detected Through a Community Cardiovascular Screening Program. Journal of Diagnostic Medical Sonography. 2020;36(3):234-42.

19. Artero EG, España-Romero V, Lee D-c, Sui X, Church TS, Lavie CJ, et al., editors. Ideal cardiovascular health and mortality: aerobics center longitudinal study. Mayo Clinic Proceedings; 2012: Elsevier.

20. Menotti A, Seccareccia F. Physical activity at work and job responsibility as risk factors for fatal coronary heart disease and other causes of death. Journal of epidemiology and community health. 1985;39(4):325-9.

21. Frimodt-Møller EK, Soliman EZ, Kizer JR, Vittinghoff E, Psaty BM, Biering-Sørensen T, et al. Lifestyle habits associated with cardiac conduction disease. Eur Heart J. 2023;44(12):1058-66.

22. Kim D, Seo J, Ha KH, Kim DJ. Maintaining Physical Activity Is Associated with Reduced Major Adverse Cardiovascular Events in People Newly Diagnosed with Diabetes. J Obes Metab Syndr. 2022.

23. Djousse L, Schubert P, Ho YL, Whitbourne SB, Cho K, Gaziano JM. Leisure time physical activity, sedentary behavior, and risk of cardiovascular disease and mortality among US Veterans. J Nov Physiother Phys Rehabil. 2021;8(2):33-9.

24. Faselis C, Kokkinos P, Tsimploulis A, Pittaras A, Myers J, Lavie CJ, et al. Exercise Capacity and Atrial Fibrillation Risk in Veterans: A Cohort Study. Mayo Clin Proc. 2016;91(5):558-66.

25. Kujala UM, Sarna S, Kaprio J, Tikkanen HO, Koskenvuo M. Natural selection to sports, later physical activity habits, and coronary heart disease. British journal of sports medicine. 2000;34(6):445-9.

26. Böhm M, Schumacher H, Werner C, Teo KK, Lonn EM, Mahfoud F, et al. Association between exercise frequency with renal and cardiovascular outcomes in diabetic and non-diabetic individuals at high cardiovascular risk. Cardiovascular diabetology. 2022;21(1):1-12.

27. Aengevaeren VL, Mosterd A, Bakker EA, Braber TL, Nathoe HM, Sharma S, et al. Exercise Volume Versus Intensity and the Progression of Coronary Atherosclerosis in Middle-Aged and Older Athletes: Findings From the MARC-2 Study. Circulation. 2023;147(13):993-1003.

28. Soares-Miranda L, Siscovick DS, Psaty BM, Longstreth WT, Mozaffarian D. Physical Activity and Risk of Coronary Heart Disease and Stroke in Older Adults The Cardiovascular Health Study. Circulation. 2016;133(2):147-55.

29. Autenrieth CS, Evenson KR, Yatsuya H, Shahar E, Baggett C, Rosamond WD. Association between physical activity and risk of stroke subtypes: the atherosclerosis risk in communities study. Neuroepidemiology. 2013;40(2):109-16.

30. Willey JZ, Moon YP, Paik MC, Boden-Albala B, Sacco RL, Elkind MSV. Physical activity and risk of ischemic stroke in the Northern Manhattan study. Neurology. 2009;73(21):1774-9.

31. Allesøe K, Holtermann A, Rugulies R, Aadahl M, Boyle E, Søgaard K. Does influence at work modify the relation between high occupational physical activity and risk of heart disease in women? Int Arch Occup Environ Health. 2017;90(5):433-42.

32. Chomistek AK, Chiuve SE, Eliassen AH, Mukamal KJ, Willett WC, Rimm EB. Healthy lifestyle in the primordial prevention of cardiovascular disease among young women. J Am Coll Cardiol. 2015;65(1):43-51.

33. Chomistek AK, Chasman DI, Cook NR, Rimm EB, Lee IM. Physical activity, genes for physical fitness, and risk of coronary heart disease. Med Sci Sports Exerc. 2013;45(4):691-7.

34. Lee I-M, Rexrode KM, Cook NR, Manson JE, Buring JE. Physical activity and coronary heart disease in women: Is no pain, no gain passé? Jama. 2001;285(11):1447-54.

35. Alvarez-Alvarez I, de Rojas JP, Fernandez-Montero A, Zazpe I, Ruiz-Canela M, Hidalgo-Santamaría M, et al. Strong inverse associations of Mediterranean diet, physical activity and their combination with cardiovascular disease: The Seguimiento Universidad de Navarra (SUN) cohort. European journal of preventive cardiology. 2018;25(11):1186-97.

36. Díaz-Gutiérrez J, Ruiz-Canela M, Gea A, Fernández-Montero A, Martínez-González MÁ. Association between a healthy lifestyle score and the risk of cardiovascular disease in the SUN cohort. Revista Española de Cardiología (English Edition). 2018;71(12):1001-9.

37. CONROY MB, COOK NR, MANSON JE, BURING JE, LEE I-M. Past Physical Activity, Current Physical Activity, and Risk of Coronary Heart Disease. Medicine & Science in Sports & Exercise. 2005;37(8):1251-6.

38. Ferrario MM, Veronesi G, Roncaioli M, Holtermann A, Krause N, Clays E, et al. Exploring the interplay between job strain and different domains of physical activity on the incidence of coronary heart disease in adult men. European Journal of Preventive Cardiology. 2019;26(17):1877-85.

39. Wannamethee G, Shaper A. Physical activity and stroke in British middle aged men. British Medical Journal. 1992;304(6827):597-601.

40. Wannamethee SG, Shaper AG, Alberti K. Physical activity, metabolic factors, and the incidence of coronary heart disease and type 2 diabetes. Archives of Internal Medicine. 2000;160(14):2108-16.

41. Ahmad T, Chasman DI, Mora S, Paré G, Cook NR, Buring JE, et al. The fat-mass and obesity-associated (FTO) gene, physical activity, and risk of incident cardiovascular events in white women. American heart journal. 2010;160(6):1163-9.

42. Lee I-M, Hennekens CH, Berger K, Buring JE, Manson JE. Exercise and risk of stroke in male physicians. Stroke. 1999;30(1):1-6.

43. Li TY, Rana JS, Manson JE, Willett WC, Stampfer MJ, Colditz GA, et al. Obesity as compared with physical activity in predicting risk of coronary heart disease in women. Circulation. 2006;113(4):499-506.

44. Shaper AG, Wannamethee G. PHYSICAL-ACTIVITY AND ISCHEMIC-HEART-DISEASE IN MIDDLE-AGED BRITISH MEN. British Heart Journal. 1991;66(5):384-94.

45. Shaper AG, Wannamethee G, Walker M. Physical activity, hypertension and risk of heart attack in men without evidence of ischaemic heart disease. J Hum Hypertens. 1994;8(1):3-10.

46. Bapat A, Zhang Y, Post WS, Guallar E, Soliman EZ, Heckbert SR, et al. Relation of Physical Activity and Incident Atrial Fibrillation (from the Multi-Ethnic Study of Atherosclerosis). Am J Cardiol. 2015;116(6):883-8.

47. Lee S-R, Choi E-K, Ahn H-J, Han K-D, Oh S, Lip GY. Association between clustering of unhealthy lifestyle factors and risk of new-onset atrial fibrillation: a nationwide population-based study. Scientific reports. 2020;10(1):1-9.

48. Su N, Kim Y, Won Y, editors. Association of Primary Hypertension and Risk of Cerebrovascular Diseases with Overweight and Physical Activity in Korean Women: A Longitudinal Study. Healthcare; 2021: MDPI.

49. Kurth T, Moore SC, Gaziano JM, Kase CS, Stampfer MJ, Berger K, et al. Healthy lifestyle and the risk of stroke in women. Archives of internal medicine. 2006;166(13):1403-9.

50. Mora S, Cook N, Buring JE, Ridker PM, Lee IM. Physical activity and reduced risk of cardiovascular events: potential mediating mechanisms. Circulation. 2007;116(19):2110-8.

51. Chiuve SE, Cook NR, Shay CM, Rexrode KM, Albert CM, Manson JE, et al. Lifestyle‐based prediction model for the prevention of CVD: the Healthy Heart Score. Journal of the American Heart Association. 2014;3(6):e000954.

52. Harmsen P, Rosengren A, Tsipogianni A, Wilhelmsen L. Risk factors for stroke in middle-aged men in Göteborg, Sweden. Stroke. 1990;21(2):223-9.

53. Lapidus L, Bengtsson C. Socioeconomic factors and physical activity in relation to cardiovascular disease and death. A 12 year follow up of participants in a population study of women in Gothenburg, Sweden. Br Heart J. 1986;55(3):295-301.

54. Evenson KR, Rosamond WD, Cai J, Toole JF, Hutchinson RG, Shahar E, et al. Physical activity and ischemic stroke risk. The atherosclerosis risk in communities study. Stroke. 1999;30(7):1333-9.

55. SALONEN J, PUSKA P, TUOMILEHTO J. PHYSICAL ACTIVITY AND RISK OF MYOCARDIAL INFARCTION, CEREBRAL STROKE AND DEATH: A LONGITUDINAL STUDY IN EASTERN FINLAND. American Journal of Epidemiology. 1982;115(4):526-37.

56. Cheung YK, Yu G, Wall MM, Sacco RL, Elkind MSV, Willey JZ. Patterns of leisure-time physical activity using multivariate finite mixture modeling and cardiovascular risk factors in the Northern Manhattan Study. Annals of Epidemiology. 2015;25(7):469-74.

57. Ahmad T, Chasman DI, Buring JE, Lee IM, Ridker PM, Everett BM. Physical activity modifies the effect of LPL, LIPC, and CETP polymorphisms on HDL-C levels and the risk of myocardial infarction in women of European ancestry. Circ Cardiovasc Genet. 2011;4(1):74-80.

58. Menotti A, Keys A, Blackburn H, Aravanis C, Dontas A, Fidanza F, et al. Twenty-year stroke mortality and prediction in twelve cohorts of the Seven Countries Study. International journal of epidemiology. 1990;19(2):309-15.

59. PAFFENBARGER RS, Jr., BRAND RJ, SHOLTZ RI, JUNG DL. ENERGY EXPENDITURE, CIGARETTE SMOKING, AND BLOOD PRESSURE LEVEL AS RELATED TO DEATH FROM SPECIFIC DISEASES1. American Journal of Epidemiology. 1978;108(1):12-8.

60. Panagiotakos DB, Pitsavos C, Crysohoou C, Menotti A, Skoumas J, Stefanadis C. Physical activity attenuates the risk of stroke in middle-age men with left ventricular hypertrophy: 40-Year follow-up (1961-2001) of the Corfu cohort (Seven Countries Study). Journal of the American College of Cardiology. 2004;43(5):15A-A.

61. Kubota Y, Evenson KR, Maclehose RF, Roetker NS, Joshu CE, Folsom AR. Physical Activity and Lifetime Risk of Cardiovascular Disease and Cancer. Med Sci Sports Exerc. 2017;49(8):1599-605.

62. Håheim LL, Holme I, Hjermann I, Leren P. Risk factors of stroke incidence and mortality. A 12-year follow-up of the Oslo Study. Stroke. 1993;24(10):1484-9.

63. Iwase H, Tanaka-Mizuno S, Takashima N, Kadota A, Matsui K, Nakamaura Y, et al. Relationship of leisure-time and household physical activity level and type with cardiovascular disease: secondary analysis of the Takashima Study data. BMC Cardiovascular Disorders. 2022;22(1).

64. Li X, Liu S, Mu X, Gao H, Zi Y, Yang H, et al. Association Between Change in Leisure-Time Physical Activity During the Postretirement Period and Incident Stroke. Neurology. 2022.

65. Bergström G, Börjesson M, Schmidt C. Self-efficacy regarding physical activity is superior to self-assessed activity level, in long-term prediction of cardiovascular events in middle-aged men. BMC Public Health. 2015;15(1):1-8.

66. Petersen CB, Grønbæk M, Helge JW, Thygesen LC, Schnohr P, Tolstrup JS. Changes in physical activity in leisure time and the risk of myocardial infarction, ischemic heart disease, and all-cause mortality. European journal of epidemiology. 2012;27:91-9.

67. Zhang YB, Chen C, Pan XF, Guo JY, Li YP, Franco OH, et al. Associations of healthy lifestyle and socioeconomic status with mortality and incident cardiovascular disease: two prospective cohort studies. Bmj-British Medical Journal. 2021;372.

68. Song C, Chang Z, Magnusson PK, Ingelsson E, Pedersen NL. Genetic factors may play a prominent role in the development of coronary heart disease dependent on important environmental factors. J Intern Med. 2014;275(6):631-9.

69. Arsenault BJ, Rana JS, Lemieux I, Després JP, Kastelein JJ, Boekholdt SM, et al. Physical inactivity, abdominal obesity and risk of coronary heart disease in apparently healthy men and women. Int J Obes (Lond). 2010;34(2):340-7.

70. Kiely DK, Wolf PA, Cupples LA, Beiser AS, Kannel WB. Physical activity and stroke risk: the Framingham Study. Am J Epidemiol. 1994;140(7):608-20.

71. Khaw KT, Jakes R, Bingham S, Welch A, Luben R, Day N, et al. Work and leisure time physical activity assessed using a simple, pragmatic, validated questionnaire and incident cardiovascular disease and all-cause mortality in men and women: The European Prospective Investigation into Cancer in Norfolk prospective population study. Int J Epidemiol. 2006;35(4):1034-43.

72. Fraser GE, Strahan TM, Sabate J, Beeson WL, Kissinger D. Effects of traditional coronary risk factors on rates of incident coronary events in a low-risk population. The Adventist Health Study. Circulation. 1992;86(2):406-13.

73. Kubota Y, Iso H, Yamagishi K, Sawada N, Tsugane S, Grp JS. Daily Total Physical Activity and Incident Stroke The Japan Public Health Center-Based Prospective Study. Stroke. 2017;48(7):1730-+.

74. Blond K, Jensen MK, Rasmussen MG, Overvad K, Tjønneland A, Østergaard L, et al. Prospective Study of Bicycling and Risk of Coronary Heart Disease in Danish Men and Women. Circulation. 2016;134(18):1409-11.

75. Williams PT. Dose-response relationship of physical activity to premature and total all-cause and cardiovascular disease mortality in walkers. PLoS One. 2013;8(11):e78777.

76. Hein HO, Suadicani P, Gyntelberg F. Lewis phenotypes, leisure time physical activity, and risk of ischaemic heart disease: an 11 year follow up in the Copenhagen male study. Heart. 2001;85(2):159-64.

77. Williams ED, Stamatakis E, Chandola T, Hamer M. Physical activity behaviour and coronary heart disease mortality among South Asian people in the UK: an observational longitudinal study. Heart. 2011;97(8):655-9.

78. Jackson SE, Brown J, Ussher M, Shahab L, Steptoe A, Smith L. Combined health risks of cigarette smoking and low levels of physical activity: a prospective cohort study in England with 12-year follow-up. BMJ open. 2019;9(11):e032852.

79. MacDonald CJ, Madika A-L, Gomes R, Severi G, Sibon I, Debette S, et al. Physical activity and stroke among women–A non-linear relationship. Preventive Medicine. 2021;150:106485.

80. Iwase H, Tanaka-Mizuno S, Takashima N, Kadota A, Matsui K, Nakamaura Y, et al. Relationship of leisure-time and household physical activity level and type with cardiovascular disease: secondary analysis of the Takashima Study data. BMC Cardiovascular Disorders. 2022;22(1):1-14.

81. Donahue RP, Abbott RD, Reed DM, Yano K. Physical activity and coronary heart disease in middle-aged and elderly men: the Honolulu Heart Program. American Journal of Public Health. 1988;78(6):683-5.

82. Kunutsor SK, Laukkanen JA, Kurl S, Mäkikallio TH, Khan H. Leisure-time cross-country skiing and risk of atrial fibrillation and stroke: A prospective cohort study. European Journal of Preventive Cardiology. 2020;27(19):2354-7.

83. Kenchaiah S, Sesso HD, Gaziano JM. Body mass index and vigorous physical activity and the risk of heart failure among men. Circulation. 2009;119(1):44-52.

84. Qi W, Ma J, Guan T, Zhao D, Abu-Hanna A, Schut M, et al. Risk factors for incident stroke and its subtypes in China: A prospective study. Journal of the American Heart Association. 2020;9(21).

85. Okada H, Horibe H, Yoshiyuki O, Hayakawa N, Aoki N. A prospective study of cerebrovascular disease in Japanese rural communities, Akabane and Asahi. Part 1: evaluation of risk factors in the occurrence of cerebral hemorrhage and thrombosis. Stroke. 1976;7(6):599-607.

86. Ferrario MM, Veronesi G, Chambless LE, Sega R, Fornari C, Bonzini M, et al. The contribution of major risk factors and job strain to occupational class differences in coronary heart disease incidence: the MONICA Brianza and PAMELA population-based cohorts. Occupational and environmental medicine. 2011;68(10):717-22.

87. Millar WJ, Chen J. Health effects of physical activity [1994-95 and 1996-97 data]. Health Reports. 1999;11(1):21.

88. Byrne DW, Rolando LA, Aliyu MH, McGown PW, Connor LR, Awalt BM, et al. Modifiable healthy lifestyle behaviors: 10-year health outcomes from a health promotion program. American journal of preventive medicine. 2016;51(6):1027-37.

89. Cowan LT, Tome J, Mallhi AK, Tarasenko YN, Palta P, Evenson KR, et al. Changes in physical activity and risk of ischemic stroke: The ARIC study. International Journal of Stroke. 2023;18(2):173-9.

90. Feng MY, Wang HX, Zhuo LB, Yao W, Hao CF, Pei JJ. Work-Related Stress and Occurrence of Cardiovascular Disease: A 13-Year Prospective Study. Journal of Occupational and Environmental Medicine. 2022;64(11):927-33.

91. Jae SY, Kim HJ, Kurl S, Kunutsor SK, Laukkanen JA. Independent and Joint Associations of Exercise Blood Pressure and Cardiorespiratory Fitness With the Risk of Cardiovascular Mortality. Am J Hypertens. 2023;36(3):148-50.

92. Ma T, Sirard JR, Jennings L. Association of Time-of-Day Physical Activity With Incident Cardiovascular Disease: The UK Biobank Study. J Phys Act Health. 2023;20(6):547-54.

93. Motamed-Gorji N, Hariri S, Masoudi S, Sharafkhah M, Nalini M, Oveisgharan S, et al. Incidence, early case fatality and determinants of stroke in Iran: Golestan Cohort Study. J Stroke Cerebrovasc Dis. 2022;31(10):106658.

94. Tezuka K, Kubota Y, Ohira T, Muraki I, Hayama-Terada M, Shimizu Y, et al. Modifying Effect of Outdoor Recreational Activity on the Association Between Anger Expression and Cardiovascular Disease Risk: The Circulatory Risk in Communities Study. Psychosom Med. 2023;85(2):182-7.

95. Wang X, Ma H, Li X, Heianza Y, Manson JE, Franco OH, et al. Association of Cardiovascular Health With Life Expectancy Free of Cardiovascular Disease, Diabetes, Cancer, and Dementia in UK Adults. Jama Internal Medicine. 2023;183(4):340-9.

96. Wang Y, Xiao M, Lyu J, Yu C, Guo Y, Pei P, et al. A prospective cohort study of premature death and influencing factors in adults aged 56-69 years from 10 regions of China. Chinese Journal of Endemiology. 2022;43(7):1010-8.

97. Greenlee H, Strizich G, Lovasi GS, Kaplan RC, Biggs ML, Li CI, et al. Concordance with prevention guidelines and subsequent cancer, cardiovascular disease, and mortality: a longitudinal study of older adults. American journal of epidemiology. 2017;186(10):1168-79.

98. Patel K, Sui X, Zhang Y, Fonarow GC, Aban IB, Brown CJ, et al. Prevention of heart failure in older adults may require higher levels of physical activity than needed for other cardiovascular events. Int J Cardiol. 2013;168(3):1905-9.

99. Bell EJ, Lutsey PL, Windham BG, Folsom AR. Physical activity and cardiovascular disease in African Americans in ARIC. Medicine and science in sports and exercise. 2013;45(5):901.

100. Florido R, Zhao D, Ndumele CE, Lutsey PL, McEvoy JW, Windham BG, et al. Physical activity, parental history of premature coronary heart disease, and incident atherosclerotic cardiovascular disease in the atherosclerosis risk in communities (ARIC) study. Journal of the American Heart Association. 2016;5(9):e003505.

101. Porter AK, Schilsky S, Evenson KR, Florido R, Palta P, Holliday KM, et al. The association of sport and exercise activities with cardiovascular disease risk: the Atherosclerosis Risk in Communities (ARIC) study. Journal of Physical Activity and Health. 2019;16(9):698-705.

102. Cuthbertson CC, Tan X, Heiss G, Kucharska‐Newton A, Nichols HB, Kubota Y, et al. Associations of Leisure‐Time Physical Activity and Television Viewing With Life Expectancy Free of Nonfatal Cardiovascular Disease: The ARIC Study. Journal of the American Heart Association. 2019;8(18):e012657.

103. Fletcher G, Alam AB, Li L, Norby FL, Chen LY, Soliman EZ, et al. Association of physical activity with the incidence of atrial fibrillation in persons > 65 years old: the Atherosclerosis Risk in Communities (ARIC) study. BMC Cardiovascular Disorders. 2022;22(1).

104. Chomistek AK, Cook NR, Rimm EB, Ridker PM, Buring JE, Lee IM. Physical Activity and Incident Cardiovascular Disease in Women: Is the Relation Modified by Level of Global Cardiovascular Risk? J Am Heart Assoc. 2018;7(12).

105. Weinstein AR, Sesso HD, Lee I-M, Rexrode KM, Cook NR, Manson JE, et al. The joint effects of physical activity and body mass index on coronary heart disease risk in women. Archives of internal medicine. 2008;168(8):884-90.

106. Everett BM, Conen D, Buring JE, Moorthy M, Lee I-M, Albert CM. Physical activity and the risk of incident atrial fibrillation in women. Circulation: Cardiovascular Quality and Outcomes. 2011;4(3):321-7.

107. Azarbal F, Stefanick ML, Salmoirago-Blotcher E, Manson JE, Albert CM, LaMonte MJ, et al. Obesity, physical activity, and their interaction in incident atrial fibrillation in postmenopausal women. J Am Heart Assoc. 2014;3(4).

108. Chomistek AK, Manson JE, Stefanick ML, Lu B, Sands-Lincoln M, Going SB, et al. Relationship of sedentary behavior and physical activity to incident cardiovascular disease: results from the Women's Health Initiative. J Am Coll Cardiol. 2013;61(23):2346-54.

109. Chomistek AK, Henschel B, Eliassen AH, Mukamal KJ, Rimm EB. Frequency, Type, and Volume of Leisure-Time Physical Activity and Risk of Coronary Heart Disease in Young Women. Circulation. 2016;134(4):290-9.

110. Manson JE, Hu FB, Rich-Edwards JW, Colditz GA, Stampfer MJ, Willett WC, et al. A prospective study of walking as compared with vigorous exercise in the prevention of coronary heart disease in women. N Engl J Med. 1999;341(9):650-8.

111. Elliott EG, Laden F, James P, Rimm EB, Rexrode KM, Hart JE. Interaction between Long-Term Exposure to Fine Particulate Matter and Physical Activity, and Risk of Cardiovascular Disease and Overall Mortality in U.S. Women. Environ Health Perspect. 2020;128(12):127012.

112. Skielboe AK, Marott JL, Dixen U, Friberg JB, Jensen GB. Occupational physical activity, but not leisure-time physical activity increases the risk of atrial fibrillation: The Copenhagen City Heart Study. European Journal of Preventive Cardiology. 2016;23(17):1883-93.

113. Larsson SC, Akesson A, Wolk A. Healthy diet and lifestyle and risk of stroke in a prospective cohort of women. Neurology. 2014;83(19):1699-704.

114. Åkesson A, Weismayer C, Newby P, Wolk A. Combined effect of low-risk dietary and lifestyle behaviors in primary prevention of myocardial infarction in women. Archives of internal medicine. 2007;167(19):2122-7.

115. Allesøe K, Holtermann A, Aadahl M, Thomsen JF, Hundrup YA, Søgaard K. High occupational physical activity and risk of ischaemic heart disease in women: the interplay with physical activity during leisure time. Eur J Prev Cardiol. 2015;22(12):1601-8.

116. Willey JZ, Moon YP, Sacco RL, Greenlee H, Diaz KM, Wright CB, et al. Physical inactivity is a strong risk factor for stroke in the oldest old: Findings from a multi-ethnic population (the Northern Manhattan Study). Int J Stroke. 2017;12(2):197-200.

117. Tanasescu M, Leitzmann MF, Rimm EB, Willett WC, Stampfer MJ, Hu FB. Exercise type and intensity in relation to coronary heart disease in men. Jama. 2002;288(16):1994-2000.

118. Chomistek AK, Cook NR, Flint AJ, Rimm EB. Vigorous-intensity leisure-time physical activity and risk of major chronic disease in men. Medicine and science in sports and exercise. 2012;44(10):1898.

119. Hu G, Tuomilehto J, Borodulin K, Jousilahti P. The joint associations of occupational, commuting, and leisure-time physical activity, and the Framingham risk score on the 10-year risk of coronary heart disease. European Heart Journal. 2007;28(4):492-8.

120. Hu G, Sarti C, Jousilahti P, Silventoinen K, Barengo NC, Tuomilehto J. Leisure time, occupational, and commuting physical activity and the risk of stroke. Stroke. 2005;36(9):1994-9.

121. Blomstrand A, Blomstrand C, Ariai N, Bengtsson C, Björkelund C. Stroke incidence and association with risk factors in women: a 32-year follow-up of the Prospective Population Study of Women in Gothenburg. BMJ Open. 2014;4(10):e005173.

122. Blomstrand A, Blomstrand C, Hakeberg M, Sundh V, Lissner L, Bjorkelund C. Forty-four-year longitudinal study of stroke incidence and risk factors - the Prospective Population Study of Women in Gothenburg. Scandinavian Journal of Primary Health Care. 2022;40(1):139-47.

123. Johansson A, Drake I, Engstrom G, Acosta S. Modifiable and Non-Modifiable Risk Factors for Atherothrombotic Ischemic Stroke among Subjects in the Malmo Diet and Cancer Study. Nutrients. 2021;13(6).

124. Calling S, Hedblad B, Engström G, Berglund G, Janzon L. Effects of body fatness and physical activity on cardiovascular risk: risk prediction using the bioelectrical impedance method. Scand J Public Health. 2006;34(6):568-75.

125. Acosta S, Johansson A, Drake I. Diet and Lifestyle Factors and Risk of Atherosclerotic Cardiovascular Disease-A Prospective Cohort Study. Nutrients. 2021;13(11).

126. Johansson A, Acosta S, Mutie PM, Sonestedt E, Engström G, Drake I. Components of a healthy diet and different types of physical activity and risk of atherothrombotic ischemic stroke: A prospective cohort study. Front Cardiovasc Med. 2022;9:993112.

127. Hidalgo-Santamaria M, Bes-Rastrollo M, Martinez-Gonzalez MA, Moreno-Galarraga L, Ruiz-Canela M, Fernandez-Montero A. Physical activity intensity and cardiovascular disease prevention—from the Seguimiento Universidad De Navarra study. The American Journal of Cardiology. 2018;122(11):1871-8.

128. Clays E, De Bacquer D, Janssens H, De Clercq B, Casini A, Braeckman L, et al. The association between leisure time physical activity and coronary heart disease among men with different physical work demands: a prospective cohort study. Eur J Epidemiol. 2013;28(3):241-7.

129. Clays E, Casini A, Van Herck K, De Bacquer D, Kittel F, De Backer G, et al. Do psychosocial job resources buffer the relation between physical work demands and coronary heart disease? A prospective study among men. Int Arch Occup Environ Health. 2016;89(8):1299-307.

130. Ahmed A, Pinto Pereira SM, Lennon L, Papacosta O, Whincup P, Wannamethee G. Cardiovascular Health and Stroke in Older British Men: Prospective Findings From the British Regional Heart Study. Stroke. 2020;51(11):3286-94.

131. Jefferis BJ, Whincup PH, Lennon LT, Papacosta O, Goya Wannamethee S. Physical activity in older men: longitudinal associations with inflammatory and hemostatic biomarkers, N-terminal pro-brain natriuretic peptide, and onset of coronary heart disease and mortality. J Am Geriatr Soc. 2014;62(4):599-606.

132. Harmsen P, Lappas G, Rosengren A, Wilhelmsen L. Long-term risk factors for stroke: twenty-eight years of follow-up of 7457 middle-aged men in Goteborg, Sweden. Stroke. 2006;37(7):1663-7.

133. Veronesi G, Borchini R, Landsbergis P, Iacoviello L, Gianfagna F, Tayoun P, et al. Cardiovascular disease prevention at the workplace: assessing the prognostic value of lifestyle risk factors and job-related conditions. Int J Public Health. 2018;63(6):723-32.

134. Ferrario MM, Roncaioli M, Veronesi G, Holtermann A, Clays E, Borchini R, et al. Differing associations for sport versus occupational physical activity and cardiovascular risk. Heart. 2018;104(14):1165-72.

135. Lakka TA, Venalainen JM, Rauramaa R, Salonen R, Tuomilehto J, Salonen JT. Relation of leisure-time physical activity and cardiorespiratory fitness to the risk of acute myocardial infarction in men. New England Journal of Medicine. 1994;330(22):1549-54.

136. Willey JZ, Voutsinas J, Sherzai A, Ma H, Bernstein L, Elkind MS, et al. Trajectories in leisure-time physical activity and risk of stroke in women in the California teachers study. Stroke. 2017;48(9):2346-52.

137. Agnarsson U, Thorgeirsson G, Sigvaldason H, Sigfusson N. Effects of leisure-time physical activity and ventilatory function on risk for stroke in men: the Reykjavik Study. Annals of Internal Medicine. 1999;130(12):987-90.

138. Lear SA, Hu W, Rangarajan S, Gasevic D, Leong D, Iqbal R, et al. The effect of physical activity on mortality and cardiovascular disease in 130 000 people from 17 high-income, middle-income, and low-income countries: the PURE study. The Lancet. 2017;390(10113):2643-54.

139. Wagner A, Simon C, Evans A, Ferrières J, Montaye M, Ducimetière P, et al. Physical activity and coronary event incidence in Northern Ireland and France: the Prospective Epidemiological Study of Myocardial Infarction (PRIME). Circulation. 2002;105(19):2247-52.

140. Petersen CB, Eriksen L, Tolstrup JS, Søgaard K, Grønbæk M, Holtermann A. Occupational heavy lifting and risk of ischemic heart disease and all-cause mortality. BMC public health. 2012;12:1-9.

141. Merry AH, Boer JM, Schouten LJ, Feskens EJ, Verschuren WM, Gorgels AP, et al. Smoking, alcohol consumption, physical activity, and family history and the risks of acute myocardial infarction and unstable angina pectoris: a prospective cohort study. BMC cardiovascular disorders. 2011;11:1-14.

142. Rosolova H, Simon J, Sefrna F. Impact of cardiovascular risk factors on morbidity and mortality in Czech middle-aged men: Pilsen Longitudinal Study. Cardiology. 1994;85(1):61-8.

143. Kaprio J, Kujala UM, Koskenvuo M, Sarna S. Physical activity and other risk factors in male twin-pairs discordant for coronary heart disease. Atherosclerosis. 2000;150(1):193-200.

144. Kuster RP, von Rosen P, Grooten WJ, Dohrn I-M, Hagströmer M. Self-Reported and Device-Measured Physical Activity in Leisure Time and at Work and Associations with Cardiovascular Events—A Prospective Study of the Physical Activity Paradox. International Journal of Environmental Research and Public Health. 2021;18(22):12214.

145. Paudel S, Ahmadi M, Phongsavan P, Hamer M, Stamatakis E. Do associations of physical activity and sedentary behaviour with cardiovascular disease and mortality differ across socioeconomic groups? A prospective analysis of device-measured and self-reported UK Biobank data. British Journal of Sports Medicine. 2023.

146. Garnvik LE, Malmo V, Janszky I, Wisløff U, Loennechen JP, Nes BM. Physical activity modifies the risk of atrial fibrillation in obese individuals: the HUNT3 study. European Journal of Preventive Cardiology. 2018;25(15):1646-52.

147. Johansen KR, Ranhoff AH, Sørensen E, Nes BM, Heitmann KA, Apelland T, et al. Risk of atrial fibrillation and stroke among older men exposed to prolonged endurance sport practice: a 10-year follow-up. The Birkebeiner Ageing Study and the Tromsø Study. Open Heart. 2022;9(2).

148. Gillum RF, Mussolino ME, Ingram DD. Physical activity and stroke incidence in women and men: the NHANES I Epidemiologic Follow-up Study. American journal of epidemiology. 1996;143(9):860-9.

149. Hu G-C, Chien K-L, Hsieh S-F, Chen C-Y, Tsai W-H, Su T-C. Occupational versus leisure-time physical activity in reducing cardiovascular risks and mortality among ethnic Chinese adults in Taiwan. Asia Pacific Journal of Public Health. 2014;26(6):604-13.

150. Hummel M, Hantikainen E, Adami H-O, Ye W, Bellocco R, Bonn SE, et al. Association between total and leisure time physical activity and risk of myocardial infarction and stroke–a Swedish cohort study. BMC Public Health. 2022;22(1):1-15.

151. Saevereid HA, Schnohr P, Prescott E. Speed and duration of walking and other leisure time physical activity and the risk of heart failure: A prospective cohort study from the Copenhagen City Heart Study. PLoS ONE. 2014;9(3).

152. Strippoli E, Hughes A, Sebastiani G, Di Filippo P, d’Errico A. Occupational physical activity, mortality and CHD events in the Italian Longitudinal Study. International Archives of Occupational and Environmental Health. 2021:1-13.

153. Sundquist K, Qvist J, Johansson S-E, Sundquist J. The long-term effect of physical activity on incidence of coronary heart disease: a 12-year follow-up study. Preventive medicine. 2005;41(1):219-25.

154. Zhao H, Zhang XN, Shi Z, Yin L, Zhang WL, He K, et al. Association of level of leisure-time physical activity with risks of all-cause mortality and cardiovascular disease in an elderly Chinese population: a prospective cohort study. J Geriatr Cardiol. 2020;17(10):628-37.

155. Amidei CB, Trevisan C, Dotto M, Ferroni E, Noale M, Maggi S, et al. Association of physical activity trajectories with major cardiovascular diseases in elderly people. Heart. 2022;108(5):360-6.

156. Armstrong ME, Green J, Reeves GK, Beral V, Cairns BJ. Frequent physical activity may not reduce vascular disease risk as much as moderate activity: large prospective study of women in the United Kingdom. Circulation. 2015;131(8):721-9.

157. Barengo NC, Antikainen R, Borodulin K, Harald K, Jousilahti P. Leisure‐time physical activity reduces total and cardiovascular mortality and cardiovascular disease incidence in older adults. Journal of the American Geriatrics Society. 2017;65(3):504-10.

158. Bakker EA, Lee DC, Hopman MTE, Oymans EJ, Watson PM, Thompson PD, et al. Dose-response association between moderate to vigorous physical activity and incident morbidity and mortality for individuals with a different cardiovascular health status: A cohort study among 142,493 adults from the Netherlands. PLoS Med. 2021;18(12):e1003845.

159. Clara A, Berenguer G, Pérez-Fernández S, Schröder H, Ramos R, Grau M, et al. Analysis of the dose-response relationship of leisure-time physical activity to cardiovascular disease and all-cause mortality: the REGICOR study. Revista Española de Cardiología (English Edition). 2021;74(5):414-20.

160. Ekblom-Bak E, Halldin M, Vikström M, Stenling A, Gigante B, de Faire U, et al. Physical activity attenuates cardiovascular risk and mortality in men and women with and without the metabolic syndrome–a 20-year follow-up of a population-based cohort of 60-year-olds. European journal of preventive cardiology. 2021;28(12):1376-85.

161. Elwood P, Galante J, Pickering J, Palmer S, Bayer A, Ben-Shlomo Y, et al. Healthy lifestyles reduce the incidence of chronic diseases and dementia: evidence from the Caerphilly cohort study. PloS one. 2013;8(12):e81877.

162. Fernandez-Lazaro CI, Sayon-Orea C, Toledo E, Moreno-Iribas C, Guembe MJ. Association of ideal cardiovascular health with cardiovascular events and risk advancement periods in a Mediterranean population-based cohort. BMC Med. 2022;20(1):232.

163. Ford ES, Bergmann MM, Kröger J, Schienkiewitz A, Weikert C, Boeing H. Healthy living is the best revenge: findings from the European Prospective Investigation Into Cancer and Nutrition-Potsdam study. Arch Intern Med. 2009;169(15):1355-62.

164. Fu M, Rosengren A, Thunström E, Mandalenakis Z, Welin L, Caidahl K, et al. Although Coronary Mortality Has Decreased, Rates of Cardiovascular Disease Remain High: 21 Years of Follow‐Up Comparing Cohorts of Men Born in 1913 With Men Born in 1943. Journal of the American Heart Association. 2018;7(9):e008769.

165. Gunnell AS, Knuiman MW, Divitini ML, Cormie P. Leisure time physical activity and long-term cardiovascular and cancer outcomes: the Busselton Health Study. European journal of epidemiology. 2014;29(11):851-7.

166. Hoevenaar-Blom MP, Wendel-Vos GW, Spijkerman AM, Kromhout D, Verschuren WM. Cycling and sports, but not walking, are associated with 10-year cardiovascular disease incidence: the MORGEN Study. European Journal of Preventive Cardiology. 2011;18(1):41-7.

167. Holtermann A, Schnohr P, Nordestgaard BG, Marott JL. The physical activity paradox in cardiovascular disease and all-cause mortality: the contemporary Copenhagen General Population Study with 104 046 adults. European heart journal. 2021;42(15):1499-511.

168. Jasiukaitienė V, Lukšienė D, Tamošiūnas A, Radišauskas R, Bobak M. The impact of metabolic syndrome and lifestyle habits on the risk of the first event of cardiovascular disease: Results from a cohort study in lithuanian urban population. Medicina. 2020;56(1):18.

169. Lacey B, Golledge J, Yeap BB, Lewington S, Norman PE, Flicker L, et al. Physical activity and vascular disease in a prospective cohort study of older men: The Health In Men Study (HIMS). BMC geriatrics. 2015;15:1-9.

170. Meneton P, Lemogne C, Herquelot E, Bonenfant S, Larson MG, Vasan RS, et al. A global view of the relationships between the main behavioural and clinical cardiovascular risk factors in the GAZEL prospective cohort. PLoS One. 2016;11(9):e0162386.

171. Mu X, Yu K, Long P, Niu R, Li W, Chen H, et al. Leisure-time physical activity and risk of incident cardiovascular disease in Chinese retired adults. Scientific Reports. 2021;11(1):24202.

172. Sesso HD, Paffenbarger RS, Ha T, Lee I-M. Physical activity and cardiovascular disease risk in middle-aged and older women. American journal of epidemiology. 1999;150(4):408-16.

173. Stamatakis E, Hamer M, Lawlor DA. Physical activity, mortality, and cardiovascular disease: is domestic physical activity beneficial? The Scottish Health Survey—1995, 1998, and 2003. American journal of epidemiology. 2009;169(10):1191-200.

174. Thomas IC, Takemoto ML, Forbang NI, Larsen BA, Michos ED, McClelland RL, et al. Associations of recreational and non-recreational physical activity with coronary artery calcium density vs. volume and cardiovascular disease events: the Multi-Ethnic Study of Atherosclerosis. Eur Heart J Cardiovasc Imaging. 2020;21(2):132-40.

175. van Sloten TT, Tafflet M, Périer M-C, Dugravot A, Climie RE, Singh-Manoux A, et al. Association of change in cardiovascular risk factors with incident cardiovascular events. Jama. 2018;320(17):1793-804.

176. Yang PS, Jang E, Yu HT, Kim TH, Pak HN, Lee MH, et al. Changes in cardiovascular risk factors and cardiovascular events in the elderly population. Journal of the American Heart Association. 2021;10(11):e019482.

177. Åkesson A, Larsson SC, Discacciati A, Wolk A. Low-risk diet and lifestyle habits in the primary prevention of myocardial infarction in men: a population-based prospective cohort study. Journal of the American college of cardiology. 2014;64(13):1299-306.

178. Haapanen N, Miilunpalo S, Vuori I, Oja P, Pasanen M. Association of leisure time physical activity with the risk of coronary heart disease, hypertension and diabetes in middle-aged men and women. International journal of epidemiology. 1997;26(4):739-47.

179. Jensen MK, Chiuve SE, Rimm EB, Dethlefsen C, Tjønneland A, Joensen AM, et al. Obesity, behavioral lifestyle factors, and risk of acute coronary events. Circulation. 2008;117(24):3062-9.

180. Kim Y, Sharp S, Hwang S, Jee SH. Exercise and incidence of myocardial infarction, stroke, hypertension, type 2 diabetes and site-specific cancers: prospective cohort study of 257 854 adults in South Korea. BMJ open. 2019;9(3):e025590.

181. Koolhaas CM, Dhana K, Golubic R, Schoufour JD, Hofman A, van Rooij FJ, et al. Physical activity types and coronary heart disease risk in middle-aged and elderly persons: the Rotterdam Study. American journal of epidemiology. 2016;183(8):729-38.

182. Raza W, Krachler B, Forsberg B, Sommar JN. Air pollution, physical activity and ischaemic heart disease: a prospective cohort study of interaction effects. BMJ Open. 2021;11(4):e040912.

183. Renninger M, Løchen M-L, Ekelund U, Hopstock LA, Jørgensen L, Mathiesen EB, et al. The independent and joint associations of physical activity and body mass index with myocardial infarction: The Tromsø Study. Preventive medicine. 2018;116:94-8.

184. Sesso HD, Paffenbarger Jr RS, Lee I-M. Physical activity and coronary heart disease in men: The Harvard Alumni Health Study. Circulation. 2000;102(9):975-80.

185. Hall C, Heck JE, Sandler DP, Ritz B, Chen H, Krause N. Occupational and leisure-time physical activity differentially predict 6-year incidence of stroke and transient ischemic attack in women. Scandinavian journal of work, environment & health. 2019;45(3):267.

186. Huerta JM, Chirlaque M-D, Tormo M-J, Gavrila D, Arriola L, Moreno-Iribas C, et al. Physical activity and risk of cerebrovascular disease in the European Prospective Investigation into Cancer and Nutrition-Spain study. Stroke. 2013;44(1):111-8.

187. Jeong HG, Kim DY, Kang DW, Kim BJ, Kim CK, Kim Y, et al. Physical activity frequency and the risk of stroke: a nationwide cohort study in Korea. Journal of the American Heart Association. 2017;6(9):e005671.

188. Lee I-M, Paffenbarger Jr RS. Physical activity and stroke incidence: the Harvard Alumni Health Study. Stroke. 1998;29(10):2049-54.

189. Rist PM, Lee IM, Kase CS, Gaziano JM, Kurth T. Physical activity and functional outcomes from cerebral vascular events in men. Stroke. 2011;42(12):3352-6.

190. Aizer A, Gaziano JM, Cook NR, Manson JE, Buring JE, Albert CM. Relation of vigorous exercise to risk of atrial fibrillation. The American journal of cardiology. 2009;103(11):1572-7.

191. Albrecht M, Koolhaas CM, Schoufour JD, van Rooij FJ, Kavousi M, Ikram MA, et al. Physical activity types and atrial fibrillation risk in the middle-aged and elderly: The Rotterdam Study. European journal of preventive cardiology. 2018;25(12):1316-23.

192. Drca N, Wolk A, Jensen-Urstad M, Larsson SC. Atrial fibrillation is associated with different levels of physical activity levels at different ages in men. Heart. 2014;100(13):1037-42.

193. Jin M-N, Yang P-S, Song C, Yu HT, Kim T-H, Uhm J-S, et al. Physical activity and risk of atrial fibrillation: a nationwide cohort study in general population. Scientific reports. 2019;9(1):1-9.

194. Knuiman M, Briffa T, Divitini M, Chew D, Eikelboom J, McQuillan B, et al. A cohort study examination of established and emerging risk factors for atrial fibrillation: the Busselton Health Study. European journal of epidemiology. 2014;29(3):181-90.

195. Mokhayeri Y, Hashemi-Nazari SS, Mansournia MA, Soori H, Khodakarim S. The association between physical activity and atrial fibrillation applying the Heaviside function in survival analysis: the Multi-Ethnic Study of Atherosclerosis. Epidemiology and Health. 2017;39.

196. Bennett DA, Du H, Clarke R, Guo Y, Yang L, Bian Z, et al. Association of physical activity with risk of major cardiovascular diseases in Chinese men and women. JAMA cardiology. 2017;2(12):1349-58.

197. Allesøe K, Aadahl M, Jacobsen RK, Kårhus LL, Mortensen OS, Korshøj M. Prospective relationship between occupational physical activity and risk of ischaemic heart disease: are men and women differently affected? Eur J Prev Cardiol. 2023;30(9):858-67.

198. Holtermann A, Marott JL, Gyntelberg F, Søgaard K, Suadicani P, Mortensen OS, et al. Occupational and leisure time physical activity: risk of all-cause mortality and myocardial infarction in the Copenhagen City Heart Study. A prospective cohort study. BMJ open. 2012;2(1):e000556.

199. Johnsen AM, Alfredsson L, Knutsson A, Westerholm PJ, Fransson EI. Association between occupational physical activity and myocardial infarction: a prospective cohort study. BMJ open. 2016;6(10):e012692.

200. Krause N, Brand RJ, Arah OA, Kauhanen J. Occupational physical activity and 20-year incidence of acute myocardial infarction: results from the Kuopio Ischemic Heart Disease Risk Factor Study. Scandinavian journal of work, environment & health. 2015:124-39.
