# Supplementary material for: Leisure-time and occupational physical activity and risk of cardiovascular disease incidence: a systematic-review and dose-response meta-analysis of prospective cohort studies
Source: Int J Behav Nutr Phys Act. 2024 Apr 24;21:45. doi: 10.1186/s12966-024-01593-8 (PMC11044601; doi:10.1186/s12966-024-01593-8)
Supplement: Supplementary file 2 — Supplementary Material 2 [file 12966_2024_1593_MOESM2_ESM.docx]

**Leisure-time and occupational physical activity and cardiovascular disease incidence: a systematic review and dose-response meta-analysis of prospective cohort studies**

Supplementary Figure 1. Funnel plots showing study precision against the relative risk with 95% confidence intervals for leisure time physical activity (high vs. low analysis) and cardiovascular incidence


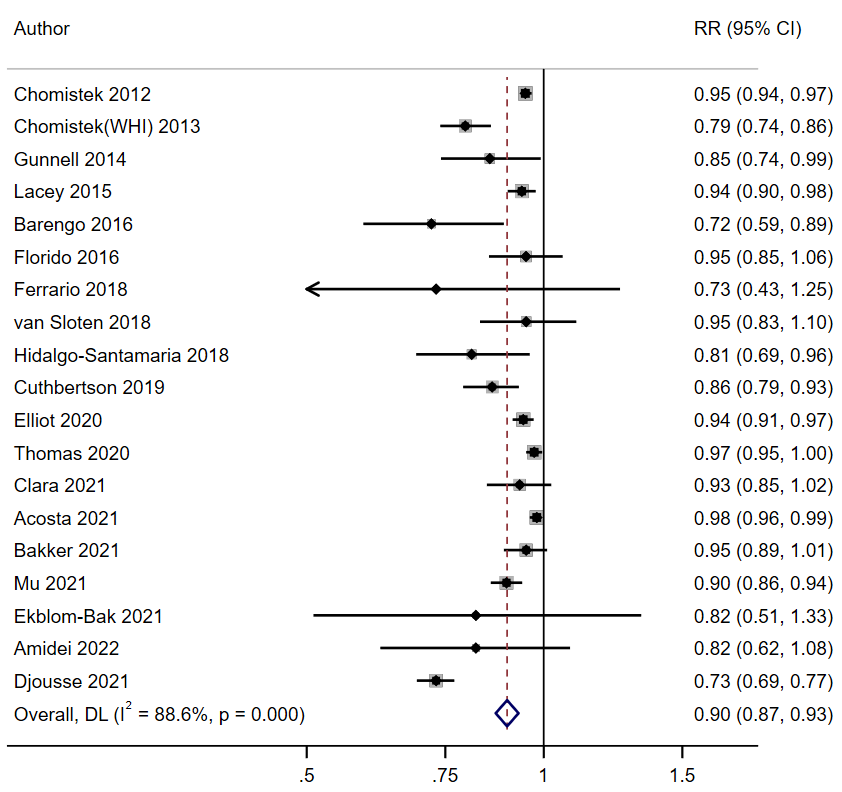


Supplementary Figure 2. Summary hazard ratio of cardiovascular disease incidence for 20 Met-hour/week increment in leisure time physical activity

| 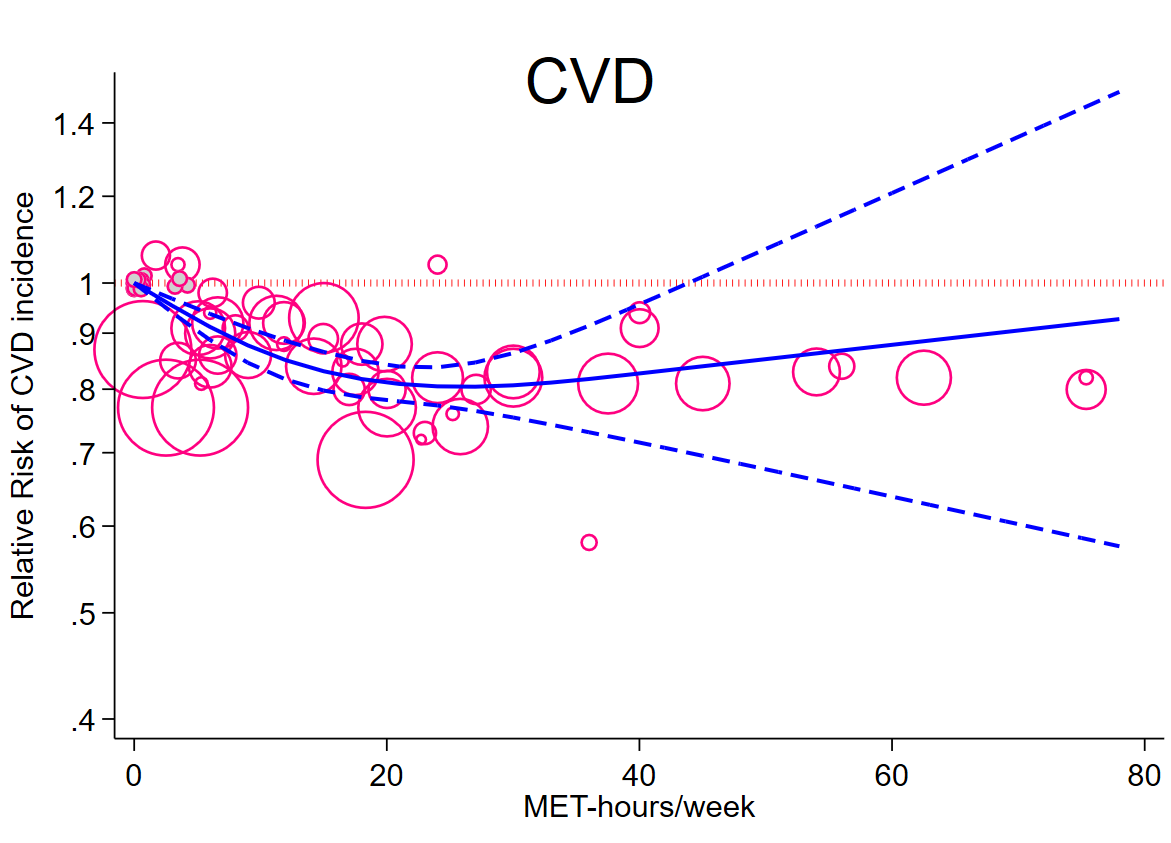 | 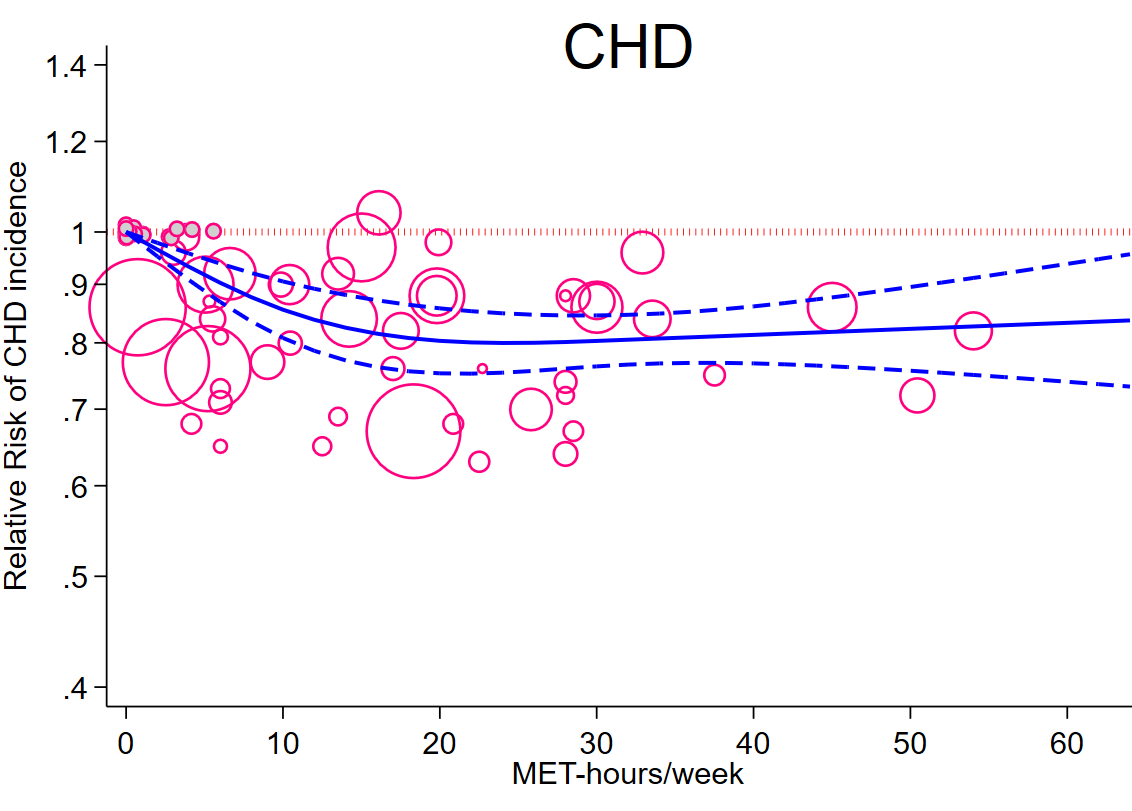 |
| --- | --- |
| 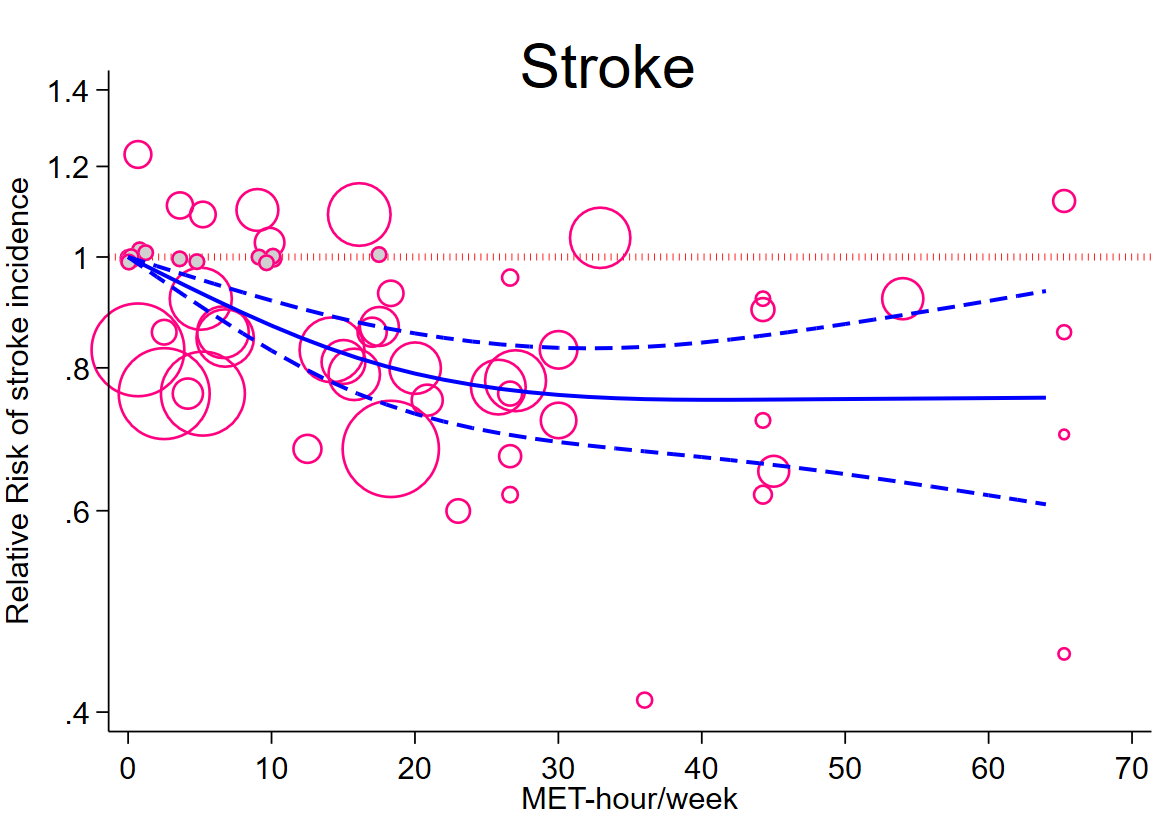 | 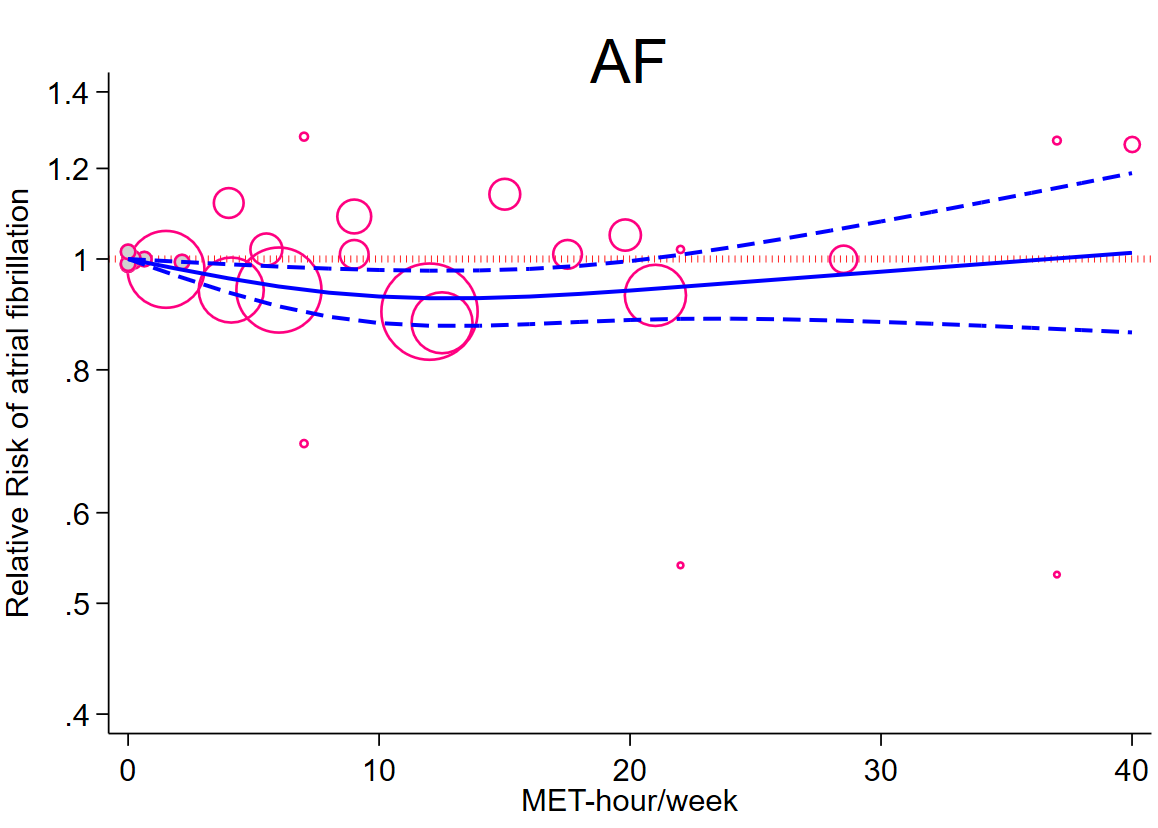 |

Supplementary Figure 3. Dose-response relationship between leisure time physical activity (LTPA) and cardiovascular disease (CVD), coronary heart disease (CHD), stoke, and atrial fibrillation (AF) after excluding the studies with two categories. Solid line represents non-linear dose response and dotted lines represent 95% confidence interval. Circles represent hazard ratio point estimates for LTPA categories from each study with circle size proportional to inverse of standard error. Small grey circles represent baseline LTPA category for each separate study.

Supplementary Figure 4. Dose-response relationship between leisure time physical activity (LTPA) in kcal/week and risk of cardiovascular disease. Solid line represents non-linear dose response and dotted lines represent the 95% confidence intervals. Circles represent hazard ratio point estimates for LTPA categories from each study with circle size proportional to inverse of standard error. Small grey circles represent baseline LTPA category for each separate study.


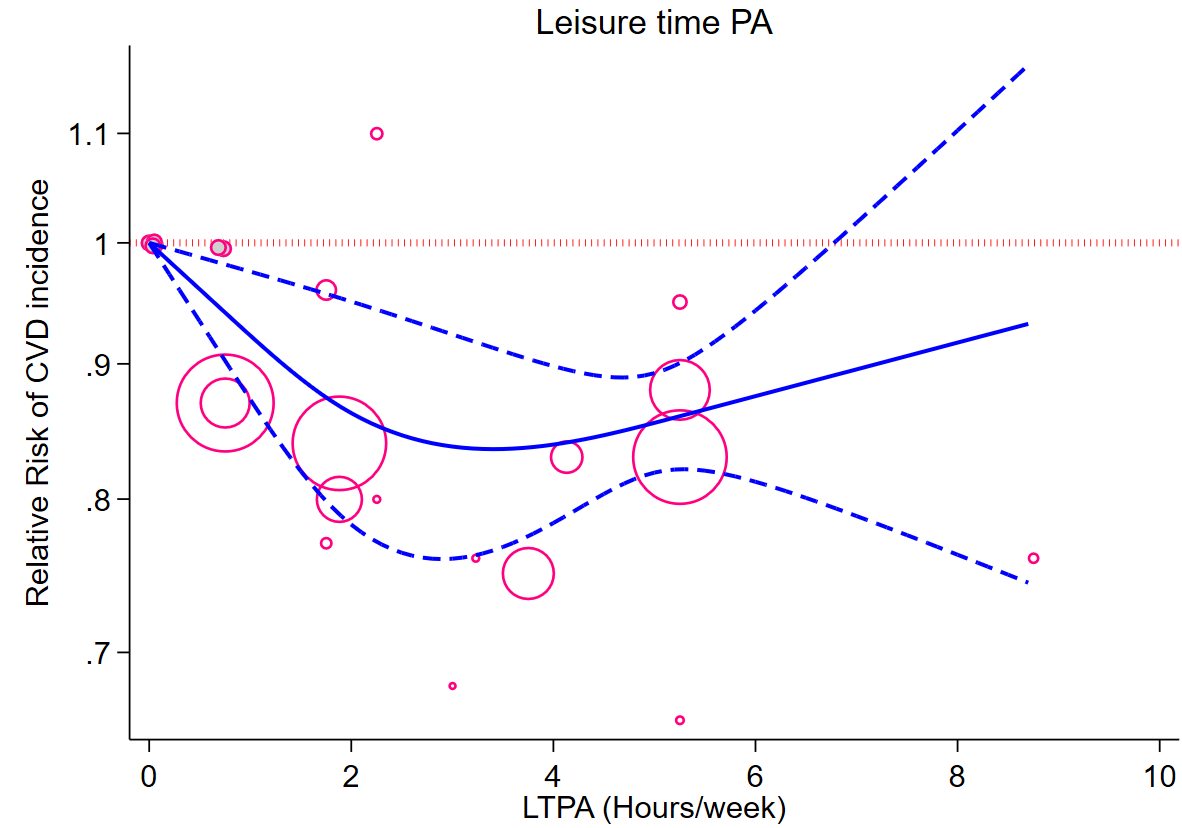


Supplementary Figure 5. Dose-response relationship between leisure time physical activity (LTPA) in hour/week and risk of cardiovascular disease. Solid line represents non-linear dose response and dotted lines represent 95% confidence interval. Circles represent hazard ratio point estimates for LTPA categories from each study with circle size proportional to inverse of standard error. Small grey circles represent baseline LTPA category for each separate study.

Supplementary Figure 6. Funnel plots showing study precision against the relative risk with 95% confidence intervals for leisure time physical activity (high vs. low analysis) and coronary heart disease incidence


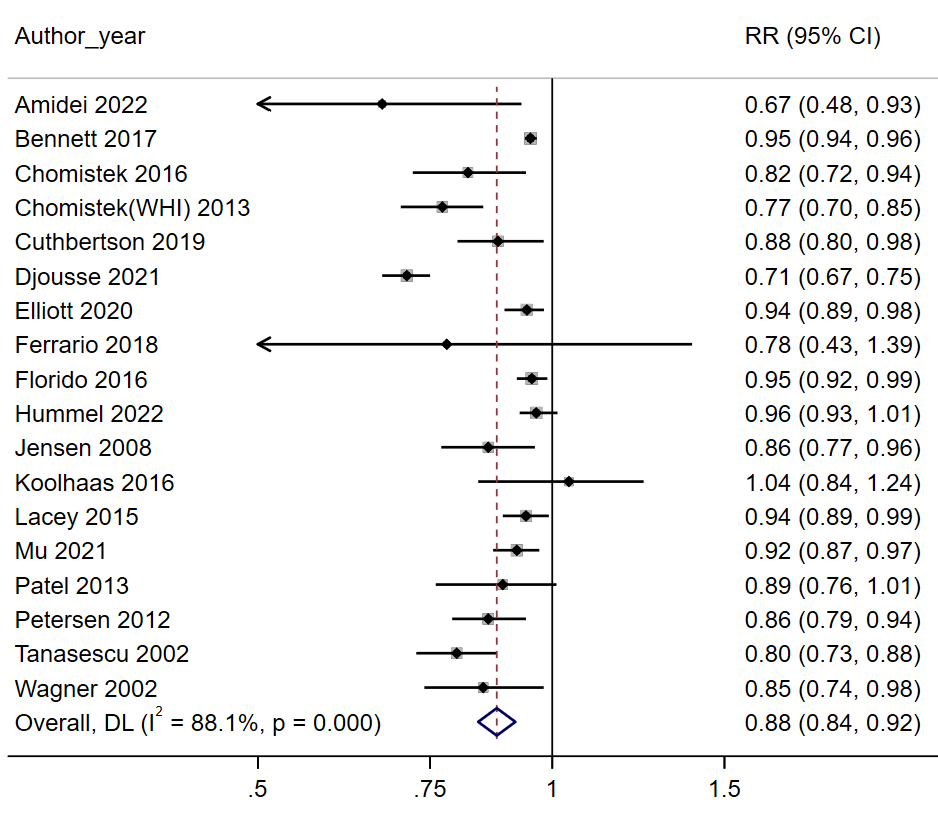


Supplementary Figure 7. Summary hazard ratio of coronary heart disease incidence for 20 Met-hour/week increment in leisure time physical activity

Supplementary Figure 8. Dose-response relationship between leisure time physical activity (LTPA) and risk of coronary heart disease. Solid line represents non-linear dose response and dotted lines represent 95% confidence interval. Circles represent hazard ratio point estimates for LTPA categories from each study with circle size proportional to inverse of standard error. Small grey circles represent baseline LTPA category for each separate study.


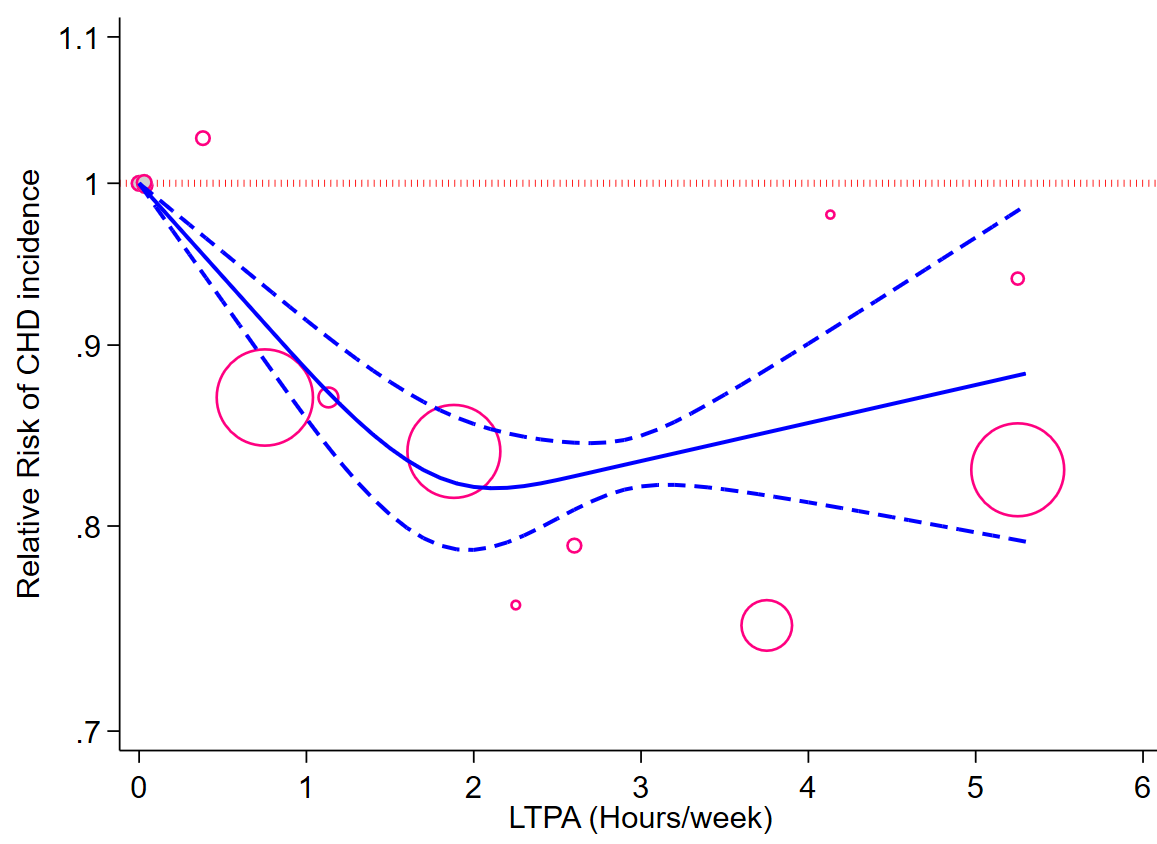


Supplementary Figure 9. Dose-response relationship between leisure time physical activity (LTPA) and risk of coronary heart disease. Solid line represents non-linear dose response and dotted lines represent 95% confidence interval. Circles represent hazard ratio point estimates for LTPA categories from each study with circle size proportional to inverse of standard error. Small grey circles represent baseline LTPA category for each separate study.

-

Supplementary Figure 10. Funnel plots showing study precision against the relative risk with 95% confidence intervals for leisure time physical activity (high vs. low analysis) and stroke incidence

Supplementary Figure 11. Summary hazard ratio of stroke incidence for 20 Met-hour/week increment in leisure time physical activity

Supplementary Figure 12. Dose-response relationship between leisure time physical activity (LTPA) in kcal/week and risk of stroke. Solid line represents non-linear dose response and dotted lines represent 95% confidence interval. Circles represent hazard ratio point estimates for LTPA categories from each study with circle size proportional to inverse of standard error. Small grey circles represent baseline LTPA category for each separate study.


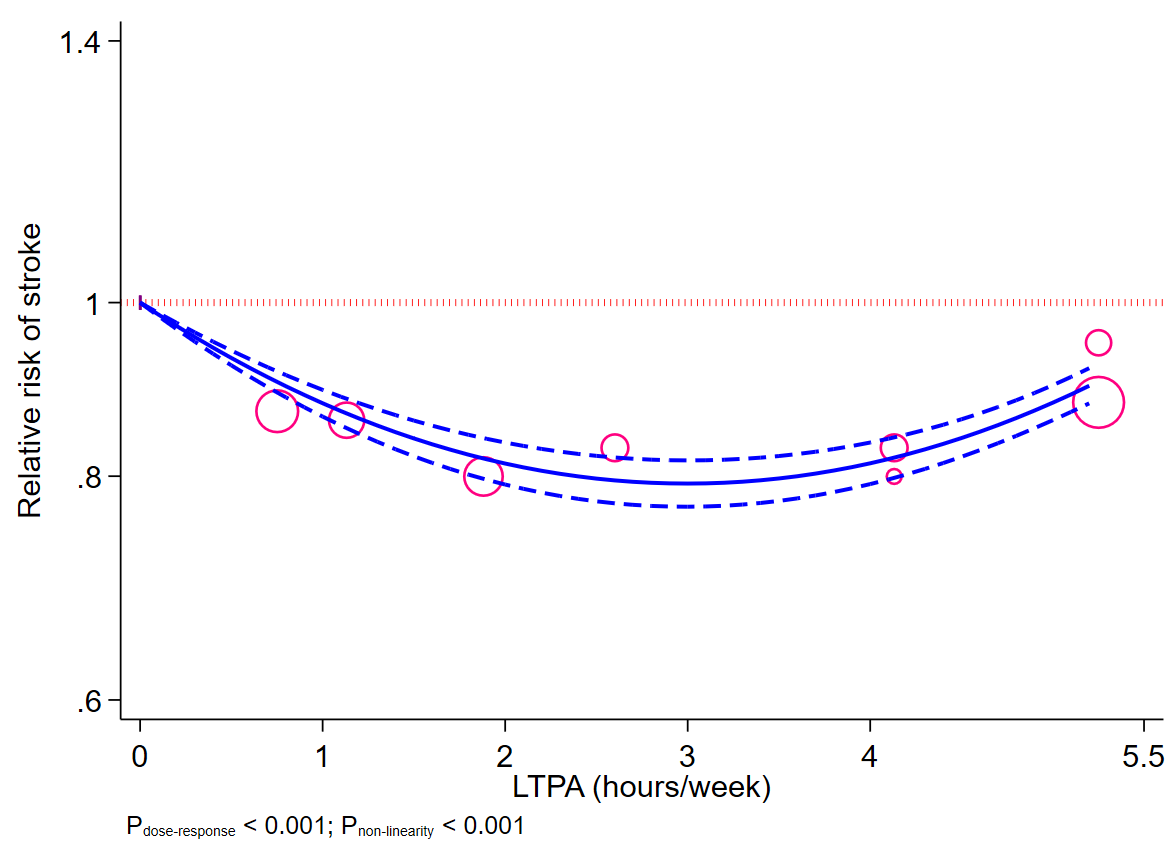


Supplementary Figure 13. Dose-response relationship between leisure time physical activity (LTPA) in hours/week and risk of stroke. Solid line represents non-linear dose response and dotted lines represent 95% confidence interval. Circles represent hazard ratio point estimates for LTPA categories from each study with circle size proportional to inverse of standard error. Small grey circles represent baseline LTPA category for each separate study.

Supplementary Figure 14. Funnel plots showing study precision against the relative risk with 95% confidence intervals for leisure time physical activity (high vs. low analysis) and atrial fibrillation incidence


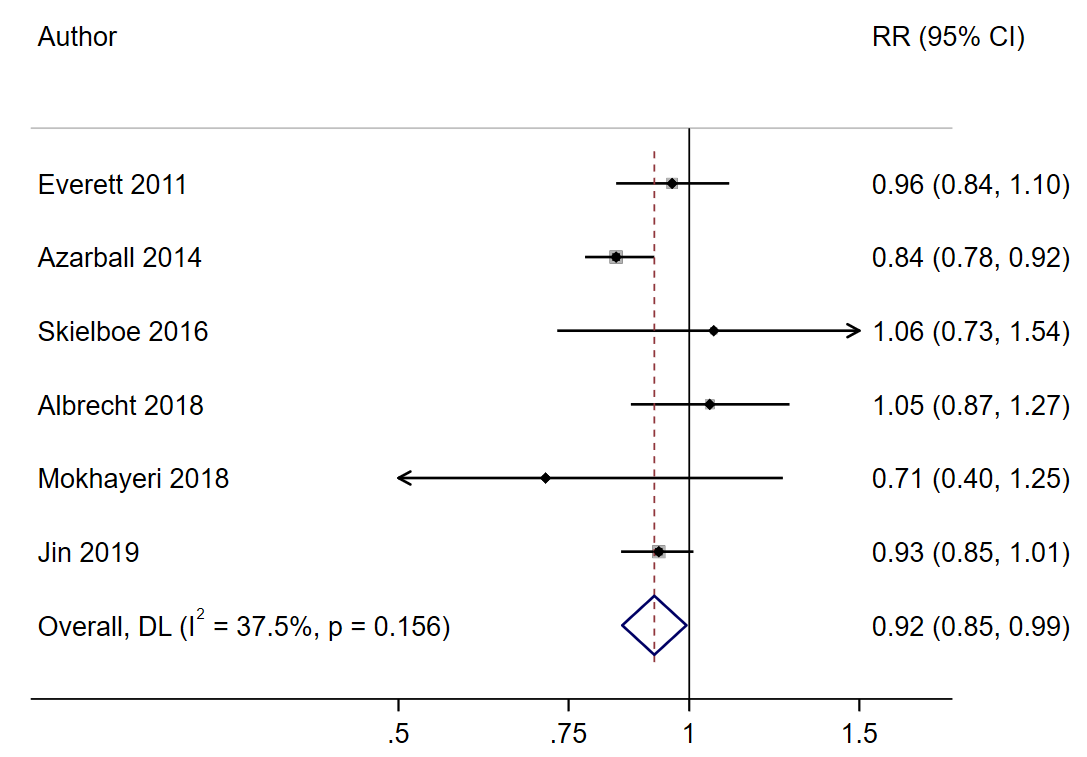


Supplementary Figure 15. Summary hazard ratio of atrial fibrillation incidence for 20 Met-hour/week increment in leisure time physical activity


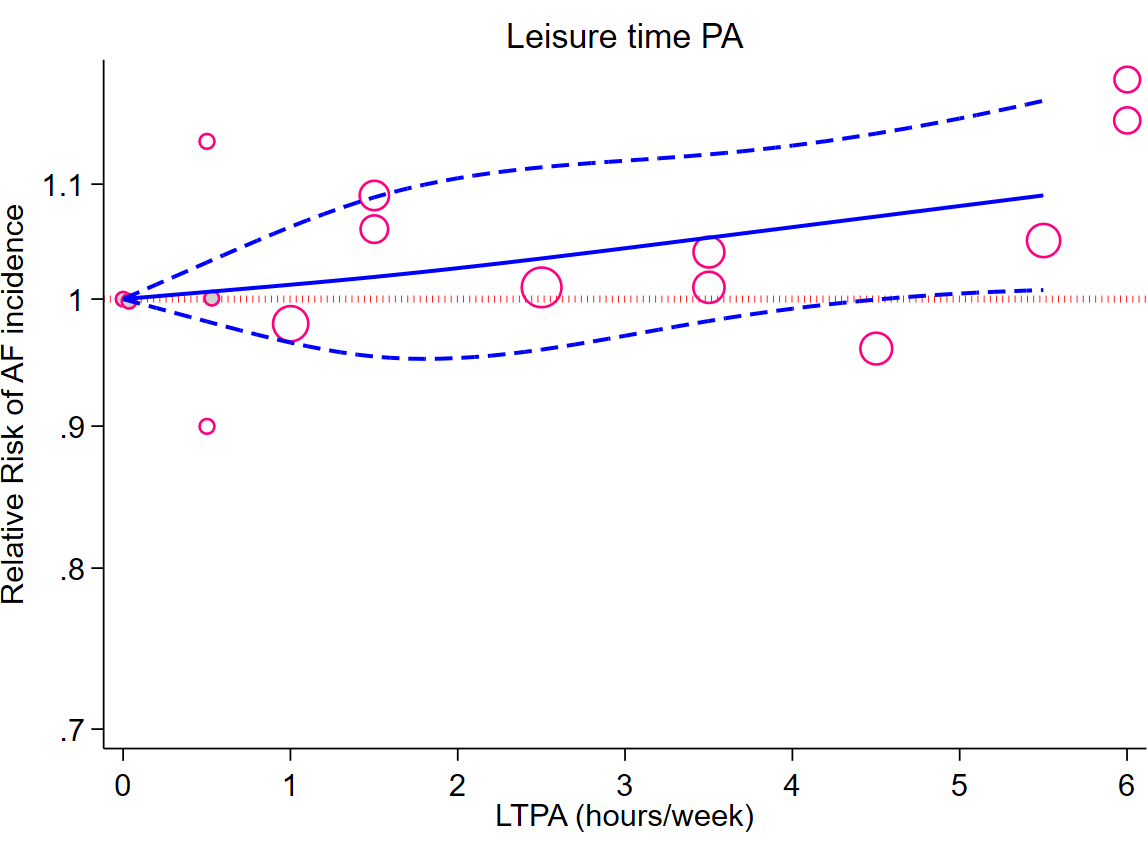


Supplementary Figure 16. Dose-response relationship between leisure time physical activity (LTPA) in hours/week and risk of atrial fibrillation (AF). Solid line represents non-linear dose response and dotted lines represent 95% confidence interval. Circles represent hazard ratio point estimates for LTPA categories from each study with circle size proportional to inverse of standard error. Small grey circles represent baseline LTPA category for each separate study.


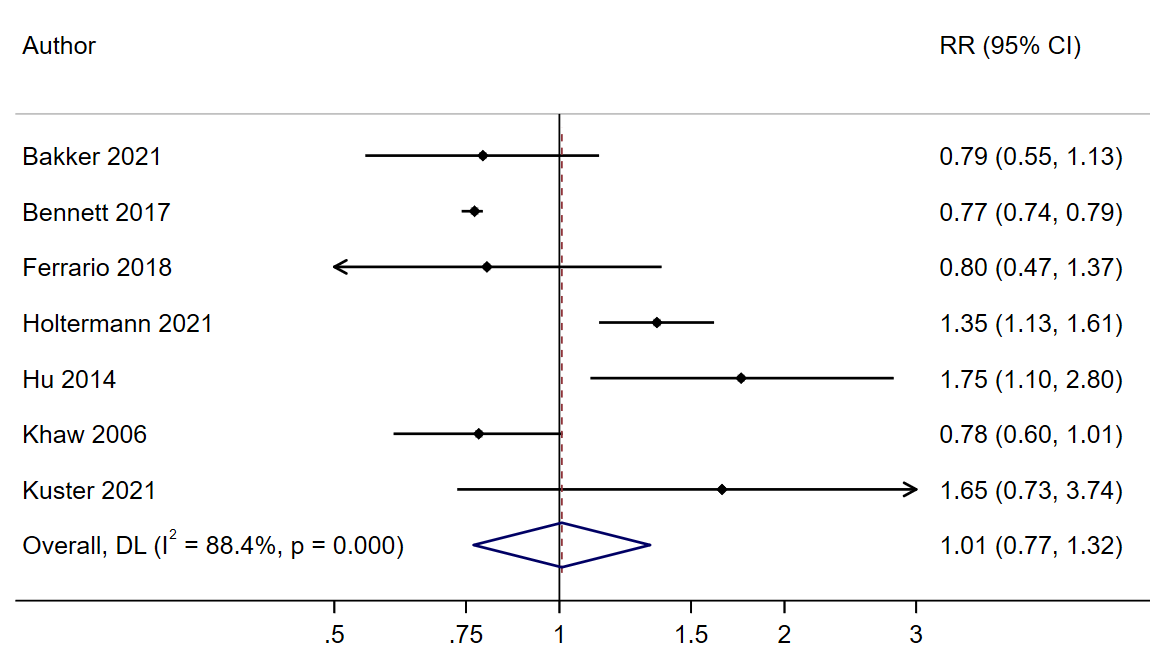


Supplementary Figure 17. Summary hazard ratio of cardiovascular disease incidence for the highest compared with the lowest category of occupational physical activity

Supplementary Figure 18. Summary hazard ratio of coronary heart disease incidence for the highest compared with the lowest category of occupational physical activity

Supplementary Figure 19. Summary hazard ratio of stroke incidence for the highest compared with the lowest category of occupational physical activity


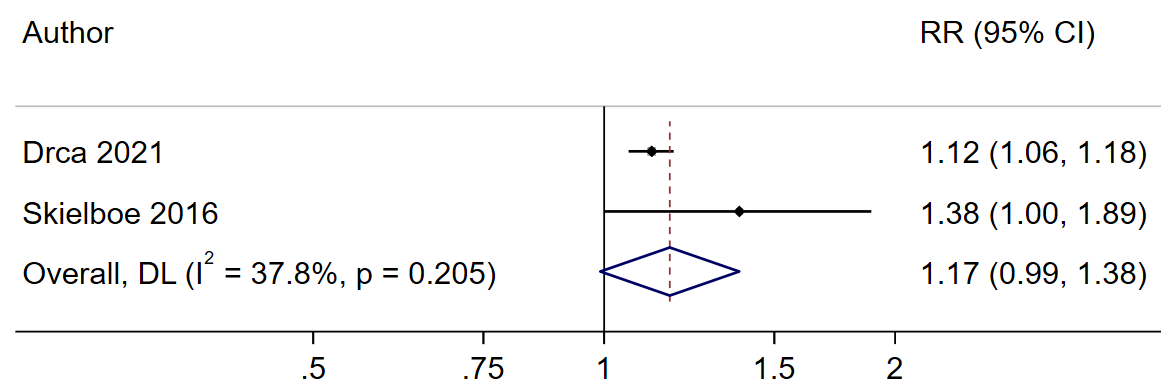


Supplementary Figure 20. Summary hazard ratio of atrial fibrillation for the highest compared with the lowest category of occupational physical activity

Supplementary Figure 21. Funnel plots showing study precision against the relative risk with 95% confidence intervals for occupational physical activity (high vs. low analysis) and coronary heart disease incidence
